# N-Myristoytransferase Inhibition Causes Mitochondrial Iron Overload and Parthanatos in TIM17A-Dependent Aggressive Lung Carcinoma

**DOI:** 10.1158/2767-9764.CRC-23-0428

**Published:** 2024-07-25

**Authors:** Sofia Geroyska, Isabel Mejia, Alfred A. Chan, Marian Navarrete, Vijaya Pandey, Samuel Kharpatin, Juliana Noguti, Feng Wang, Daniel Srole, Tsui-Fen Chou, James Wohlschlegel, Elizabeta Nemeth, Robert Damoiseaux, David B. Shackelford, Delphine J. Lee, Begoña Díaz

**Affiliations:** 1 The Lundquist Institute for Biomedical Innovation at Harbor-UCLA Medical Center, Torrance, California.; 2 Division of Hematology and Oncology at Harbor-UCLA Medical Center, David Geffen School of Medicine at UCLA, Los Angeles, California.; 3 Division of Dermatology at Harbor-UCLA Medical Center, David Geffen School of Medicine at UCLA, Los Angeles, California.; 4 Department of Biological Chemistry, David Geffen School of Medicine at UCLA, Los Angeles, California.; 5 Division of Pulmonary and Critical Care Medicine, David Geffen School of Medicine at UCLA, Los Angeles, California.; 6 Biology and Biological Engineering, California Institute of Technology, Pasadena, California.; 7 UCLA Center for Iron Disorders, Department of Medicine, David Geffen School of Medicine at UCLA, Los Angeles, California.; 8 Department of Molecular and Medical Pharmacology, David Geffen School of Medicine at UCLA, Los Angeles, California.; 9 California NanoSystems Institute at UCLA, Los Angeles, California.; 10 Department for Bioengineering, Samueli School of Engineering, UCLA, Los Angeles, California.; 11 Jonsson Comprehensive Cancer Center, UCLA, Los Angeles, California.

## Abstract

Myristoylation is a type of protein acylation by which the fatty acid myristate is added to the N-terminus of target proteins, a process mediated by N-myristoyltransferases (NMT). Myristoylation is emerging as a promising cancer therapeutic target; however, the molecular determinants of sensitivity to NMT inhibition or the mechanism by which it induces cancer cell death are not completely understood. We report that NMTs are a novel therapeutic target in lung carcinoma cells with LKB1 and/or KEAP1 mutations in a KRAS-mutant background. Inhibition of myristoylation decreases cell viability *in vitro* and tumor growth *in vivo*. Inhibition of myristoylation causes mitochondrial ferrous iron overload, oxidative stress, elevated protein poly (ADP)-ribosylation, and death by parthanatos. Furthermore, NMT inhibitors sensitized lung carcinoma cells to platinum-based chemotherapy. Unexpectedly, the mitochondrial transporter translocase of inner mitochondrial membrane 17 homolog A (TIM17A) is a critical target of myristoylation inhibitors in these cells. TIM17A silencing recapitulated the effects of NMT inhibition at inducing mitochondrial ferrous iron overload and parthanatos. Furthermore, sensitivity of lung carcinoma cells to myristoylation inhibition correlated with their dependency on TIM17A. This study reveals the unexpected connection between protein myristoylation, the mitochondrial import machinery, and iron homeostasis. It also uncovers myristoylation inhibitors as novel inducers of parthanatos in cancer, and the novel axis NMT-TIM17A as a potential therapeutic target in highly aggressive lung carcinomas.

**Significance:**

KRAS-mutant lung carcinomas with LKB1 and/or KEAP1 co-mutations have intrinsic therapeutic resistance. We show that these tumors are sensitive to NMT inhibitors, which slow tumor growth *in vivo* and sensitize cells to platinum-based chemotherapy *in vitro*. Inhibition of myristoylation causes death by parthanatos and thus has the potential to kill apoptosis and ferroptosis-resistant cancer cells. Our findings warrant investigation of NMT as a therapeutic target in highly aggressive lung carcinomas.

## Introduction

Myristoylation (protein lipidation with myristic acid; ref. 1) is mediated in human cells by two N-myristoyltransferases (NMT1 and NMT2; refs. 2, 3). This lipidation occurs at an N-terminal glycine within a myristoylation consensus motif, although proximal Lysine residues can also be myristoylated (4, 5). Myristoylation regulates protein function by increasing affinity for membranes, promoting calcium/myristoylation switches, facilitating palmitoylation (6), and preventing proteasomal degradation (7). NMT1 is overexpressed in various tumor types (8–12) and has been considered a cancer therapeutic target for years (13). However, few oncoproteins are myristoylated; thus, the mechanisms by which NMT1 contributes to cancer progression in most tumors are not well defined. The discovery of highly selective and potent inhibitors of NMTs such as DDD85646, DDD86481, and IMP-1088 (14, 15) has enabled mechanistic and preclinical studies in cancer (16–18). Notably, DDD86481 (PCLX-001) is currently in clinical trial for lymphoma and advanced solid malignancies (NCT04836195; ref. 19).

Lung carcinoma is the leading cause of death by cancer, with most cases corresponding to non–small cell lung carcinoma (NSCLC; ref. 20). NSCLCs with *KRAS* and *STK11* (encoding for LKB1) concurrent mutations have extensive metabolic reprogramming (21, 22) and are resistant to most treatments, including immunocheckpoint inhibitors (23). *KEAP1* mutations are commonly concurrent with *KRAS* and/or *STK11* mutations in NSCLC and confer tolerance to oxidative stress through the KEAP1/NRF2 pathway, promoting tumor progression and drug resistance (24–26). *KRAS*, *STK11*, and *KEAP1* triple mutant tumors exhibit extensive metabolic reprogramming (25), are highly resistant to chemotherapy, and have poor responses to immunocheckpoint inhibitors (27). Thus, novel therapeutics are urgently needed for this NSCLC subgroup.

Mitochondria are central to energy metabolism, redox signaling, and metabolic rewiring in tumors (28, 29). Notably, mitochondria activity and morphology are influenced by tumor driver mutations, histological subtype, and metabolic demands *in vivo* (30, 31). Most of the mitochondrial proteome is encoded by nuclear DNA, thus the machinery that imports and sorts proteins into mitochondria is key to mitochondria function and health (32). The translocase of the outer mitochondrial membrane 20 (TOM20) complex works along with the translocase of inner mitochondria membrane complexes 22 and 23 (TIM22 and TIM23) to transport proteins from the cytoplasm to the mitochondrial compartments (33). Translocase of inner mitochondrial membrane 17 homologs A and B (TIM17A and TIM17B) bind to the TIM23 subunit in the TIM23 complex to form heterodimers (TIM23-TIM17A or TIM23-TIM17B). TIM17A protein expression decreased after activation of the integrated stress response to adapt mitochondria protein import to stress, preventing mitochondria damage and facilitating cell survival (34). Conversely, elevated TIM17A transcript is associated with a worse prognosis in breast cancer (33). It remains unknown whether and how TIM17A contributes to cancer progression.

Mitochondria are also essential for the biogenesis of iron–sulfur cluster proteins and heme-containing proteins, which regulate DNA synthesis, repair, and protein translation and participate in the mitochondria electron transport chain (35–37). To fulfill these functions, mitochondria avidly take up and utilize iron (38). Because of its chemical properties, free iron (as opposed to protein-bound iron) generates reactive oxygen species (ROS; refs. 39, 40), which may induce oxidative stress if the cell’s antioxidant capacity is overcome. Sustained oxidative stress may cause cell death (41).

Parthanatos is one of the cell death mechanisms induced by oxidative stress (42). During parthanatos, excess poly-(ADP)-ribose (PAR) generated by hyperactivated PARP1 acts as a death signal that initiates the parthanatos cascade (42). Poly-ADP-ribosylation (PARylation) of apoptosis-inducing factor (AIF) causes its nuclear translocation (43, 44). PARylated AIF binds to macrophage migration inhibitory factor (MIF) before both translocate to the nucleus, where MIF cleaves DNA to finalize cell death (45). Parthanatos shares some features with apoptosis, including phosphatidylserine exposure to the cell surface, DNA fragmentation, and caspase activation (42, 43). Parthanatos, however, is independent of caspase activation and dependent on PARP activation; thus, it can be rescued with PARP inhibitors. Parthanatos has been mostly studied in the context of neurodegenerative diseases and stroke, in which it was first described (42, 46). However, recent findings indicate that cancer cells are also susceptible to parthanatos (47, 48), which represents a therapeutic opportunity to kill cancer cells that are resistant to other types of death.

## Material and Methods

### Cell lines

HeLa (cat. no. CCL-2 RRID:CVCL_0030) and lung carcinoma lines NCI-H460 (cat. no. HBT-177 RRID:CVCL_0459), NCI-H1792 (cat. no. CRL-5895 RRID:CVCL_1495), NCI-H522 (cat. no. CRL-5810 RRID:CVCL_1567), NCI-H1650 (cat. no. CRL-5883 RRID:CVCL_1483), and NCI-H1437 (cat. no. CRL-872 RRID:CVCL_1472) were obtained from ATCC and maintained at 37°C and 5% or 10% CO_2_ (for DMEM) in a humidified tissue culture incubator. HCC44 cells (cat. no. RRID:CVCL_2060) were from David Shackelford. HeLa cells were grown in DMEM (Corning cat. no. 10013CV) and lung carcinoma cells in RPMI-1640 (ThermoFisher Scientific cat. no. MT10040CV) both supplemented with 10% FBS (Fisher cat. no. MT35015CV). Authentication was performed with short tandem repeat analysis, and cells were regularly tested for mycoplasma infection using MycoAlert mycoplasm detection kit (Lonza cat. no. LT07218). Cells were maintained in culture for a maximum of 6 to 8 weeks before a new vial was thawed. Tet-system-approved FBS was from Clontech (cat. no. 631106).

### General reagents and inhibitors

NMT inhibitors (NMTi): DDD85646 was from Aobious (cat. no. AOB6657) or Cayman Chemical (cat. no. 13839), PCLX-001 was from Aobious (cat. no. AOB13563) or MCE (cat. no. HY-147308), and IMP-1088 was from Cayman Chemical (cat. no. 25366). Deferoxamine (cat. no. 14595), ferrostatin-1 (cat. no. 17729) liproxstatin-1 (cat. no. 17730), erastin (cat. no. 17754), trolox (cat. no. 10011659), necrostatin-1 (cat. no. 11658), ferroptosis suppressor protein 1 (FSP1) inhibitor (cat. no. 29483), olaparib (cat. no. 10621), Z-VA-DL-D(OMe)-FMK (cat. no. 27421), disulfiram (cat. no. 15303), deferoxamine (cat. no. 14597), staurosporine (cat. no. 81590), 4-hydroxynonenal (cat. no. 32100), cisplatin (cat. no. 13199), and pemetrexed (cat. no. 14269) were from Cayman Chemical. Ferric citrate (cat. no. F3388-250G) was from Sigma-Aldrich, crystal violet (cat. no. C581-25) was from Fisher, and propidium iodide (cat. no. 556463) was from BD Pharmingen. Hanks balanced salt solution (HBSS) was from ThermoScientific (cat. no. J67763.K2). Puromycin was from InVivoGen (cat. no. ANTPR1), and doxycycline was from MCE (cat. no. HY-N0565B).

### Primary antibodies

Antibodies against NMT1 (cat. no. 11546-1-AP, RRID:AB_2153157), TOM20 (cat. no. 11802-1-AP, RRID:AB_2207530), TOM40 (cat. no. 18409-1-AP, RRID:AB_2303725), SAM50 (cat. no. 28679-1-AP, RRID:AB_2881192), TIM17A (cat. no. 11189-1-AP, RRID:AB_2271661), TIM17B (cat. no. 11062-1-AP, RRID:AB_2201995), β-Tubulin (cat. no. 66240-1-Ig, RRID:AB_2881629), NDUFAF4 (cat. no. 26003-1-AP, RRID:AB_2880329), and FSP1/AIFM2 (cat. no. 20886-1-AP, RRID:AB_2878756) were from ProteinTech. Antibodies against poly/mono-ADP ribose (cat. no. 83732, RRID:AB_2749858), AIF (cat. no. 5318, RRID:AB_10634755), MIF (cat. no. 75038, RRID:AB_3101808), β-Actin (cat. no. 8457, RRID:AB_1095048), lamin A/C (cat. no. 4777, RRID:AB_10545756), phospho-H2A.X (cat. no. 9718, RRID:AB_2118009), and GAPDH (cat. no. 5174, RRID:AB_10622025) were from Cell Signaling Technology. HSP60 antibody (cat. no. MAB1800, RRID:AB_2118930) was from R&D Systems.

### RNA interference

siRNA against human NMT1 correspond to oligo 3026786108-000020 from Sigma; TIM17A SMARTpool siRNA (M-012739-02) was from Dharmacon. siRNA oligos #1 and #2 targeting human TIM17A correspond to oligo D-012739-02 (Dharmacon) and oligo 3031277378 (Sigma), respectively. NDUFAF4 SMARTpool siRNA (M-020684-01) and control non-targeting individual oligos or pools were from Dharmacon. siRNAs were transfected using Opti-MEM (Gibco 31985062) and Lipofectamine RNAimax (13778500, ThermoFisher Scientific), and cells were processed at 72 hours after transfection. pLKO-Tet-puro lentiviral constructs were used to clone the following shRNAs: NMT1 #10 (TRCN0000035710), NMT1 # 68 (TRCN0000289868), and TIM17A (TRCN0000275956). Constructs and lentivector stocks, including pLKO Tet-puro non-targeting control, were prepared by the Viral Vectors Shared Resource at Sanford-Burnham Prebys Medical Research Institute (La Jolla). Infected cells were selected with puromycin (2.5 μg/mL for H460, 1 μg/mL for H1792 and H1437, and 1.5 μg/mL for H522). To induce shRNA expression, doxycycline was used at 500 ng/mL for 72 hours.

### Western blotting

Proteins were extracted with RIPA lysis buffer containing fresh protease and phosphatase inhibitors. Cytoplasmic and nuclear fractions were separated with an NE-PER kit (ThermoFisher Scientific, cat. no. 78835). Total protein content was estimated using bicinchoninic acid assay (cat. no. 23225 Pierce). Western blotting was performed using standard protocols. Briefly, membranes were blocked for 1 hour at room temperature in 5% milk (Bio-Rad) and incubated overnight at 4°C. After washing in PBS containing 0.1% Tween 20, membranes were incubated with horseradish peroxidase-conjugated anti-mouse or anti-rabbit IgG (GE Healthcare). Images were acquired in a Bio-Rad ChemiDoc MP Imaging System using Supersignal WestPico PLUS (cat. no. 34580, Pierce). ImageJ software v1.52p (RRID:SCR_003070) was used for the quantification of digital images and Excel software for normalizing to protein loading control.

### Immunofluorescence

Cells were grown on glass coverslips and fixed in 4% paraformaldehyde (Electron Microscopy Sciences) for 15 minutes. For MIF staining, cells were subsequently fixed for 5 minutes in ice-cold methanol. Cells were blocked and permeabilized for 1 hour using PBS containing 0.1% Triton X100 and 3% Bovine Serum Albumin fraction V (BSA) from Fisher. Primary antibody was diluted in PBS containing 0.1% Triton X100 and 0.3% BSA and incubated overnight at 4°C. After washing, cells were incubated with anti-rabbit or anti-mouse IgG conjugated with AlexaFluor-488 or AlexaFluor-594 (ThermoFisher Scientific; 1:500) for 1 hour. Coverslips were mounted in Vectashield medium containing 4′,6-diamidino-2-phenylindole (DAPI; cat. no. H1200-10, Vector Labs). Immunofluorescence images were acquired using a fluorescence AxioImager ZEISS microscope provided with a ZEISS AxioCam 503 camera and ZEISS Zen Microscopy Software (RRID:SCR_013672). Images were exported as “TIF” and quantified using ImageJ 1.52p, or ZEISS Zen Microscopy Software. Adobe Photoshop (RRID:SCR_014199) was used to separate individual channels and/or crop digital images.

### Immunohistochemistry

Excised H460 tumors were fixed in formalin, dehydrated, and embedded in Paraplast Plus (Sigma, cat. no. P3683). Sections (0.3 μm) were prepared and processed for IHC using standard protocols. Briefly, sections were deparaffinized and rehydrated in distilled water. Heat-induced antigen retrieval was performed for 1 minute in a pressure cooker in a citrate-based antigen-unmasking solution (Vector, cat. no. H3300). After cooling down, sections were blocked for 10 minutes in Bloxall endogenous blocking solution (Vector, cat. no. SP-6000) and 20 minutes with normal goat serum (Vector, cat. no. S-1012). Primary antibodies against p-H2A.X and AIF were diluted in 1% BSA-containing TBS at 1:200 and incubated overnight at 4°C. After washing, samples were incubated with goat-anti-rabbit IgG biotinylated antibody (Vector, cat. no. BA-1000), Vectastain Elite ABC reagent (Vector, cat. no. PK-6100), and DAB (Vector, cat. no. SK-4100). Samples were dehydrated and mounted in Permount (Fisher, cat. no. SP-15). Images were taken on an ECHO Revolve microscope, exported as TIFF, and cropped using Adobe Photoshop.

### Iron measurement

#### a) Total iron content

Cells growing in 15-cm-diameter dishes treated with vehicle or NMTi were collected using 0.5% Trypsin EDTA-free (Hyclone, cat. no. SV3003701) and an aliquot reserved for cell counting. After low-speed centrifugation, cell pellets were weighted and immediately frozen. Inductively coupled plasma mass spectrometry (ICP-MS, NexION 2000, PerkinElmer) analysis was performed to detect iron in cell pellets. Each sample was transferred to clean teflon vessels for acid digestion. Digestion was carried out with concentrated HNO_3_ (65%–70%, Trace Metal Grade, Fisher Scientific) with a supplement of H_2_O_2_ (30%, Certified ACS, Fisher Scientific) at 190°C for 20 minutes in a microwave digestion system (Titan MPS, PerkinElmer). Once the sample was cooled to room temperature, it was subsequently diluted to make a final volume of 10 mL by adding filtered deionized water for analysis. The calibration curve was established using a standard solution while the dwell time was 50 milliseconds with 30 sweeps and three replicates with background correction. Iron content (ng/mg) was normalized to the total cell number for each sample and represented as iron content per million cells.

#### b) Ferric iron content

Cells treated with vehicle or NMTi were grown on glass coverslips and fixed in 10% neutral buffered formalin. Cells were subsequently washed with PBS and stained with Prussian blue (Polysciences, cat. no. 24199) following the vendor’s recommendation. Signal was amplified using Vector’s SG Substrate Kit (Vector Laboratories, cat. no. SK-4700) following the kit’s technical datasheet. Images were acquired using an Olympus IX83 microscope and bright field imaging. Iron deposits were quantified as percent area covered using ImageJ and data normalized to cell number.

### c) Cytoplasmic ferrous iron content

Cells growing on glass-bottom black 96-well plates (Agilent) were assayed for cytoplasmic ferrous iron content using FerroOrange (Dojindo, cat. no. F374) following the manufacturer’s protocol. Fluorescence at 560 nm was measured using a BioTek Synergy H1M plate reader. A duplicated 96-well plate containing cells treated in parallel was stained with 0.4% crystal violet in 30% Methanol for 30 minutes at room temperature. After washing, staining was extracted from dried plates with a 10% SDS solution, and absorbance was measured at 570 nm using a BioTek plate reader. Data from FerroOrange fluorescent intensity were normalized to crystal violet absorbance values for each experiment.

#### d) Mitochondrial ferrous iron content

Cells were grown on glass coverslips and treated with vehicle or NMTi for the indicated periods of time. Cells were washed twice with HBSS and treated with Mito-FerroGreen (Dojindo, cat. no. M489) working solution following the technical manual. Fluorescence images were taken randomly using a ZEISS AxioImager microscope provided with a ZEISS AxioCam 503 camera and ZEISS Zen Microscopy Software. Images were exported as TIFF files. ImageJ was used to quantify and determine the area covered by the green signal on each picture. After normalizing to the number of cells for each image in Excel, data were analyzed with GraphPad Prism 10 (RRID:SCR_002798).

### ROS measurement

Cells were grown on glass coverslips and treated with vehicle or NMTi for the indicated periods of time. After removing media, cells were washed twice with HBSS and treated with highly sensitive 2′−7′-dichlorodihydrofluorescein diacetate (DCFH-DA; Dojindo, cat. no. R252) at 1:2,000 in HBSS for 30 minutes following the technical manual. Fluorescence images were acquired randomly using a ZEISS AxioImager microscope provided with a ZEISS AxioCam 503 camera and ZEISS Zen Microscopy Software. Images were exported as “TIFF.” ImageJ was used to quantify the area covered by fluorescent signal on each picture. After normalizing to the number of cells for each image in Excel, data were analyzed with GraphPad Prism 10.

### Colony forming assay

Cells (∼500–1,000 per well) were plated in six-well plates and treated 24 hours after plating with the indicated amount of inhibitors or with doxycycline. After 6 to 10 days in culture, cells were stained with 0.1% crystal violet in 30% MeOH for 30 minutes, washed, and let dry. ImageJ was used to quantify colony number or % area covered on digital images.

### IC_50_ calculation

Cells were seeded in 384-well white plates (Greiner) at 375 to 1,500 cells per well according to the linear relationship measured from a standard curve of each cell line. 24 hours after seeding, cells were treated with NMTi (threefold dilution, eight dilution points, in duplicate). After 72 hours of treatment, cell viability was measured using CellTiter Glo Luminescent Cell Viability Assay (Promega), according to the manufacturer’s description, and IC_50_ values were calculated using the percentage of growth of treated cells versus the DMSO control with GraphPad Prism 10 software.

### CCK8 viability test

Cells growing in 96-well plates were treated with a vehicle or NMTi or co-treated with additional inhibitors for the indicated periods of time. Cell Counting Kit-8 (Dojindo cat. no. CK04) was incubated for 1 to 3 hours at 37°C, and absorbance was measured at 450 nm using a BioTek Synergy H1M. Values were analyzed with Excel and Prism 10.

### Cell death detection

#### a) Annexin/PI staining

Treated cells were analyzed using FITC Annexin V Apoptosis Detection Kit I, (BD cat. no. 556547). Briefly, after compound treatment, floating cells were collected and combined with adherent cells that were harvested using Accutase (BioLegend, cat. no. 423201). Cells were washed with cold PBS and resuspended at 1 × 10^6^ cells/mL in the provided binding buffer. Cells (1 × 10^5^ total) were incubated for 15 minutes in the dark with 1 μL of FITC Annexin V and 5 μL propidium iodide (PI). After staining, 400 μL of binding buffer was added to each tube prior to analysis using a BD LSRII cell analyzer (BD Biosciences). Single stains for Annexin V and PI of untreated cells were used for compensation. The distribution of cell populations was performed using FlowJo (RRID:SCR_008520) 10.6.2 software (BD Biosciences).

#### b) Propidium iodide staining

Floating cells were collected and combined with adherent cells that were harvested using TrypLE Express Enzyme reagent (Gibco, cat. no. 126050). Cells were washed with PBS and resuspended in 1 mL of PBS-EDTA at a cell concentration of 1 × 10^6^ cells/mL. PI (5 μL) was added to each sample and incubated for 15 minutes at room temperature in the dark. Samples were analyzed using a BD LSRII cell analyzer (BD Biosciences). Samples were compensated to unstained cells treated as above. The distribution of dead and alive cell populations was calculated using FlowJo 10.6.2 software (BD Biosciences).

#### c) Dead/live imaging kit

Cultured cells were washed with HBSS and stained with live/dead cell imaging kit (ThermoFisher, cat. no. R37601) dissolved in HBSS following the technical manual. An ECHO Revolve microscope was used to acquire images. ImageJ was used to manually score the number of green and red cells on each image. Data were analyzed in Excel and GraphPad Prism 10.

### Cell cycle analysis

After compound treatment, floating cells were collected and combined with adherent cells that were harvested with Accutase (BioLegend, cat. no. 423201). Cells were fixed in 70% ethanol overnight at −20°C. Fixed cells were washed two times with PBS and pellets resuspended in 0.5 mL of PI staining buffer (BD Biosciences, cat. no. 550825) at a concentration of 2 × 10^6^ cells/mL and incubated at room temperature for 30 minutes in the dark prior analysis. Fluorescence intensity was measured using a BD LSRII cell analyzer (BD Biosciences). For each sample, at least 1 × 10^5^ events were recorded. Collected events were analyzed using FlowJo 10.6.2 software (BD Biosciences) to determine cell cycle distribution.

### Lipid peroxidation detection

Treated cells were incubated with 5 μmol/L of Bodipy 581/591 C11 (ThermoFisher, cat. no. D3861) in complete medium for 30 minutes at 37°C. Cells were collected using TrypLE Express Enzyme reagent (Gibco cat. no. 126050), washed, and resuspended in ice-cold PBS. Data acquisition and analysis were performed in a BD FACSyphony A5 flow cytometer (BD Biosciences) using BD FACSDiva Software (RRID:SCR_001456) v9.0. The median fluorescence intensity for the green channel of each sample was determined and normalized to DMSO-treated control cells using FlowJo v10.8.1 software (BD Biosciences).

### Electron microscopy

Cells growing in tissue culture plates were fixed with 2.5% glutaraldehyde in 0.1-mol/L sodium cacodylate buffer pH 7.4 for 1 hour on ice. Cells were scraped and pelleted. After washing with 0.1-mol/L cacodylate buffer, pellets were postfixed in 1% OsO4 in 0.1-mol/L cacodylate buffer for 1 hour on ice, stained with 2% uranyl acetate for 1 hour on ice, and dehydrated in graded series of ethanol (50%–100%) while remaining on ice. The cells were then subjected to one wash with 100% ethanol and two washes with acetone (10 minutes each) and embedded with Durcupan. Sections were cut at 60 nm on a Leica UCT ultramicrotome and picked up on 300-mesh copper grids. Sections were post-stained with 2% uranyl acetate for 5 minutes and Sato’s lead stain for 1 minute. The images were acquired with a Tecnai G2 Spirit BioTWIN operated at 80 KeV and equipped with an FEI eagle 4k × 4k camera.

### Xenograft mouse model

All animal work was performed in strict accordance with an animal usage protocol approved by the University of California Los Angeles Animal Care and Use Committee (IACUC# ARC-2012-094). NSG female mice RRID:IMSR_JAX:005557 (Jackson Laboratories) of ∼8 weeks of age were injected subcutaneously in one flank with a suspension of saline and ∼30% Matrigel (Corning cat. no. 356231) containing 1 × 10^6^ H460 cells. Around 2 weeks after injection, animals were distributed into three experimental groups with an average tumor volume of ∼140 mm^3^ each and dosed with vehicle (12.5% DMSO in saline) or PCLX-001 via daily subcutaneous injection in the scruff of the neck (we found that intraperitoneal injection was not tolerated). Control group contained nine animals, and each of the experimental groups contained 10. Consistent with a previous report (18), mice receiving PCLX-001 suffered dehydration and weight loss. Body weight was monitored daily and treatment of mice in the 50 mg/kg group was discontinued after 10 days due to sustained weight loss. From day 10 to endpoint (day 18) animals in the 50 mg/kg group were only injected subcutaneously with 200 μL of lactated Ringer’s solution. Tumor volume was estimated using the formula *V* = 0.5 × *L* × *W*^*2*^, where *L* is the tumor length (highest dimension) and *W* is the tumor width.

### Mass spectrometry–based proteomics

H1792 cells treated with DMSO control or 1-μmol/L DDD85646 for 48 hours (*n* = 3 biological replicates) were lysed in 8-mol/L urea, 100-mmol/L Tris-Cl, and pH 8.0. An equal amount of protein (estimated using bicinchoninic acid assay cat. no. 23225 from Pierce) for each sample was reduced and alkylated by the sequential addition of 5-mmol/L tris (2-carboxyethyl) phosphine and 10-mmol/L iodoacetamide. This was followed by treatment with a single-pot, solid-phase-enhanced sample preparation (SP3) protocol for protein clean-up (49). Following SP3, eluates were proteolytically digested with Lys-C and trypsin at 37°C overnight. Peptides were subjected to offline SP3-based peptide clean-up and subsequently analyzed by LC-MS/MS. Briefly, peptides were separated by reversed-phase chromatography using a 75-μm-inner-diameter fritted fused silica capillary column packed in-house to a length of 25 cm with bulk 1.9-mmol/L ReproSil-Pur beads with 120-Å pores. The increasing gradient of acetonitrile was delivered by a Dionex Ultimate 3000 (ThermoFisher Scientific) at a flow rate of 200 nL/minute. The MS/MS spectra were collected using data-dependent acquisition on Orbitrap Fusion Lumos Tribrid mass spectrometer (ThermoFisher Scientific) with an MS1 resolution (r) of 120,000 followed by sequential MS2 scans at a resolution (r) of 15,000. The data generated by LC-MS/MS were analyzed on MaxQuant (RRID:SCR_014485) bioinformatic pipeline (50). The Andromeda integrated into MaxQuant was employed as the peptide search engine, and the data were searched against *Homo sapiens* (Uniprot Reference UP000005640). Briefly, a maximum of two missed cleavages was allowed. The maximum FDR for peptide and protein was specified as 0.01. Label-free quantification was enabled with a label-free quantification minimum ratio count of 1. The parent and peptide ion search tolerances were set as 20 and 4.5 ppm, respectively. The MaxQuant output files were subsequently processed for statistical analysis of differentially enriched proteins using Analytical R Tools for Mass Spectrometry (51). Briefly, the MSstats function of this package was used for relative quantification and global median normalization of protein intensities. Prior to statistical analysis, missing protein abundance values were imputed based on the lowest value of protein abundance detected in that sample under the assumption that failure to detect a protein in a sample was due to its low abundance. Log_2_ fold changes were calculated for indicated comparisons. Significance testing for differential expression was determined using a moderated *t* test from the LIMMA (RRID:SCR_010943) package and FDR adjustment by the Benjamini–Hochberg method with a probability of a false-positive discovery cutoff of 0.05.

### Data mining

Publicly available gene expression data from The Cancer Genome Atlas (TCGA) lung adenocarcinoma (LUAD) was queried from BioPortal (RRID:SCR_002713; ref. 52) Specifically TCGA-LUAD Pan Cancer Atlas, *n* = 501, *n* = event = 181 was used. Patient information such as age and overall survival status and months were also provided. For gene expression values, the RSEM (RNA seq by expectation-maximization) batch normalized count data were used (from Illumina HiSeq_RNASeqV2). Differential gene expression analysis for TCGA data was also performed using BioPortal.

The lung carcinoma IC_50_ (half-maximal inhibitory concentration) for the NMTi ICL1100013 (DDD85646) was queried from *Genomics of Drug Sensitivity in Cancer* (RRID:SCR_011956) v8.4 July 2022. *KRAS*, *EGFR*, *TP53*, *STK11*, and *KEAP1* mutation status for LUAD cell lines were queried from the *Cancer Dependency Map* portal (RRID:SCR_017655). Comparison among the three groups *KRAS*, *STK11*, and *KEAP1* log_2_ transformed was performed by ANOVA assuming equal variance.

Pathway analysis by gene over-representation was performed using Fisher’s exact test against the WikiPathways (RRID:SCR_002134) database as well as Gene Ontology (RRID:SCR_002811) biological processes and cellular components. A list of upregulated and downregulated genes was inputted separately for over-representation analysis on WebGestalt: WEB-based GEne SeT AnaLysis Toolkit (RRID:SCR_006786). Benjamini–Hochberg procedure was applied to correct the FDR associated with multiple hypothesis testing.

The subset of proteins specific to the mitochondria was queried using the Human Protein Atlas HPA (RRID:SCR_006710). According to this database, 6% (1,119 proteins) of all human proteins have been experimentally detected in the mitochondria of which 539 were detected in our dataset. Moderated *t*-statistics from the entire proteomic data array was used.

TIM17A dependency in LUAD cell lines was queried from the *Cancer Dependency Map* portal (DepMap-23Q2). TIM17A dependency was estimated using the CRISPR DepMap Scores with the Chronos algorithm. Association between NMTi sensitivity (IC_50_) and TIM17A dependency (CRISPR DepMap Scores) was tested using Spearman’s rank correlation.

### Statistical analysis

Comparison of survival estimates between the two groups was performed using a log-rank test and plotted using Kaplan–Meier curves with shaded bands for 95% confidence intervals. Overall survival was defined as the interval from initial diagnosis to patient death or last follow-up (censored at 8 years).

Comparison of means between the two groups was performed using a two-sided Student *t* test with an alpha threshold of 0.05 for statistical significance. Excel, GraphPad Prism 10, and R Project for Statistical Computing (RRID:SCR_001905) software were used for statistical analysis and plotting graphs. For testing whether two continuous variables are significantly correlated, the non-parametric Spearman’s rank test was used.

### Additional software

Diagrams in Figs. 4, 5, and 8 were created with Biorender (RRID:SCR_018361).

### Data availability

Materials and data generated during the current study are available from the corresponding author upon reasonable request.

## Results

### N-myristoyltransferase-1 is a novel therapeutic target in aggressive and therapy-resistant lung carcinoma

Pharmacological inhibition of NMTs is emerging as a promising therapeutic strategy in lymphoma (18, 19), but its therapeutic potential in lung carcinoma, the cancer causing the highest number of annual deaths, has not been fully explored. We used TCGA to explore the relationship between NMT transcript expression and patient outcome and found that high NMT1 (but not NMT2) transcript correlated with poor outcomes in LUAD (Supplementary Fig. S1A).

**Figure 1:**
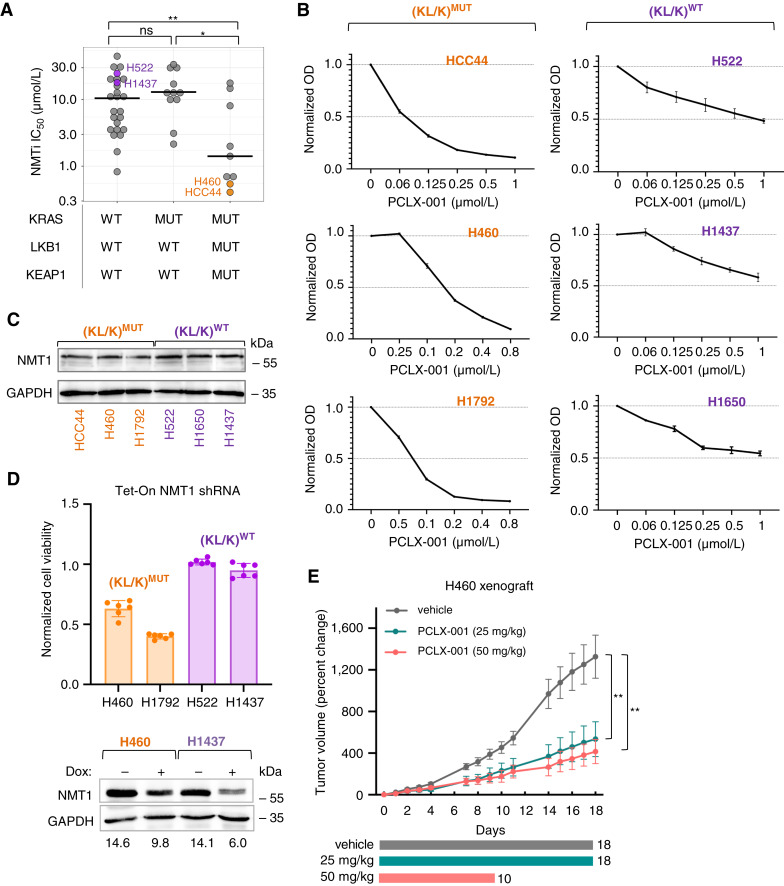
Lung carcinoma cells with LKB1 and/or KEAP1 mutations in a KRAS-mutant background are sensitive to myristoylation inhibition. **A,** NMTi IC_50_ for lung carcinoma cells with the indicated mutational status were compared. IC_50_ values for ICL1100013 (DDD85646) were from *Genomics of Drug Sensitivity in Cancer* and mutational profiles from *DepMap*. **, *P* = 0.008; *, *P* = 0.005; ns = not significant (Student *t* test). Crossbar, median. **B,** Viability test (CCK8) at 72 hours of PCLX-001 treatment. Optical density (OD) values were normalized to vehicle-treated samples. Error bars, SEM. **C,** NMT1 immunoblotting on the indicated cells. GAPDH, loading control. **D,** Relative viability (CCK8) of doxycycline (Dox) treated vs. untreated Tet-inducible NMT1#10 shRNA cells. Blot: NMT1 immunoblotting in cells with or without Dox. GAPDH, loading control. **E,** Percent change in H460 (KL/K)^MUT^ xenograft tumor volume from mice treated with daily subcutaneous injections of vehicle control or PCLX-001 at dosages of 25 and 50 mg/kg. Treatment was indicated by the colored bars (18 days for the 25 mg/kg group and 10 days for the 50 mg/kg group). **, *P* = 0.008, one-way ANOVA, Tukey’s multiple comparisons test.

We previously showed that H460 and H1792 KRAS mutant lung carcinoma cells are sensitive to the NMTi DDD85646 (16). We calculated the half inhibitory concentration (IC_50_) of DDD85646 on EGFR mutant (H1975 and H1650) and KRAS/EGFR wild-type lung carcinoma cells (H1299 and H522) at 72 hours of treatment. This revealed heterogeneous responses to NMTi treatment (Supplementary Fig. S1B). To expand this analysis, we used *Genomics of Drug Sensitivity in Cancer*, where DDD85646 is deposited as ICL1100013 (bioRxiv 2021.03.20.436222). A total of 58 NSCLC lines were manually annotated for their driver mutation status based on the *Cancer Dependency Map* portal. Our search for associations between NMTi sensitivity and driver mutations revealed that cells with a triple mutation in KRAS, LKB1, and KEAP1 (*n* = 9) were more sensitive (*P* = 0.008) to NMT inhibition than those lacking any of these mutations (*n* = 25) or than those containing KRAS mutation alone (*n* = 11, *P* = 0.005; Fig. 1A). The number of double mutant KRAS/LKB1 or KRAS/KEAP1 was not sufficiently large to perform statistical analysis. Our data suggest that NMT1 is a potential therapeutic target in lung carcinomas with mutant KRAS and LKB1 and/or KEAP1 (KL/K)^MUT^, which are highly aggressive and resistant to current therapies, including immune checkpoint inhibitors (27).

We selected three (KL/K)^MUT^ and three (KL/K)^WT^ lung carcinoma cell lines for further study: The first group included HCC44 and H460 (KRAS/LKB1/KEAP1 triple mutant) along with H1792 (KRAS/KEAP1 double mutant). The second group included H522 and H1650 (KRAS/LKB1/KEAP1 wild-type), along with H1437 (KRAS and KEAP1 wild-type and LKB1 mutant). In addition to DDD85646, other NMTis have been characterized for their sensitivity and specificity, including the derivative PCLX-001 (18) and the structurally unrelated IMP-1088 (Supplementary Fig. S1C; ref. 15). The viability of the selected cell lines after a dose–response of PCLX-001 for 72 hours (Fig. 1B) was comparable with the sensitivity to DDD85646 deposited in *Genomics of Drug Sensitivity in Cancer.* (KL/K)^MUT^ cells were sensitive (EC_50_ below 0.2 μmol/L), whereas (KL/K)^WT^ cells were relatively resistant (EC_50_ values over 1 μmol/L; Fig. 1B). The responses to DDD85646 and IMP-1088 were comparable with PCLX-001 (Supplementary Fig. S1D and S1E). NMT1 immunoblotting, however, revealed no differences in NMT1 protein expression between the six cell lines (Fig. 1C).

To demonstrate that the effects of NMTi on cell viability were on target, we generated H460, H1792, H522, and H1437 cells stably expressing a Tet-inducible shRNA targeting NMT1 and use them to compare cell viability upon doxycycline treatment for 72 hours. Doxycycline did not affect the viability of cells expressing non-targeting shRNA control (Supplementary Fig. S1F). Although the viability of H460 and H1792 (KL/K)^MUT^ decreased by 60% and 40%, respectively, the viability of H522 and H1437 (KL/K)^WT^ was not altered by doxycycline treatment despite a comparable level of NMT1 knockdown (Fig. 1D).

Using Tet-inducible H460 cells expressing two different NMT1 shRNAs, we confirmed that colony formation ability was dependent on the degree of NMT1 silencing (Supplementary Fig. S1G). H1792 and HCC44 cell lines expressing inducible NMT1 shRNA also showed decreased colony-forming ability upon doxycycline treatment (Supplementary Fig. S2A and S2B).

Notably, the NMTi PCLX-001 efficiently reduced tumor growth in H460 xenografts at dosages of 25 and 50 mg/kg daily (Fig. 1E; Supplementary Fig. S2C). Animals in the 50 mg/kg group experienced dehydration and weight loss (Supplementary Fig. S2D) and thus were only treated for 10 days. Despite treatment discontinuation, the anti-tumor effect of PCLX-001 was maintained until the end of the study (Fig. 1D). Taken together, our data indicate that NMT1 is a novel therapeutic target in (KL/K)^MUT^ lung carcinoma, an aggressive and therapy-resistant lung cancer subtype.

### N-myristoyltransferase inhibition alters transferrin receptor trafficking and decreases cytoplasmic ferrous iron content

KRAS is not myristoylated and NMT1 protein expression levels were comparable between sensitive and resistant cells (Fig. 1C). Thus, the molecular determinants of the sensitivity of (KL/K)^MUT^ cells to NMTi treatment were unclear.

**Figure 2:**
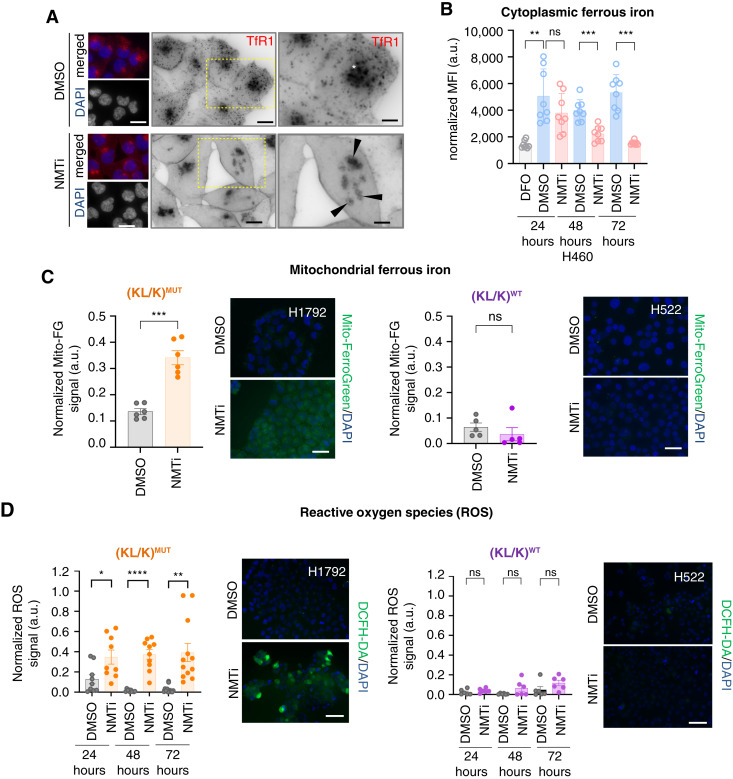
NMT inhibition causes mitochondrial ferrous iron accumulation and increases ROS in (KL/K)^MUT^ but not (KL/K)^WT^ lung carcinoma cells. **A,** TfR1 and DAPI staining on (KL/K)^MUT^ H460 cells treated with 0.5 μmol/L DDD85646 (NMTi) or vehicle for 72 hours. Images were inverted for clarity. Right column, amplification of the area in the square in the middle column. Bar, 15 μm in left and middle columns, 5 μm in right column. (*), endocytic recycling compartment. Arrowheads, intracellular TfR1 clusters. **B,** Cytoplasmic ferrous iron measured using FerroOrange in H460 cells (KL/K)^MUT^ treated with 0.5 μmol/L DDD85646 (NMTi) or vehicle for the indicated times. Two independent experiments with four technical replicates each were combined. Deferoxamine (DFO, 7 μmol/L) was used as negative control. Fluorescence was normalized to cell number and expressed as arbitrary units (a.u.). Bar, group mean; error bars, SD. **, *P* = 0.0014; ***, *P* = 0.0002; ns = not significant (Student *t* test). **C,** Mitochondrial ferrous iron (Mito-FerroGreen) measured in (KL/K)^MUT^ and (KL/K)^WT^ lung carcinoma cells treated with 1 μmol/L DDD85646 or vehicle for 24 hours. Signal intensity was quantified in randomly imaged fields containing at least 200 cells per condition. Bar, group mean; error bars, SEM; a.u., arbitrary units. ***, *P* = 0.0002; ns, not significant (Student *t* test). Representative images are shown. Bar, 15 μm. **D,** ROS detected using DCFH-DA in (KL/K)^MUT^ and (KL/K)^WT^ lung carcinoma cells treated with 1 μmol/L DDD85646 for the indicated times. Signal intensity was quantified in randomly imaged fields containing at least 720 cells per condition. Bars, mean; error bars, SEM; a.u., arbitrary units. *, *P* = 0.0202; ****; *P* < 0.0001, **; *P* = 0.0017; ns, not significant (Student *t* test). Representative images from 72 hours of treatment are shown. Bar, 30 μm.

We performed mass spectrometry–based proteomics analysis (LC-MS/MS) on (KL/K)^MUT^ H1792 lung carcinoma cells treated with 1 μmol/L DDD85646 or vehicle control for 48 hours, when cells were still viable (Supplementary Table S1; ref. 16). Over-representation analysis of proteins upregulated with NMTi treatment (765/4,929 uniprot IDs, *P* < 0.05) by Fisher’s exact test (*P* adjusted < 0.05) against Gene Ontology biological processes revealed enrichment of secretory and vesicle transport-related processes in drug-treated cells (Supplementary Fig. S3A), a finding consistent with a previous study on HeLa cells (17). Using transferrin receptor 1 (TfR1) staining as a surrogate for vesicle trafficking within the endocytic recycling pathway, we found that NMTi caused accumulation of TfR1 at the plasma membrane and intracellular clusters, but staining was absent from intracytoplasmic vesicles (Fig. 2A). This effect was not a consequence of cell death because the normal distribution of TfR1 was almost recovered ∼24 hours after drug removal even in cells treated for 72 hours (Supplementary Fig. S3B).

TfR1 is the main mechanism for cellular uptake of iron, an essential metal that is necessary for DNA replication (53). Disrupted TfR1 trafficking could decrease cellular iron availability, halt DNA replication, and decrease cell viability. However, ferric citrate (a TfR1-independent cell-permeable form of iron) did not increase the colony-forming ability of cells treated with DDD85646 (Supplementary Fig. S3C), indicating that NMT inhibition is unlikely to cause global iron deficiency. In agreement, measurement of total iron content using ICP-MS in H460 cells showed no differences between control and NMTi treatment at 24, 48, or 72 hours (Supplementary Fig. S3D). This was largely consistent with our analysis of ferric iron content using enhanced Perls’ Prussian Blue staining in H1792 cells treated with DDD85646 (Supplementary Fig. S3E). We concluded that NMTi treatment does not decrease total iron availability in NMTi-sensitive lung carcinoma cells despite altered TfR1 subcellular distribution.

The cellular levels of iron are strictly regulated to avoid deficiency while preventing toxicity resulting from the iron-dependent generation of ROS. Accumulation of TfR1 at the plasma membrane (Fig. 2A; Supplementary Fig. S3B) is a marker of ferroptosis (54), a ferrous iron-mediated cell death mechanism characterized by excessive lipid peroxidation. To investigate whether NMTi treatment could cause ferroptosis, we first measured cytoplasmic ferrous iron content using FerroOrange. Surprisingly, cytoplasmic ferrous iron levels were decreased at 48 and 72 hours of treatment with NMTi in H460 and H1792 cells (Fig. 2B; Supplementary Fig. S4A). Taken together, our data indicate that NMT inhibition alters ferrous iron homeostasis without decreasing total cellular iron content.

### N-myristoyltransferase inhibition causes mitochondrial ferrous iron overload, elevated ROS, and excessive lipid peroxidation in sensitive lung carcinoma cells

We reasoned that decreased cytoplasmic ferrous iron content in drug-treated cells could occur at the expense of ferrous iron accumulation in a different subcellular compartment. Using Mito-FerroGreen, we observed that NMTi caused mitochondrial ferrous iron accumulation at 24 hours of treatment in sensitive (KL/K)^MUT^ cells (Fig. 2C; Supplementary Fig. S4B). In contrast, none or little ferrous iron accumulated in the mitochondria of resistant (KL/K)^WT^ cells (Fig. 2D; Supplementary Fig. S4B). Transfection of NMT1 siRNA into HeLa cells [which are sensitive to NMTi (16, 17)] also led to mitochondrial ferrous iron overload (Supplementary Fig. S4C).

Because free ferrous iron is a known source of ROS, we evaluated ROS generation after NMTi treatment using DCFH-DA. As expected, NMTi treatment of (KL/K)^MUT^ cells led to a sustained increase in ROS from 24 to 72 hours of treatment, whereas a modest or no increase was seen in (KL/K)^WT^ cells (Fig 2D; Supplementary Fig. S4D). Excessive ROS, which crosses cellular membranes causes widespread lipid peroxidation, a known consequence of oxidative stress that is associated with ferroptosis. Using the lipid peroxidation sensor Bodipy 581/591 C11 and flow cytometry we confirmed that NMTi increased lipid peroxidation in a dose- and time-dependent manner in (KL/K)^MUT^ H460 cells (Supplementary Fig. S5A and S5B). The NMTis PCLX-001 and IMP-1088 had a similar effect (Supplementary Fig. S5C).

**Figure 3:**
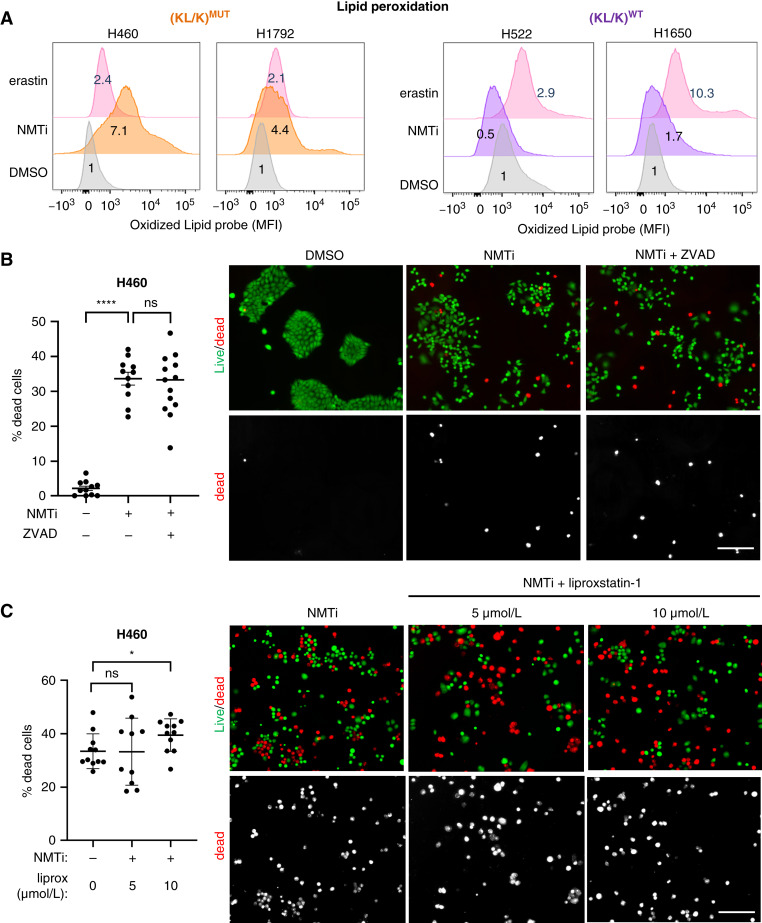
NMT inhibition increases lipid peroxidation and induces caspase-independent cell death in (KL/K)^MUT^ lung carcinoma cells. **A,** Lipid peroxidation measured using Bodipy 581/591 C11 and flow cytometry in (KL/K)^MUT^ and (KL/K)^WT^ lung carcinoma cells treated with 1 μmol/L DDD85646 (NMTi) or vehicle control (DMSO) for 96 hours. Erastin (10 μmol/L for 24 hours) was used as positive control. Green fluorescence (oxidized probe) was normalized to control DMSO. *n* = 2 independent experiments. MFI, mean fluorescence intensity. **B,** Cell viability (Live/dead reagent) was analyzed in (KL/K)^MUT^ cells treated for 72 hours with vehicle control or 1 μmol/L DDD85646 (NMTi) in the presence or absence of 25-μmol/L ZVAD-FMK added freshly every 24 hours. Graph: percentage of dead cells calculated from at least 5,000 cells per condition. Bar, group mean, error bars, SEM. ****, *P* < 0.0001; ns, not significant (Student *t* test). Representative images are shown. Bar, 100 μm. **C,** Cell viability (live/dead reagent) analyzed in (KL/K)^MUT^ cells treated for 72 hours with vehicle control or 1 μmol/L DDD85646 (NMTi) in the presence or absence of the ferroptosis inhibitor liproxstatin (5 and 10 μmol/L). Graph: percentage of dead cells calculated from at least 5,500 cells per condition. Crossbar, group mean; error bars, SEM. *, *P* < 0.05; ns, not significant (Student *t* test). Representative images are shown. Bar, 100 μm.

Next, we wonder whether the extent of lipid peroxidation correlated with sensitivity to NMTi. DDD85646 treatment (2 μmol/L for 96 hours) led to increased lipid peroxidation in NMTi-sensitive but not in NMTi-resistant cells (Fig. 3A). Thus, lipid peroxidation in sensitive cells was likely caused by excessive ROS generated due to mitochondrial ferrous iron accumulation. Consistent with that possibility, both the iron chelator deferoxamine and the antioxidant trolox attenuated lipid peroxidation in sensitive H460 cells treated with DDD85646 (Supplementary Fig. S5D and S5E). NMTi and genetic targeting of NMT1 also increased lipid peroxidation in HeLa cells (Supplementary Fig. S5F and S5G).

FSP1 (ferroptosis suppressor protein 1) is a myristoylated protein that protects from lipid peroxidation and ferroptosis (55, 56). Our proteomic analysis revealed decreased abundance of FSP1 after NMTi treatment, which we verified by immunoblotting (Supplementary Fig. S5H). However, although inhibition of FSP1 with the small compound FSP1 inhibitor (55) sensitized H460 cells to NMTi, it did not cause significance cell death as a single agent (Supplementary Fig. S5I). Thus, loss of FSP1 is unlikely to explain the sensitivity of lung carcinoma cells to NMTi treatment.

Taken together, our findings indicate that NMT inhibition in sensitive (KL/K)^MUT^ lung carcinoma cells causes mitochondrial ferrous iron overload, which leads to excessive ROS generation and lipid peroxidation.

### Inhibition of N-myristoyltransferase causes parthanatos in (KL/K)^MUT^ lung carcinoma cells

We previously showed that NMTi decreases cell proliferation in sensitive H1792 and H460 cells (16). Using flow cytometry and cell cycle analysis, we now confirmed that NMTi prevents cell cycle progression, leading to the accumulation of cells in G1 (Supplementary Fig. S6A). We also reported that H1792 cells die after ∼72 hours of NMTi treatment (16). To investigate the mechanism by which sensitive lung cancer cells die in response to NMTi, we performed Annexin-V/PI staining of H460 cells treated with 2 μmol/L of DDD85646 for 96 hours (Supplementary Fig. S6B). NMTi increased the amount of Annexin V and Annexin-V/PI double-positive cells (considered as early and late apoptotic respectively). There was also a modest increase in cells with high PI and low Annexin-V staining (considered as non-apoptotic death). Although this analysis indicated death by apoptosis, co-treatment of H460 cells with the pan-caspase inhibitor Z-VAD-FMK failed to prevent death induced by NMTi (Fig. 3B) despite preventing apoptosis of H460 cells treated with 0.2-μmol/L staurosporine for 24 hours (Supplementary Fig. S6C). The above finding indicates that sensitive lung carcinoma cells die by a caspase-independent mechanism in response to NMTi. Despite the phenotypic similarities of NMTi-induced death with caspase-independent types of death such as ferroptosis (accumulation of TfR1 at the plasma membrane and excessive lipid peroxidation) or pyroptosis (excessive lipid peroxidation), death of H460 cells treated with DDD85646 was not rescued by inhibitors of ferroptosis (liproxstatin-1, Fig. 3C; Supplementary Fig. S7A) or pyroptosis (disulfiram, Supplementary Fig. S7B). Inhibitors of necroptosis (necrostatin-1) also failed to rescue lung carcinoma cells from NMTi-induced cell death (Supplementary Fig. S7C).

**Figure 4:**
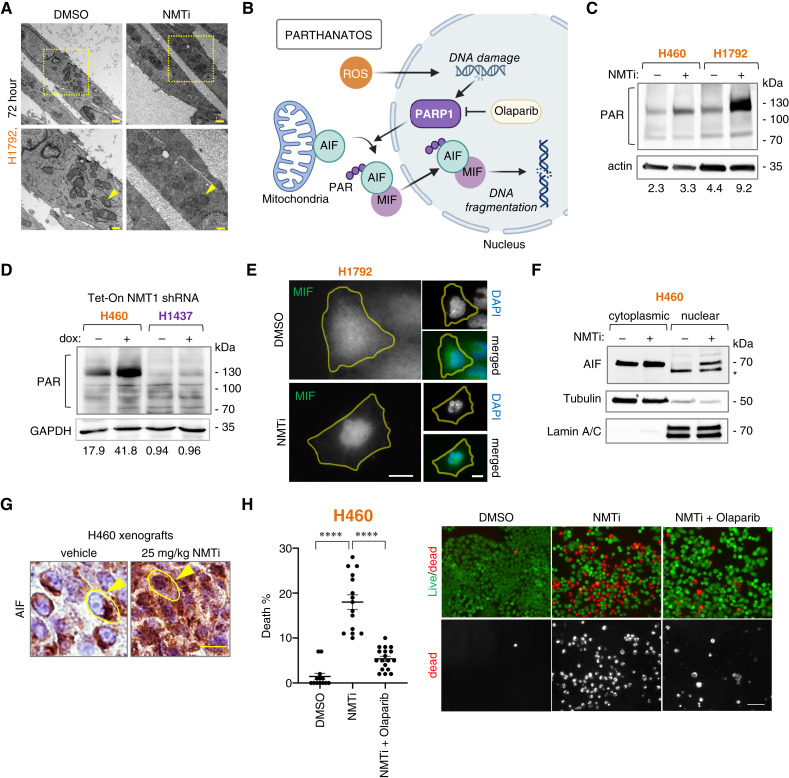
NMTi treatment induces parthanatos in (KL/K)^MUT^ lung carcinoma cells. **A,** Ultrastructure of (KL/K)^MUT^ cells treated with 1 μmol/L DDD85646 (NMTi) or vehicle control for 72 hours. Bottom, Magnification of areas in the squares. Arrowheads, mitochondria. Bars, 1 μm (top) and 0.5 μm (bottom). **B,** Diagram of the parthanatos components. **C,** Detection of PARylation (PAR) by immunoblotting in lysates from (KL/K)^MUT^ cells treated with 1 μmol/L PCLX-001 (NMTi) for 72 hours. Actin, loading control. **D,** Detection of PARylation (PAR) by immunoblotting in lysates from (KL/K)^MUT^ H460 and (KL/K)^WT^ H1437 cells expressing Tet-inducible NMT1 shRNA treated with or without Dox. GAPDH, loading control. Note that GAPDH is identical to that in Fig. 1E because the same membrane was used to stain NMT1 and PAR. **E,** MIF subcellular localization in (KL/K)^MUT^ H1792 cells treated with vehicle control of 1 μmol/L PCLX-001 (NMTi) for 96 hours and processed for immunofluorescence using a MIF antibody. Representative images are shown. Bar, 5 μm in left column, 15 μm in right column. **F,** AIF1 immunoblotting in cytoplasmic and nuclear lysates of (KL/K)^MUT^ H460 treated with 1 μmol/L PCLX-001 (NMTi) o vehicle for 72 hours. Tubulin and lamin staining were used to verify fraction purity. (*) non-specific band. **G,** AIF immunofluorescence in tumor sections from (KL/K)^MUT^ H460 xenografts from animals treated with 25 mg/kg PCLX-001 (NMTi) or vehicle control. Arrowheads, nuclear AIF. Bar, 10 μm. **H,** Cell viability (live/dead reagent) in (KL/K)^MUT^ H460 cells treated with 1 μmol/L PCLX-001 (NMTi) or vehicle control and 20 μmol/L olaparib for 72 hours. Representative fluorescent images are shown. Bar, 30 μm. Graph, percentage of dead cells calculated from at least 5,000 cells per condition. Crossbar, group mean; error bars, SEM. ****, *P* < 0.0001 (Student *t* test).

To gain insights into the elusive mechanism by which (KL/K)^MUT^ lung carcinoma cells die in response to NMTi, we used transmission electron microscopy to analyze the ultrastructure of H1792 cells treated with 1 μmol/L DDD85646 for 24 and 72 hours. Notably, cells treated for 72 hours showed a striking electron-dense cytoplasm previously described in dark microglia cells as the result of extensive oxidative damage (Fig. 4A; ref. 57). Parthanatos is a type of apoptosis-independent programmed cell death characterized by extensive oxidative stress (42). During parthanatos, PARP is hyperactivated and causes extensive poly-ADP-ribosylation (PARylation) of proteins, including AIF, which then translocates from mitochondria to the nuclei along with MIF (58), causing fragmentation of DNA into large fragments, and cell death (see diagram in Fig. 4B). We had noticed that the nuclei of dying cells contained large DNA fragments (Supplementary Fig. S8A); thus, we investigated whether NMTi causes parthanatos in (KL/K)^MUT^ lung carcinoma cells.

Immunoblotting with an anti-PAR antibody revealed increased PARylated proteins in NMTi-treated lung carcinoma cells (Fig. 4C). In addition, sensitive H460 but not resistant H522 cells expressing a Tet-inducible NMT1 shRNA had increased PARylation in the presence of doxycycline (Fig. 4D). Immunofluorescence revealed the accumulation of nuclear MIF in H460 and H1792 cells treated with NMTi (Fig. 4E; Supplementary Fig. S8B), and immunoblotting revealed that AIF was enriched in the nuclear fraction of NMTi-treated, but not control cells (Fig. 4F). Notably, AIF was largely cytoplasmic in control H460 xenografts but mostly nuclear in H460 xenografts from mice treated with PCLX-001 (Fig. 4G; Supplementary Fig. S8D).

To confirm the role of PARP in death induced by NMTi, we used the PARP inhibitor olaparib, a parthanatos inhibitor (59). Treatment of H460 cells with olaparib in the presence of NMTi effectively prevented PARylation (Supplementary Fig. S8C). Notably, despite causing cell death on its own, olaparib decreased NMTi-induced cell death at 72 and 96 hours of treatment (Fig. 4H; Supplementary Fig. S9A–C).

Taken together, our data indicate that NMTis are a new class of parthanatos-inducing drugs with the potential to kill cancer cells that are resistant to other forms of death such as apoptosis or ferroptosis. Furthermore, our findings also indicate that parthanatos may occur downstream of mitochondrial ferrous iron accumulation and that cells undergoing parthanatos may display features associated with ferroptosis, such as excessive lipid peroxidation and accumulation of transferrin receptor at the plasma membrane.

### N-myristoyltransferase inhibition activates the DNA damage response and potentiates cell death induced by platinum doublet chemotherapy in (KL/K)^MUT^ lung carcinoma cells

**Figure 5:**
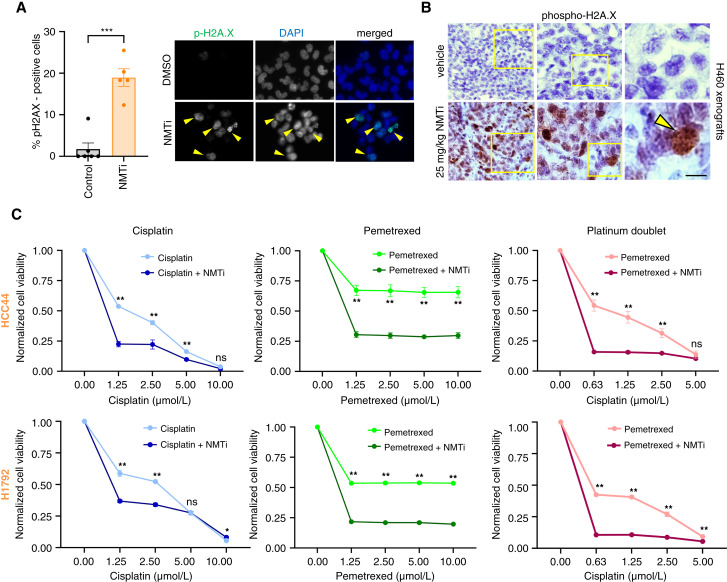
NMTi treatment activates the DNA damage response and sensitizes (KL/K)^MUT^ lung carcinoma cells to platinum doublet chemotherapy. **A,** Phospho-H2A.X staining in (KL/K)^MUT^ H460 lung cancer cells treated with 1 μmol/L PCLX-001 (NMTi) or vehicle control for 72 hours. Graph: percentage of p-H2A.X positive cells calculated from at least 400 cells per condition. Crossbar, group mean; error bars, SEM. ***, *P* < 0.0002 (Student *t* test). Representative images are shown. Arrowheads, nuclei containing p-H2A.X-positive foci. Bar, 25 μm. **B,** Phospho-H2A.X staining of H460 xenograft tumor sections from mice treated with 25 mg/kg PCLX-001 or vehicle control. Square, area magnified for each on the right. Arrowhead, nuclei with p-H2A.X positive foci. Bar, 100, 30, and 10 μm in the left, middle, and right columns, respectively. **C,** Viability test (CCK8) of (KL/K)^MUT^ HCC44 and H1792 cells treated with PCLX-001 (NMTi) in combination with platinum-based chemotherapy. Left, Dose–response of cisplatin in combination with NMTi (50 nmol/L). Middle, Dose–response of pemetrexed in combination with NMTi (50 nmol/L). Right, Dose–response of cisplatin in combination with a single dose of pemetrexed (5 μmol/L) and a single dose of NMTi (125 nmol/L). OD was normalized to vehicle-treated samples. One representative experiment from two independent experiments with the same result is shown. Error bars, SEM. **, *P* < 0.0001; *, *P* < 0.0025, two-way ANOVA with the interaction between drug and dose level. All *P* values are adjusted for multiple testing using the Šídák method.

Parthanatos is characterized by extensive oxidative stress and DNA damage (42). To investigate if NMTi caused DNA damage in (KL/K)^MUT^ lung cancer cells, we stained cells with the DNA damage response marker phospho-H2A.X, which localizes at DNA repair foci. Notably, NMTi increased phospho-H2A.X positive foci in (KL/K)^MUT^ H1792 lung carcinoma (Fig. 5A) and xenografted H460 tumors from mice treated with PCLX-001 (Fig. 5B).

The induction of DNA damage by NMTi suggests that it could sensitize cancer cells to DNA-damaging chemotherapeutics. To test this hypothesis, we selected cisplatin and pemetrexed, chemotherapeutics to which (KL/K)^MUT^ HCC44 and H1792 are resistant (*Genomics of Sensitivity in Cancer*). Treatment with PCLX-001 (50 nmol/L) decreased the viability in response to 1.25 μmol/L cisplatin by ∼25% in HCC44 and by ∼20% in H1792 (Fig. 5C). Treatment with PCLX-001 (50 nmol/L) decreased the viability of in response to 5 μmol/L pemetrexed by ∼35% in HCC44 and ∼25% in H1792 (Fig. 5C). When PCLX-001 (125 nmol/L) was combined with 5 μmol/L pemetrexed, the viability of cells treated with 0.6 μmol/L cisplatin decreased by ∼40% in HCC44 cells and ∼30% in H1792 cells (Fig. 5C).

Taken together, our data indicate that NMTi causes DNA damage in (KL/K)^MUT^ lung carcinoma cells and sensitizes them to platinum doublet chemotherapy. Our findings warrant further investigation of the efficacy of combining platinum-based chemotherapy and NMTi in preclinical models of cancer.

### Mitochondria are a key target of N-myristoyltransferase inhibitors

To investigate how NMTi causes parthanatos in (KL/K)^MUT^ lung carcinoma cells, we wondered which biological processes were altered by NMTi treatment in H1792 cells. Analysis of our global proteomics data in H1792 cells revealed that “*NADH dehydrogenase assembly*” and “*mitochondria respiratory chain complex assembly*” were among the top three biological processes overrepresented in control *versus* NMTi-treated cells (Supplementary Fig. S10A), indicating that mitochondria are a key target of NMTi. In agreement with previous reports (bioRxiv 2021.03.20.436222; refs. 7, 60), the abundance of NDUFAF4, a myristoylated protein that participates in the assembly of the mitochondrial respiratory complex I, decreased after NMT inhibition in (KL/K)^MUT^ cells (Supplementary Fig. S10B). siRNA-mediated silencing of NDUFAF4 in NMTi-sensitive lung carcinoma cells caused mitochondrial fragmentation but did not alter cell viability as demonstrated by lack of morphological features of cell death, including DNA fragmentation (Supplementary Fig. S10C). We concluded that loss of NDUFAF4 is unlikely to be a major contributor to NMTi-induced cell death in (KL/K)^MUT^ lung carcinoma cells.

**Figure 6:**
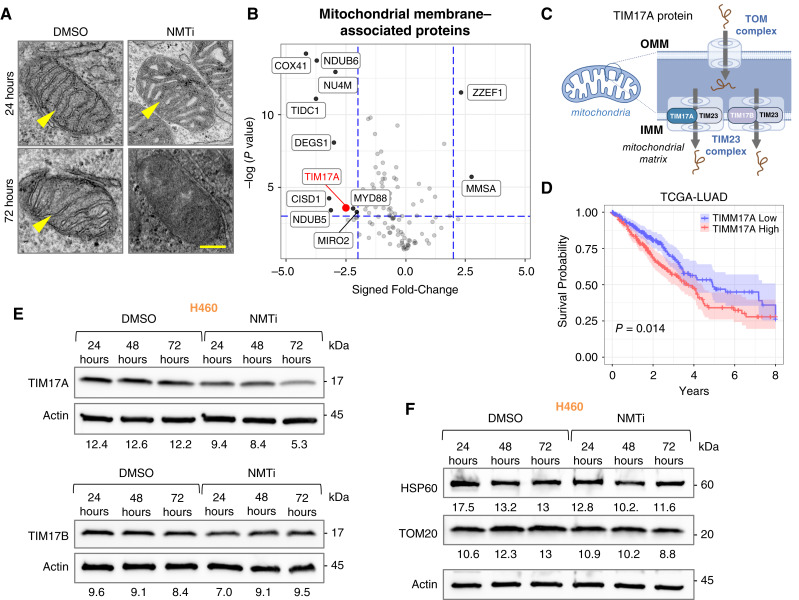
Mitochondria are a key target of NMT inhibition in (KL/K)^MUT^ lung carcinoma cells. **A,** Ultrastructure of mitochondria from (KL/K)^MUT^ H1792 lung carcinoma cells treated with control or 1 μmol/L DDD85646 (NMTi) for the indicated times. Arrowheads, mitochondria cristae. Bar, 50 nm. **B,** Volcano plot of mitochondrial membrane-associated proteins whose abundance was altered by NMTi treatment. Blue lines indicate thresholds for a fold-change of two or a *P* value of 0.05 by moderated *t* test. **C,** Diagram of the main mitochondrial import complexes: TOM, in the outer mitochondrial membrane, and TIM23, in the inner mitochondrial membrane (IMM). TIM17A or TIM17B bind TIM23 to form the main TIM23 complex channel in the IMM. **D,** Kaplan–Meier curve shows percent surviving (overall survival, *y*-axis) over time (years, *x*-axis) for TCGA-LUAD (*n* = 501, *n*-event = 181). High *TIM17A* (Red, ≥50th percentile) were compared with low *TIM17A* (Blue, <50th percentile; log-rank test, *P* = 0.014, median OS was 3.72 years in *TIM17A* High vs. 4.93 in *TIM17A* Low). Colored shading: 95% confidence interval. **E,** Immunoblotting for TIM17A and TIM17B in samples from (KL/K)^MUT^ H460 cells treated with 1 μmol/L PCLX-001 (NMTi) or vehicle control for the indicated times. Numbers, band intensity normalized to actin control. **F,** Immunoblotting for HSP60 and TOM20 in samples from (KL/K)^MUT^ H460 cells treated with 1 μmol/L PCLX-001 (NMTi) or vehicle control for the indicated times. Numbers, band intensity normalized to actin.

Next, we compared the ultrastructure of mitochondria in control and NMTi-treated H1792 cells. After 24 hours of NMTi treatment, mitochondrial transitioned from an orthodox ultrastructure with elongated cristae to a condensed-like ultrastructure in which matrix volume decreases and cristae volume expands (Fig. 6A; Supplementary Fig. S10D), a state associated with transition to increased ATP production (61, 62). Notably, at 72 hours of treatment, mitochondria were highly electron-dense and the vast majority lacked distinguishable internal membranes, an indication of extensive widespread mitochondrial damage (Fig.6A; Supplementary Fig. S10D).

Our differential proteomic data identified 539 proteins from a total of 1,119 mitochondria-related proteins in the Human Protein Atlas. From these, the abundance of 179 (33.2%) was significantly altered by NMTi treatment after adjusting for false discovery: 79 increased and 100 decreased. Gene Ontology analysis showed enrichment in mitochondrial matrix proteins and a decrease in mitochondrial membrane proteins in drug-treated cells (Supplementary Fig. S10E). We reasoned that an imbalance in matrix *versus* membrane mitochondrial protein content might reflect defective import of proteins into the matrix. The outer mitochondrial membrane proteins TOM40 (Translocase of the Outer Mitochondrial Membrane 40) and SAM50 (Sorting and Assembly Machinery Component 50) participate in mitochondrial protein import and are NMT targets (63). SAM50 binds to Coiled-Coil-Helix–Coiled-Coil-Helix Domain Containing 3 (CHCHD3), a component of the mitochondrial contact site and cristae organizing system complex, which is also an NMT target (63). Surprisingly, at 72 hours of NMTi treatment, we found only a modest decrease of around 30% in the abundance of TOM40 and no changes in SAM50 or CHCHD3 in lysates from H1792 cells (Supplementary Fig. S10F). Immunofluorescence revealed that TOM40, SAM50, and CHCHD3 remained largely localized at mitochondria in NMTi-treated cells, also arguing against mis-localization.

Next, we search our proteomics data for other candidates whose loss of function could impact mitochondrial protein import. We focused on mitochondrial membrane-associated proteins and identified 119 mitochondrial membrane proteins from a total of 294 found in the Human Protein Atlas. From these, the abundance of 12 (10%) was significantly altered by NMTi treatment after adjusting for false discovery (Fig. 6B). Of particular interest was the mitochondrial transporter TIM17A, whose abundance was reduced 2.5-fold by NMTi treatment. TIM17 subunits A and B are essential components of the TIM23 inner mitochondrial membrane translocase, one of the two complexes that import nuclear-encoded proteins to the mitochondria (Fig. 6C).

### Dependency on TIM17A is a key determinant of sensitivity to N-myristoyltransferase inhibitors in lung carcinoma

**Figure 7:**
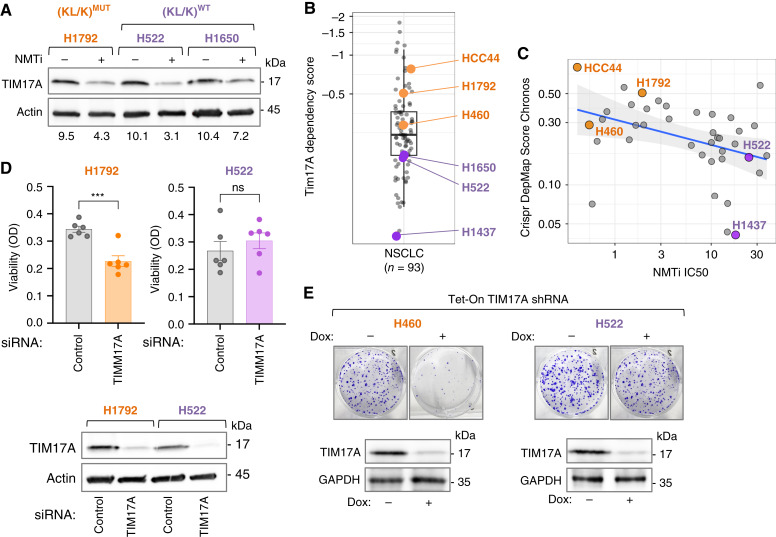
Dependency on TIM17A is a determinant of NMTi sensitivity in lung carcinoma cells. **A,** TIM17A immunoblotting in the indicated cells treated with 1 μmol/L PCLX-001 (NMTi) for 72 hours. Numbers, band intensity normalized to actin control. **B,** Boxplot showing CRISPR DepMap Score for LUAD cells (*y*-axis) estimated using the Chronos algorithm. Negative values suggest decreased cell viability. (KL/K)^MUT^ (HCC44, H460, and H1792) and (KL/K)^WT^ (H522, H1650, and H1437) lung carcinoma cells used in our study are highlighted. **C,** TIM17A CRISPR Chronos scores (*y*-axis) plotted against NMTi IC_50_ (*x*-axis; Spearman’s Correlation, *P* = 0.006, *⍴* = −0.43). Axis scales are log-transformed, and the best-fit line (blue) was calculated using a linear model. Gray shading, standard error of the estimate. H460 was added manually based on our calculated IC_50_ and the publicly available dependency score. **D,** Cell viability (crystal violet staining) in (KL/K)^MUT^ H1792 and (KL/K)^WT^ H522 lung carcinoma cells transfected with a *TIM17*-targeting siRNA pool or a non-targeting control. Bar: average; error bars, SEM. One representative experiment from three independent experiments with similar results. ***, *P* = 0.0009 (Student *t* test). Bottom, TIM17A immunoblotting of the samples above. **E,** Colony assays of (KL/K)^MUT^ H460 and (KL/K)^WT^ H522 expressing Tet-inducible TIM17A or control non-targeting shRNA growing in the presence or absence of Dox. Top, Representative images. Bottom, TIM17A immunoblotting of lysates from the cells used for colony assays. GAPDH, loading control.

We explored the TCGA-LUAD dataset and found a significant association between elevated TIM17A transcript levels and decreased survival probability (Fig. 6D), indicating that TIM17A function may be relevant to lung carcinoma progression. Next, we analyzed TIM17A protein abundance in (KL/K)^MUT^ lung cancer cells treated with NMTi and confirmed that TIM17A levels decreased 24 hours after NMTi treatment in H460 (Fig. 6E) and H1792 (Supplementary Fig. S11A), and 48 hours after treatment in HCC44 cells (Supplementary Fig. S11B). The expression of the TIM17B subunit was only minimally altered by NMTi (Fig. 6E; Supplementary Fig. S11A) even at 72 hours. The mitochondrial matrix protein HSP60 and the transmembrane protein TOM20, which are commonly used mitochondrial markers, remained unchanged or slightly decreased at 72 hours of treatment in H460 (Fig. 6F) or H1792 cells (Supplementary Fig. S11A). Likewise, the mitochondrial membrane protein SAM50 was not altered by NMTi treatment in HCC44 cells (Supplementary Fig. S11B). Resistant (KL/K)^WT^ lung carcinoma cells also showed decreased TIM17A at 72 hours of treatment with NMTi (Fig. 7A), but not at earlier time points (Supplementary Fig. S11C and S11D).

Because NMTi caused loss of TIM17A in sensitive and resistant cells, but only the former died, we reasoned that NMTi-sensitive lung carcinoma cells might have a higher reliance on TIM17A. To explore this possibility, we used the *Cancer Dependency Map* portal (CRISPR Chronos algorithm), which assigns an estimated CRISPR DepMap Score for each gene in a way that negative values suggest decreased cell viability. Notably, TIM17A dependency scores for NMTi-sensitive cells HCC44, H460, and H1792 were lower when compared with the dependency scores of resistant H522, H1650, and H1437 (Fig. 7B). This suggests that (KL/K)^MUT^ lung cancer cells not only are more sensitive to NMTi but also more dependent on TIM17A for survival. Next, we compared NMTi EC_50_ data deposited in the *Genomics of Drug Sensitivity in Cancer* portal with TIM17A dependency data (CRISPR Chronos) deposited in the *Cancer Dependency Map* and found an unexpected significant correlation (*⍴* = −0.43, *P* = 0.006) between NMTi sensitivity and TIM17A dependency in lung carcinoma cells (Fig. 7C).

To demonstrate experimentally that (KL/K)^MUT^ are more dependent on TIM17A than (KL/K)^WT^ cells, we used two complementary genetic strategies. We knocked down TIM17A by transfection of a siRNA pool into H1792 (KL/K)^MUT^ and H522 (KL/K^WT^) cells and analyzed cell viability. In agreement with the CRISPR score data, loss of TIM17A induced cell death in H1792 cells but did not affect the viability of H522 (Fig. 7D). Next, we generated H460 and H522 cells expressing Tet-inducible* TIM17A*****shRNA and used them to evaluate colony formation ability. In agreement with the first approach, colony-forming ability was strongly decreased by TIM17A loss in H460 cells, but only modestly decreased in H522 cells (Fig. 7E). Taken together, our data reveal an unexpected correlation between sensitivity to NMT inhibition and dependency on TIM17A in lung carcinoma cells.

### Genetic targeting of TIM17A induces mitochondrial ferrous iron accumulation and markers of parthanatos in (KL/K)^MUT^ lung carcinoma cells

TIM17A is not myristoylated, thus, it is unclear how NMTi decreases TIM17A levels. We mimicked oxidative stress by treating cells with 4-hydroxynonenal (4-HNE), a byproduct of lipid peroxidation. Notably, a short pulse of 4-HNE caused a modest decrease of TIM17A, accompanied by a compensatory increase in TIM17B abundance in H1792 cells (Supplementary Fig. S12A). Furthermore, a short pulse of 4-HNE caused extensive protein PARylation (Supplementary Fig. S12B), indicating that lipid peroxidation contributes to TIM17A loss and PARP activation in (KL/K)^MUT^ lung carcinoma cells.

**Figure 8:**
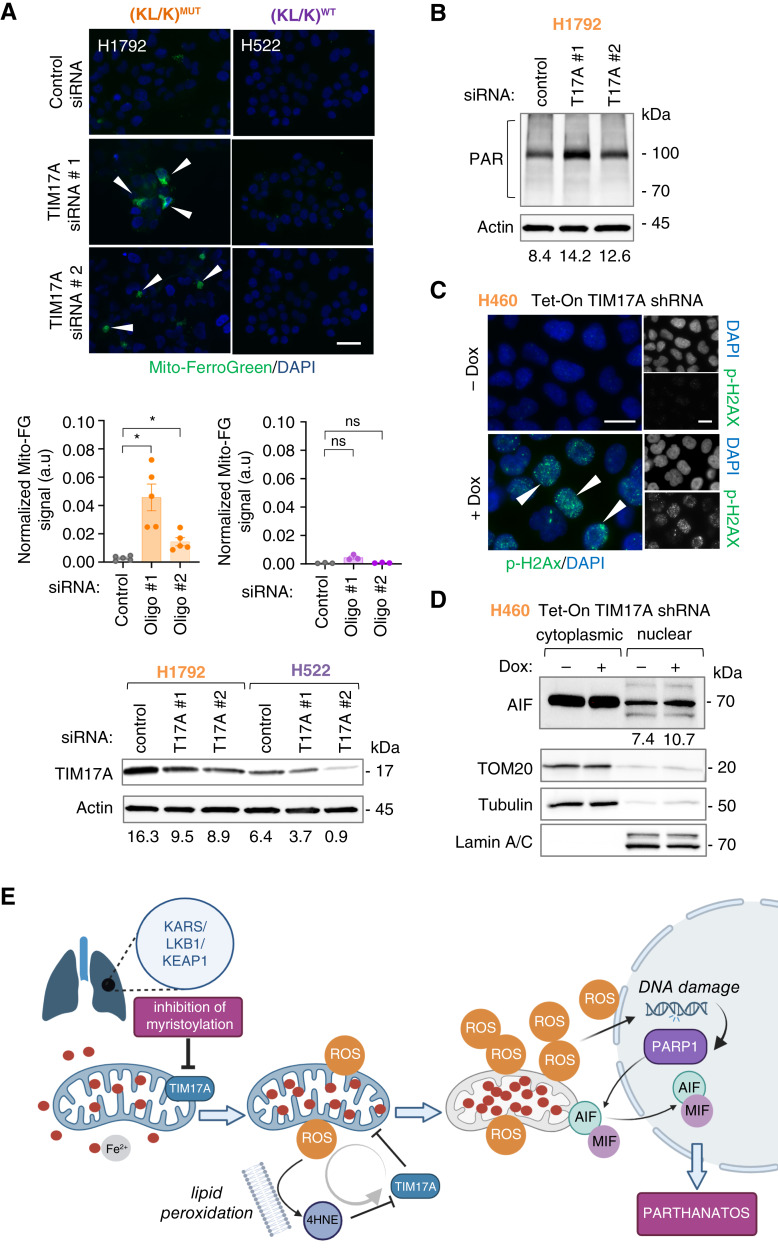
Genetic targeting of TIM17A causes mitochondrial ferrous iron accumulation and activation of parthanatos in (KL/K)^MUT^ lung carcinoma cells. **A,** Mitochondrial ferrous iron detection in (KL/K)^MUT^ and (KL/K)^WT^ cells transfected with non-targeting control or two different TIM17A siRNAs. Quantification of signal intensity on a representative experiment out of two using randomly imaged fields containing at least 480 cells per experimental condition. Graph bars, group mean; error bars, SEM. a.u., arbitrary units. *, *P* = 0.0101 for control vs. #1, *P* = 0.0131 for control vs. #2; ns = not significant (Student *t* test). Representative microscope images are shown. Arrowheads, Mito-FerroGreen positive cells. Bars, 15 μm. Bottom, TIM17A immunoblotting in lysates from the cells used above. Numbers: band intensity normalized to actin control. **B,** Protein PARylation detection by immunoblotting in H1792 cells transfected with non-targeting control and two different TIM17A oligos for 72 hours. Numbers: band intensity normalized to actin control. **C,** H460 cells stably expressing a Tet-inducible TIM17A shRNA treated or not with Dox were stained with p-H2A.X and DAPI. Arrowheads, nuclei containing p-H2A.X-positive foci. Bar, 10 μm. **D,** AIF subcellular localization in (KL/K)^MUT^ H460 cells stably expressing a Tet-inducible TIM17A shRNA treated or not with Dox. Cytoplasmic and nuclear lysates were separated and immunoblotted for AIF. Tubulin, TOM20, and lamin staining were used to verify fraction purity. Numbers: nuclear AIF band intensity normalized to lamin A/B. **E,** Summary of our findings reporting that myristoylation inhibition induces parthanatos through loss of TIM17A and mitochondrial ferrous iron overload in (KL/K)^MUT^ lung carcinoma.

Next, we wondered whether loss of TIM17A in NMTi-sensitive, TIM17A-dependent (KL/K)^MUT^ lung cancer cells could cause mitochondrial ferrous iron overload. We transfected non-targeting control and two different TIM17A-specific siRNA oligos in H1792 (sensitive) and H522 (resistant) cells and analyzed the accumulation of ferrous iron in mitochondria. Notably, sensitive but not resistant cells showed increased mitochondrial ferrous iron when TIM17A was silenced (Fig. 8A). These data suggest that the susceptibility of (KL/K)^MUT^ cells to mitochondrial ferrous iron overload could be a determinant of TIM17A dependency. Accordingly, TIM17A silencing in (KL/K)^MUT^ cells (which induced cell death, Fig. 7D and E) also increased protein PARylation (Fig. 8B), activated the DNA damage response (Fig. 8C) and caused AIF nuclear accumulation (Fig. 8D). Taken together, our data indicate that loss of TIM17A in TIM17A-dependent lung cancer cells causes oxidative stress and contributes to the induction of parthanatos.

We propose a model in which (KL/K)^MUT^ but not (KL/K)^WT^ lung carcinoma cells depend on NMT activity to maintain cellular iron homeostasis. Inhibition of NMT caused mitochondrial iron overload, increased ROS generation, and led to widespread lipid peroxidation and DNA damage by a mechanism at least in part mediated by loss of the mitochondrial transporter subunit TIM17A. Excessive lipid peroxidation, which in turn led to further TIM17A loss in a vicious cycle, caused sustained oxidative stress, activated PARP, and induced parthanatos in (KL/K)^MUT^ lung carcinoma cells (Fig. 8E).

## Discussion

We report here that NMT inhibition causes death by parthanatos in a subset of lung carcinoma cells through TIM17A loss, mitochondrial ferrous iron overload and oxidative stress-induced DNA damage, and PARP activation. We show that NMTis are effective as single agents against lung carcinomas with concurrent LKB1 and/or KEAP1 mutations in a KRAS-mutant background (KL/K)^MUT^ and sensitize cells with these mutations to platinum-based chemotherapy.

NMT1 was previously associated with lower survival in LUAD (64) and our updated TCGA data analysis is consistent with that study. Some lung carcinoma cells included in this study were previously found to be sensitive to PCLX-001 (18).

The reason for the sensitivity of (KL/K)^MUT^ lung carcinoma cells to NMT inhibition is unknown, as none of these tumor drivers is myristoylated. Additional research is necessary to dissect the specific molecular mechanisms by which this mutational signature confers sensitivity to NMTi. Nevertheless, our data indicate that a subset of highly aggressive and difficult-to-treat lung carcinomas rely on NMT activity for survival, uncovering a novel potential therapeutic approach for these aggressive tumors.

It is unclear how NMTi alters the distribution of TfR1, but a similar enrichment in membrane localization of TfR1 occurs in cells undergoing ferroptosis (54), suggesting a role for oxidative stress and lipid peroxidation in the disruption of TfR1 trafficking downstream NMTi treatment.

Mitochondrial ferrous iron overload induced by NMTi seemed linked to TIM17A loss. Understanding the exact mechanism by which TIM17A loss induces ferrous iron accumulation in mitochondria requires further investigation, but we could speculate that decreased mitochondrial protein import caused by loss of TIM17A alters the synthesis of iron–sulfur clusters or heme-containing proteins. Indeed, most genetic diseases causing mitochondrial iron accumulation are linked to defective synthesis of iron–sulfur clusters or heme (65). Likewise, it will be relevant to investigate why the accumulation of ferrous iron in mitochondria in response to NMTi happens in (KL/K)^MUT^ but not (KL/K)^WT^ lung carcinoma cells. One possibility is that (KL/K)^MUT^ cells are more reliant on iron–sulfur clusters and/or heme-containing proteins to sustain their high proliferative rates, and thus, a larger amount of ferrous iron is accumulated in their mitochondria if the assembly of these prosthetic groups into proteins fails. In agreement, heme has been shown to sustain oxidative phosphorylation and tumor progression in lung carcinoma (66, 67).

Our proteomic data indicating decreased expression of various electron transport chain components after NMTi treatment has been noted before (17, 68). This could be a consequence of defective mitochondrial protein import and cause decreased ATP generation. Our observation that NMTi causes a switch in mitochondria from an orthodox to a compact state [which has been linked to increased ATP production (61)] suggests a compensatory effect. In agreement, we previously showed that autophagy and lysosomal-associated degradation are defective in lung carcinoma cells treated with NMTi (16), which could contribute to metabolic stress. In addition, NMTi has been shown to decrease oxygen consumption (bioRxiv 2021.03.20.436222; ref. (61)), suggesting defective oxidative phosphorylation.

Although loss of TIM17A represents a pro-survival mechanism that promotes stress resistance by maintaining mitochondrial proteostasis (34), we show here that some cancer cells are dependent on TIM17A expression for survival, likely because loss of TIM17A in these cells, but not others, causes mitochondrial ferrous iron accumulation and oxidative stress. Thus, TIM17A seems to be a potential therapeutic target in a subset of lung carcinomas and possibility other cancers. It will be important to dissect the mechanism by which NMTi causes TIM17A loss. One possibility is that TIM17A decreases in response to stress caused by a general lack of myristoylation of newly synthesized proteins. In agreement with this hypothesis, NMTi has been shown to induce endoplasmic reticulum stress in cancer cells (17).

We discovered that NMTi causes parthanatos. NMTi leads to PARP1 activation, increased protein PARylation, and translocation of AIF and MIF to the nucleus. The parthanatos inhibitor olaparib, but not inhibitors of apoptosis, ferroptosis, necroptosis, or pyroptosis, partially rescued cell death induced by NMTi. We and others have shown features of apoptosis in cells treated with NMTi (16–18). Although we cannot completely rule out that NMT inhibition causes some apoptosis, the lack of rescue with caspase inhibitors suggests that parthanatos is the main death mechanism downstream of NMTi treatment in (KL/K)^MUT^ lung carcinoma cells. Notably, cells dying by parthanatos have been shown to be Annexin V-positive and experience caspase activation, although it is dispensable for death (42, 43).

Excessive ROS and DNA damage are known activators of PARP in parthanatos. Thus, mitochondrial ferrous iron accumulation and the consequent increase in ROS generation and lipid peroxidation are likely the cause of DNA damage and PARP activation. Notably, we observed that 4-HNE increased PARylation in lung carcinoma cells, suggesting that lipid peroxidation can also activate PARP. Parthanatos is better characterized in neurodegenerative diseases, but it can also occur in cancer cells. As such, the induction of parthanatos could kill apoptosis and ferroptosis-resistant cancer cells, offering obvious therapeutic advantages. However, to date, only a few compounds have been shown to induce parthanatos in cancer (68–70). Our finding that NMTis selectively induce parthanatos in a subset of lung carcinoma cells and possibly other cancer types offers an opportunity to better understand the significance of parthanatos in cancer and how to induce it for therapy.

## Supplementary Material

Figure S1 NMT1 is a therapeutic target in lung carcinoma

Figure S2 Genetic targeting of NMT1 decreases the viability of (KL/K)MUT lung carcinoma.

Figure S3 Inhibition of NMT alters iron homeostasis in (KL/K)MUT lung carcinoma cells.

Figure S4 Inhibition of NMT increases mitochondrial ferrous iron content in (KL/K)MUT but not (KL/K)WT lung carcinoma cells.

Figure S5 Inhibition of NMT increases mitochondrial ferrous iron content in (KL/K)MUT but not (KL/K)WT lung carcinoma cells.

Figure S6 Inhibition of NMT prevents cell cycle progression and causes death in lung carcinoma cells.

Figure S7 Treatment with necroptosis and pyroptosis inhibitors fail to prevent cell death induced by of NMTi treatment in lung carcinoma cells.

Figure S8 NMTi treatment induces features of parthanatos in vitro and in vivo.

Figure S9 The PARP inhibitor olaparib rescues death induced by NMTi treatment.

Figure S10 Mitochondria is a key target of NMT inhibitors.

Figure S11 Effect of NMTi on the abundance of TIM17A in lung carcinoma cells.

Figure S12 Effect of lipid peroxidation on the abundance of TIM17A in lung carcinoma cells.

Supplementary Table 1 MS data of H1792 cells treated with NMTi
