# Supplementary material for: N-Myristoytransferase Inhibition Causes Mitochondrial Iron Overload and Parthanatos in TIM17A-Dependent Aggressive Lung Carcinoma
Source: Cancer Res Commun. 2024 Jul 25;4(7):1815–33. doi: 10.1158/2767-9764.CRC-23-0428 (PMC11270646; doi:10.1158/2767-9764.CRC-23-0428)
Supplement: Figure S1 — NMT1 is a therapeutic target in lung carcinoma [file crc-23-0428_figure_s1_supps1.pptx]

## Slide 1
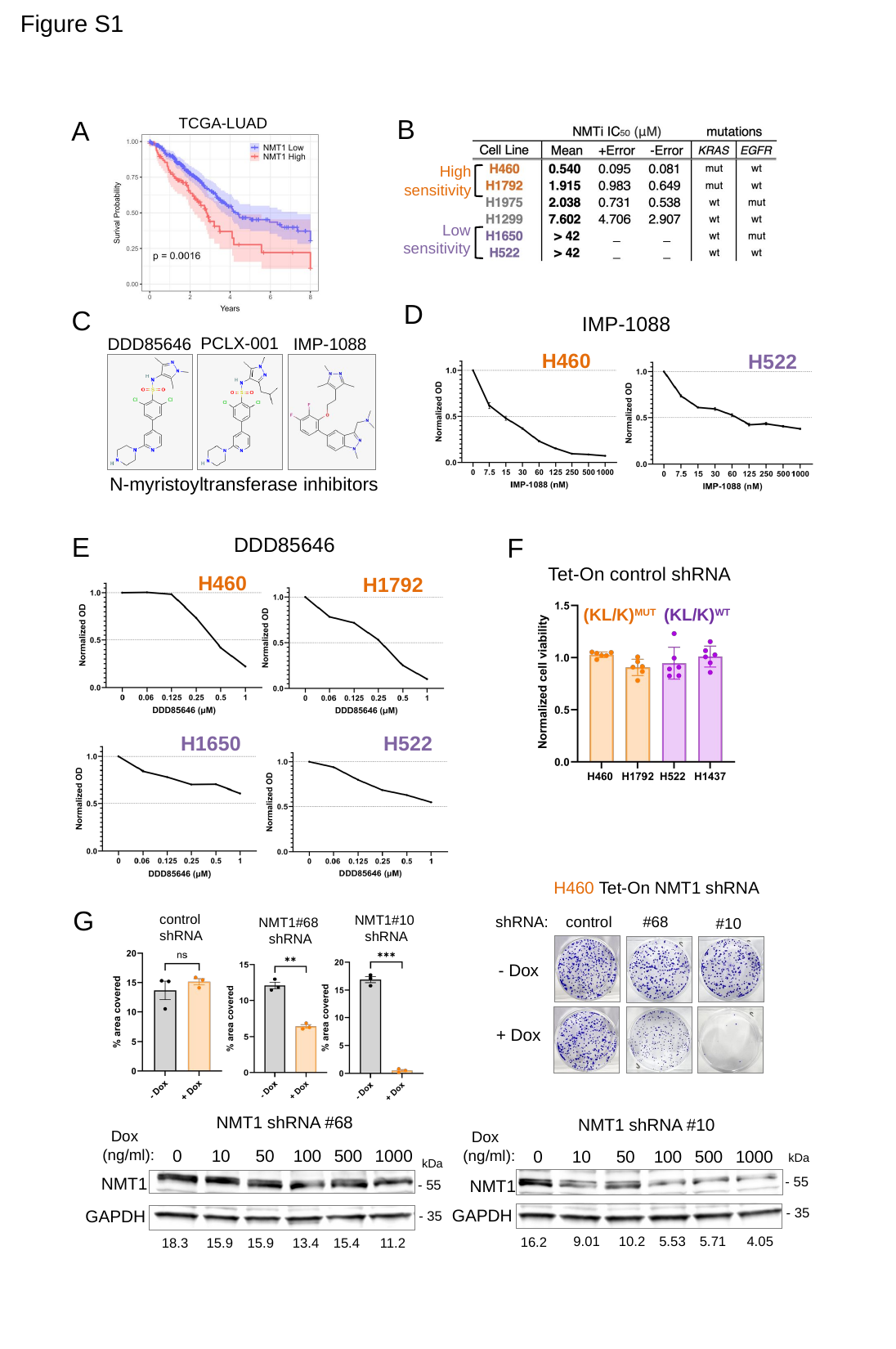

Figure S1
B
TCGA-LUAD
A
High
sensitivity
Low
sensitivity
D
C
IMP-1088
PCLX-001
IMP-1088
DDD85646
N-myristoyltransferase inhibitors
H460
H522
E
F
DDD85646
Tet-On control shRNA
(KL/K)MUT
(KL/K)WT
H460
H1792
H1650
H522
H460 Tet-On NMT1 shRNA
#68
#10
control
shRNA:
- Dox
+ Dox
control
shRNA
NMT1#10
shRNA
NMT1#68
shRNA
G
NMT1 shRNA #68
 Dox
 (ng/ml):
0
10
50
100
500
1000
kDa
NMT1
- 55
GAPDH
- 35
15.9
15.9
13.4
15.4
11.2
18.3
NMT1 shRNA #10
kDa
- 55
NMT1
- 35
GAPDH
9.01
10.2
5.53
5.71
4.05
16.2
 Dox
 (ng/ml):
0
10
50
100
500
1000

## Slide 2
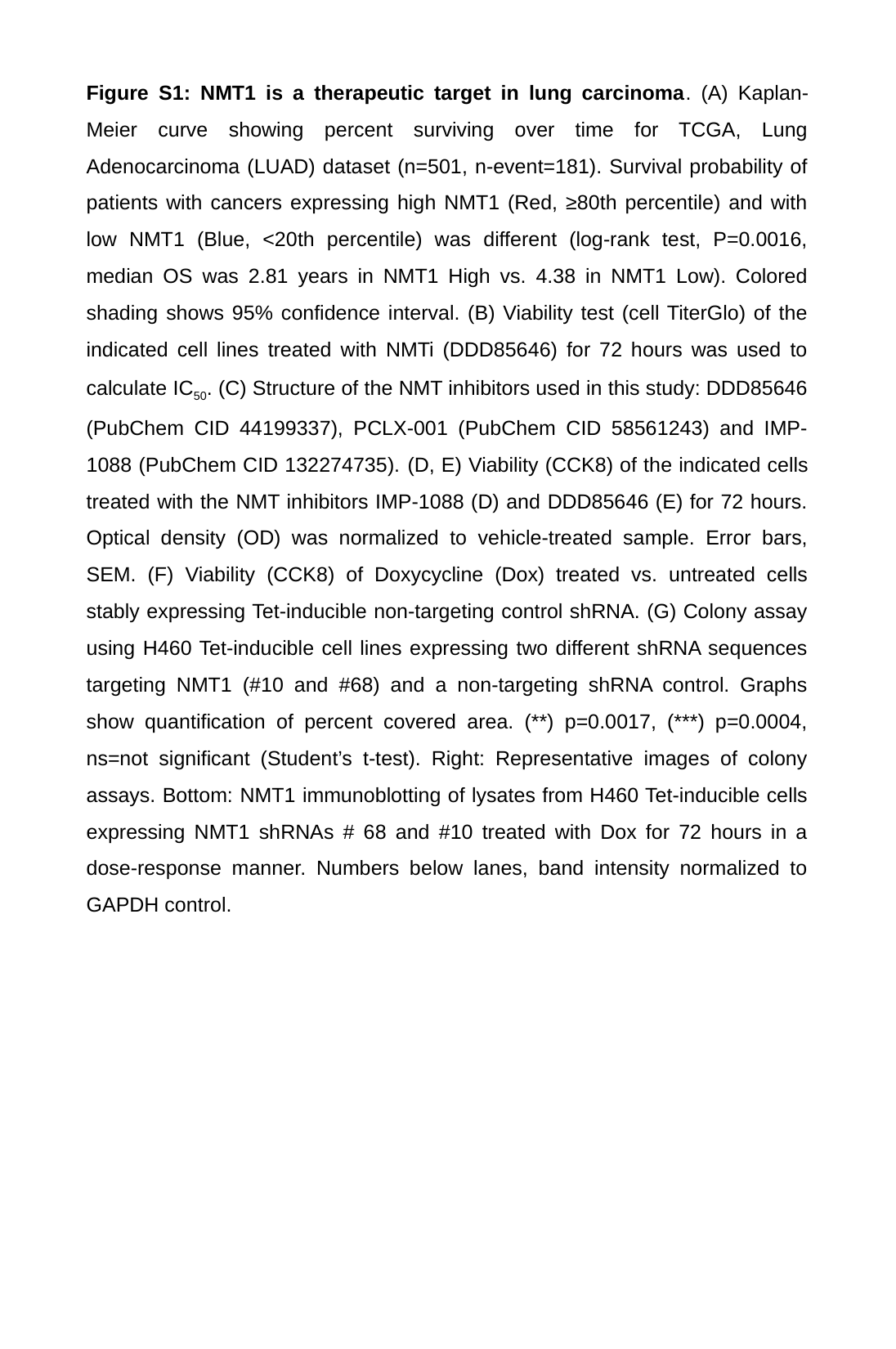

Figure S1: NMT1 is a therapeutic target in lung carcinoma. (A) Kaplan-Meier curve showing percent surviving over time for TCGA, Lung Adenocarcinoma (LUAD) dataset (n=501, n-event=181). Survival probability of patients with cancers expressing high NMT1 (Red, ≥80th percentile) and with low NMT1 (Blue, <20th percentile) was different (log-rank test, P=0.0016, median OS was 2.81 years in NMT1 High vs. 4.38 in NMT1 Low). Colored shading shows 95% confidence interval. (B) Viability test (cell TiterGlo) of the indicated cell lines treated with NMTi (DDD85646) for 72 hours was used to calculate IC50. (C) Structure of the NMT inhibitors used in this study: DDD85646 (PubChem CID 44199337), PCLX-001 (PubChem CID 58561243) and IMP-1088 (PubChem CID 132274735). (D, E) Viability (CCK8) of the indicated cells treated with the NMT inhibitors IMP-1088 (D) and DDD85646 (E) for 72 hours. Optical density (OD) was normalized to vehicle-treated sample. Error bars, SEM. (F) Viability (CCK8) of Doxycycline (Dox) treated vs. untreated cells stably expressing Tet-inducible non-targeting control shRNA. (G) Colony assay using H460 Tet-inducible cell lines expressing two different shRNA sequences targeting NMT1 (#10 and #68) and a non-targeting shRNA control. Graphs show quantification of percent covered area. (**) p=0.0017, (***) p=0.0004, ns=not significant (Student’s t-test). Right: Representative images of colony assays. Bottom: NMT1 immunoblotting of lysates from H460 Tet-inducible cells expressing NMT1 shRNAs # 68 and #10 treated with Dox for 72 hours in a dose-response manner. Numbers below lanes, band intensity normalized to GAPDH control.
