# Supplementary material for: N-Myristoytransferase Inhibition Causes Mitochondrial Iron Overload and Parthanatos in TIM17A-Dependent Aggressive Lung Carcinoma
Source: Cancer Res Commun. 2024 Jul 25;4(7):1815–33. doi: 10.1158/2767-9764.CRC-23-0428 (PMC11270646; doi:10.1158/2767-9764.CRC-23-0428)
Supplement: Figure S2 — Genetic targeting of NMT1 decreases the viability of (KL/K)MUT lung carcinoma. [file crc-23-0428_figure_s2_supps2.pptx]

## Slide 1
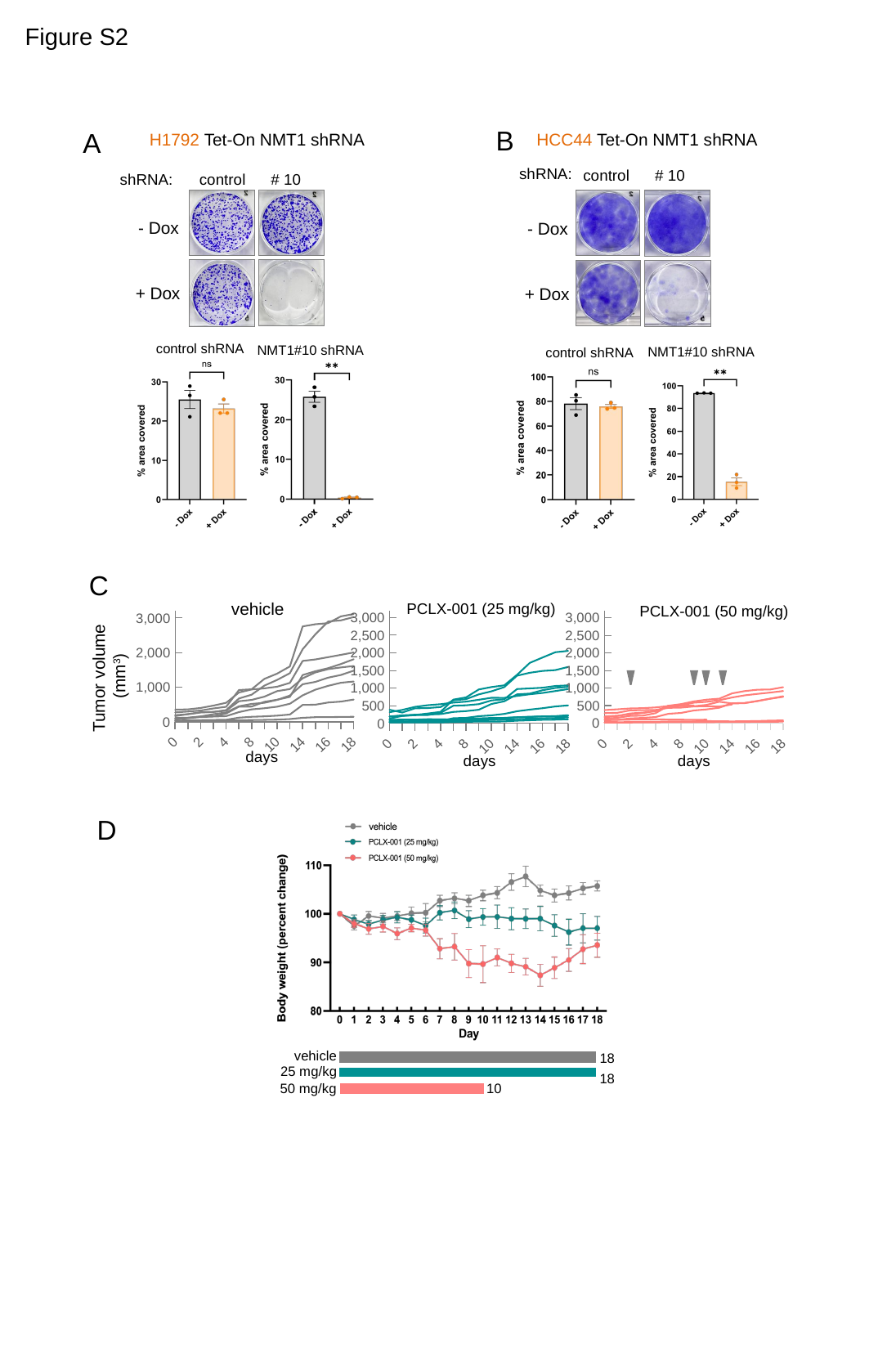

Figure S2
B
A
HCC44 Tet-On NMT1 shRNA
H1792 Tet-On NMT1 shRNA
shRNA:
control
# 10
shRNA:
control
# 10
- Dox
+ Dox
- Dox
+ Dox
control shRNA
NMT1#10 shRNA
NMT1#10 shRNA
control shRNA
C
vehicle
PCLX-001 (25 mg/kg)
PCLX-001 (50 mg/kg)
### Chart
| Category | 10 | 11 | 12 | 13 | 14 | 15 | 16 | 17 | 18 | 19 |
|---|---|---|---|---|---|---|---|---|---|---|
| 0 | 26.263999999999996 | 24.5055 | 51.2 | 89.46450000000002 | 98.39399999999999 | 132.5 | 188.01250000000002 | 192.20000000000002 | 313.44950000000006 | 382.925 |
| 1 | 33.635000000000005 | 23.400000000000002 | 52.0 | 89.46450000000002 | 101.614 | 207.0 | 209.48400000000004 | 224.334 | 378.44999999999993 | 303.24 |
| 2 | 63.504000000000005 | 25.466500000000003 | 57.33 | 96.768 | 104.92750000000001 | 228.84149999999997 | 235.2 | 242.406 | 465.5199999999999 | 423.86399999999986 |
| 3 | 69.82799999999999 | 24.3 | 52.951499999999996 | 113.14349999999999 | 110.592 | 232.56249999999997 | 276.538 | 246.895 | 511.48799999999994 | 431.43299999999994 |
| 4 | 62.775 | 23.1275 | 51.2 | 110.00000000000001 | 111.74399999999999 | 294.03 | 322.45199999999994 | 258.94399999999996 | 541.0174999999999 | 459.418 |
| 7 | 142.97 | 37.56999999999999 | 69.8625 | 112.21600000000002 | 99.40500000000002 | 496.0 | 671.058 | 321.408 | 585.844 | 650.25 |
| 8 | 153.0 | 41.0375 | 78.41950000000001 | 125.388 | 105.984 | 508.4775 | 733.1625 | 344.988 | 612.5625 | 684.2805000000002 |
| 9 | 200.6875 | 46.93 | 82.944 | 137.98399999999998 | 108.288 | 541.282 | 958.8125 | 382.42050000000006 | 665.856 | 818.6034999999999 |
| 10 | 224.26399999999995 | 45.36 | 92.33549999999998 | 149.698 | 128.44000000000003 | 646.624 | 1026.675 | 546.75 | 722.1374999999999 | 905.5935 |
| 11 | 264.6525 | 51.26199999999999 | 104.97600000000001 | 153.06199999999998 | 146.71249999999998 | 680.4 | 1076.236 | 622.7280000000001 | 719.2640000000001 | 1024.9375 |
| 14 | 340.70399999999995 | 72.60000000000001 | 116.032 | 171.0 | 170.56900000000002 | 970.2 | 1376.2560000000003 | 820.228 | 775.284 | 1351.6800000000003 |
| 15 | 386.63 | 83.94200000000001 | 127.832 | 178.60799999999998 | 183.6 | 988.768 | 1715.0 | 841.5014999999999 | 822.3120000000001 | 1432.9434999999999 |
| 16 | 426.27500000000003 | 96.25 | 138.6 | 194.56000000000003 | 193.49199999999996 | 1007.512 | 1866.2400000000002 | 936.396 | 859.0999999999999 | 1485.8760000000002 |
| 17 | 476.2560000000001 | 101.4 | 146.97949999999997 | 192.49649999999997 | 202.41899999999998 | 1057.409 | 2015.1680000000001 | 1000.6920000000001 | 912.9835000000002 | 1508.304 |
| 18 | 506.25 | 113.4375 | 164.7135 | 207.025 | 228.68999999999997 | 1075.2305000000001 | 2053.5925 | 1031.55 | 968.832 | 1599.6960000000004 |
### Chart
| Category | 20 | 21 | | 22 | | 23 | 24 | | 28 | 25 |
|---|---|---|---|---|---|---|---|---|---|---|
| 0 | 16.038000000000004 | 21.150000000000002 | 28.88 | 86.43600000000002 | 97.19600000000003 | 126.15 | 166.20799999999997 | 191.664 | 284.752 | 372.4 |
| 1 | 15.6735 | 27.165999999999997 | 32.49 | 93.28800000000001 | 97.92 | 154.791 | 204.65499999999997 | 212.70399999999995 | 295.3125 | 393.984 |
| 2 | 18.9225 | 31.752000000000006 | None | 134.56 | 101.376 | 207.10350000000003 | 276.85 | 248.832 | 364.8 | 418.91400000000004 |
| 3 | 24.064000000000007 | 32.400000000000006 | None | 150.70049999999998 | 101.614 | 214.24500000000003 | 300.672 | 288.8 | 370.6965 | 427.5 |
| 4 | 23.040000000000006 | 37.544 | None | 175.3375 | 109.44 | 284.59200000000004 | 343.672 | 344.4525 | 377.2575 | 447.63950000000006 |
| 7 | 29.947499999999998 | 47.150999999999996 | None | 267.1875 | 103.684 | 496.375 | 496.375 | 473.8125 | 435.2174999999999 | 486.4 |
| 8 | 27.648000000000007 | 52.111 | None | 292.032 | 101.25 | 542.6260000000002 | 505.4 | 485.9800000000001 | 442.7864999999999 | 492.075 |
| 9 | 26.427500000000002 | 52.0 | None | 361.67250000000007 | 94.29899999999999 | 619.038 | 583.1595000000001 | 505.4 | 475.26000000000005 | 480.0 |
| 10 | 26.908 | 59.094 | None | 391.98799999999994 | 100.67200000000003 | 668.3670000000001 | 606.9594999999999 | None | 483.18100000000004 | 520.1195000000001 |
| 11 | 32.946 | 57.33 | None | 439.61550000000005 | None | 697.6320000000001 | 654.368 | None | 472.38400000000007 | 610.17 |
| 14 | 34.102 | 52.0 | None | 566.6360000000002 | None | 846.7199999999999 | 730.34 | None | 533.2319999999999 | 559.9375 |
| 15 | 36.75 | 56.3135 | None | 576.2400000000001 | None | 912.5250000000001 | 792.55 | None | None | 569.492 |
| 16 | 37.975 | 60.858000000000004 | None | 629.4419999999999 | None | 951.3855 | 834.1759999999999 | None | None | 631.8 |
| 17 | 42.120000000000005 | 73.9125 | None | 696.6320000000001 | None | 958.2299999999999 | 870.7320000000001 | None | None | 698.0440000000001 |
| 18 | 42.768 | 79.35 | None | 766.3245000000001 | None | 1022.4 | 915.008 | None | None | 748.0155 |
### Chart
| Category | 1 | 2 | 3 | 4 | 5 | 6 | 7 | 8 | 9 |
|---|---|---|---|---|---|---|---|---|---|
| 0 | 18.0 | 27.380000000000003 | 60.75 | 92.512 | 105.84 | 162.0 | 189.48599999999996 | 268.363 | 346.79999999999995 |
| 1 | 19.968000000000004 | 39.503499999999995 | 103.933 | 106.1705 | 116.16000000000003 | 219.006 | 215.016 | 294.4 | 357.07500000000005 |
| 2 | 26.567999999999998 | 44.982 | 135.0 | 144.86849999999998 | 153.216 | 273.8 | 265.586 | 322.75199999999995 | 396.576 |
| 3 | 29.808 | 50.274 | 147.99400000000003 | 158.4375 | 217.8 | 281.25 | 284.0625 | 383.3280000000001 | 465.46000000000004 |
| 4 | 31.752000000000006 | 54.5455 | 161.79200000000003 | 236.25 | 268.0425 | 334.62 | 317.2015 | 430.61199999999997 | 546.0875 |
| 7 | 44.0 | 116.272 | 276.82199999999995 | 433.34999999999997 | 446.48999999999995 | 671.058 | 591.3 | 915.0625 | 843.8380000000002 |
| 8 | 47.06799999999999 | 142.175 | 356.328 | 437.40000000000003 | 514.5 | 781.4880000000002 | 630.5679999999999 | 937.024 | 931.4909999999999 |
| 9 | 52.6965 | 154.32750000000001 | 388.28999999999996 | 575.0 | 546.912 | 1044.0 | 723.4559999999999 | 1239.3 | 964.4224999999999 |
| 10 | 58.725 | 174.6 | 433.34999999999997 | 636.5400000000001 | 630.0 | 1209.675 | 884.3399999999999 | 1391.6 | 1011.4674999999999 |
| 11 | 75.63150000000002 | 206.84800000000004 | 518.9375 | 716.625 | 756.4499999999999 | 1403.325 | 942.6375 | 1598.7 | 1123.632 |
| 14 | 112.36 | 485.514 | 761.3584999999999 | 1350.36 | 1082.41 | 2096.976 | 1256.375 | 2756.8640000000005 | 1757.8125 |
| 15 | 129.96 | 492.075 | 925.75 | 1461.184 | 1152.0 | 2528.75 | 1425.6000000000001 | 2819.6099999999997 | 1801.926 |
| 16 | 131.5845 | 554.5645000000002 | 1036.8 | 1548.4 | 1275.0 | 2899.0860000000002 | 1520.4645000000003 | 2851.5600000000004 | 1867.7760000000005 |
| 17 | 137.92399999999998 | 582.12 | 1127.1105000000002 | 1669.2480000000003 | 1352.6475000000003 | 2930.7700000000004 | 1570.3125 | 3047.4079999999994 | 1935.05 |
| 18 | 141.288 | 639.5804999999998 | 1164.0625 | 1807.0800000000002 | 1476.9209999999998 | 3027.8744999999994 | 1611.414 | 3114.4770000000008 | 2003.7599999999998 |Tumor volume
 (mm3)
days
days
days
D
vehicle
18
25 mg/kg
18
50 mg/kg
10

## Slide 2
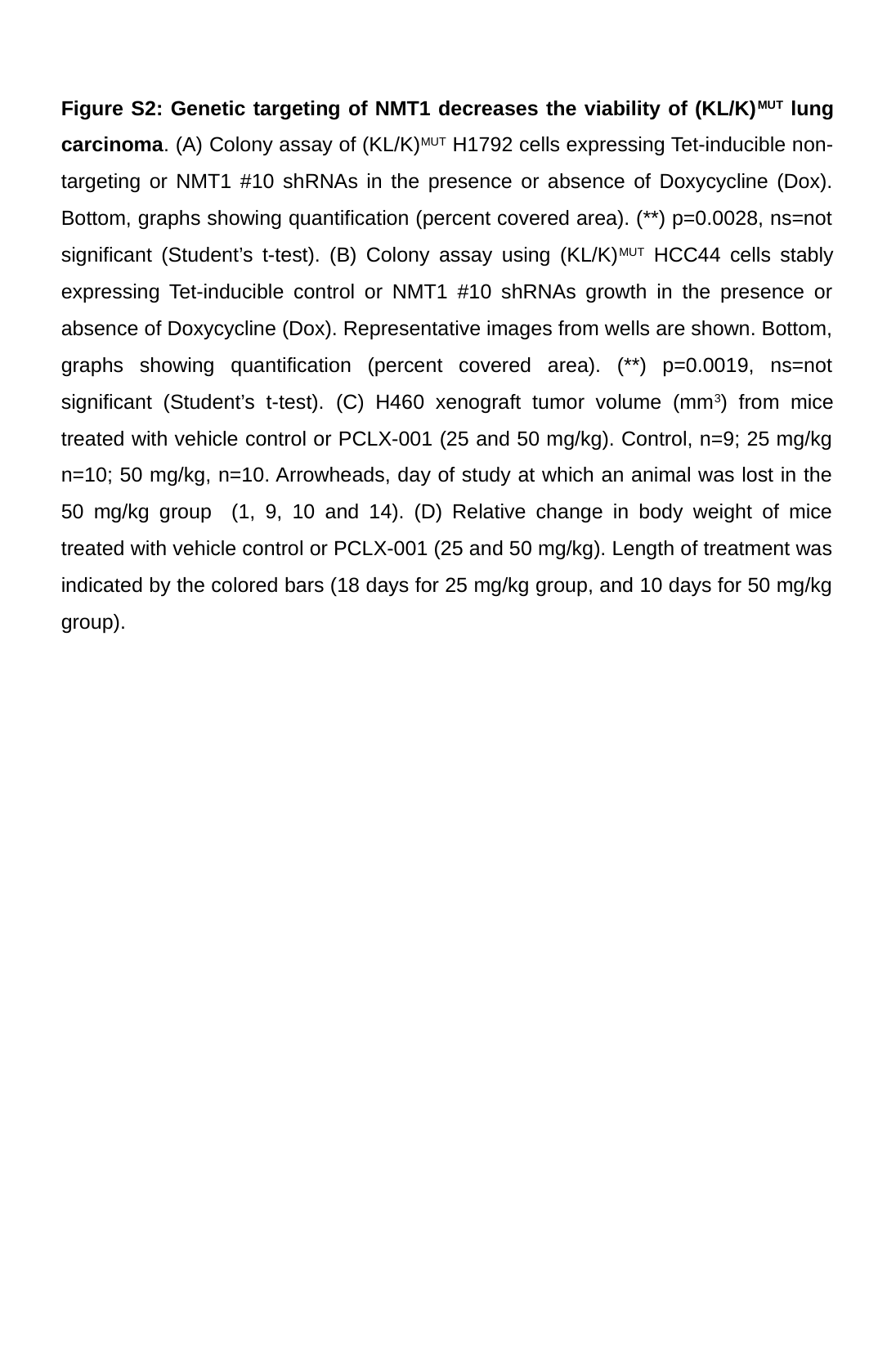

Figure S2: Genetic targeting of NMT1 decreases the viability of (KL/K)MUT lung carcinoma. (A) Colony assay of (KL/K)MUT H1792 cells expressing Tet-inducible non-targeting or NMT1 #10 shRNAs in the presence or absence of Doxycycline (Dox). Bottom, graphs showing quantification (percent covered area). (**) p=0.0028, ns=not significant (Student’s t-test). (B) Colony assay using (KL/K)MUT HCC44 cells stably expressing Tet-inducible control or NMT1 #10 shRNAs growth in the presence or absence of Doxycycline (Dox). Representative images from wells are shown. Bottom, graphs showing quantification (percent covered area). (**) p=0.0019, ns=not significant (Student’s t-test). (C) H460 xenograft tumor volume (mm3) from mice treated with vehicle control or PCLX-001 (25 and 50 mg/kg). Control, n=9; 25 mg/kg n=10; 50 mg/kg, n=10. Arrowheads, day of study at which an animal was lost in the 50 mg/kg group (1, 9, 10 and 14). (D) Relative change in body weight of mice treated with vehicle control or PCLX-001 (25 and 50 mg/kg). Length of treatment was indicated by the colored bars (18 days for 25 mg/kg group, and 10 days for 50 mg/kg group).
