# Supplementary material for: N-Myristoytransferase Inhibition Causes Mitochondrial Iron Overload and Parthanatos in TIM17A-Dependent Aggressive Lung Carcinoma
Source: Cancer Res Commun. 2024 Jul 25;4(7):1815–33. doi: 10.1158/2767-9764.CRC-23-0428 (PMC11270646; doi:10.1158/2767-9764.CRC-23-0428)
Supplement: Figure S3 — Inhibition of NMT alters iron homeostasis in (KL/K)MUT lung carcinoma cells. [file crc-23-0428_figure_s3_supps3.pptx]

## Slide 1
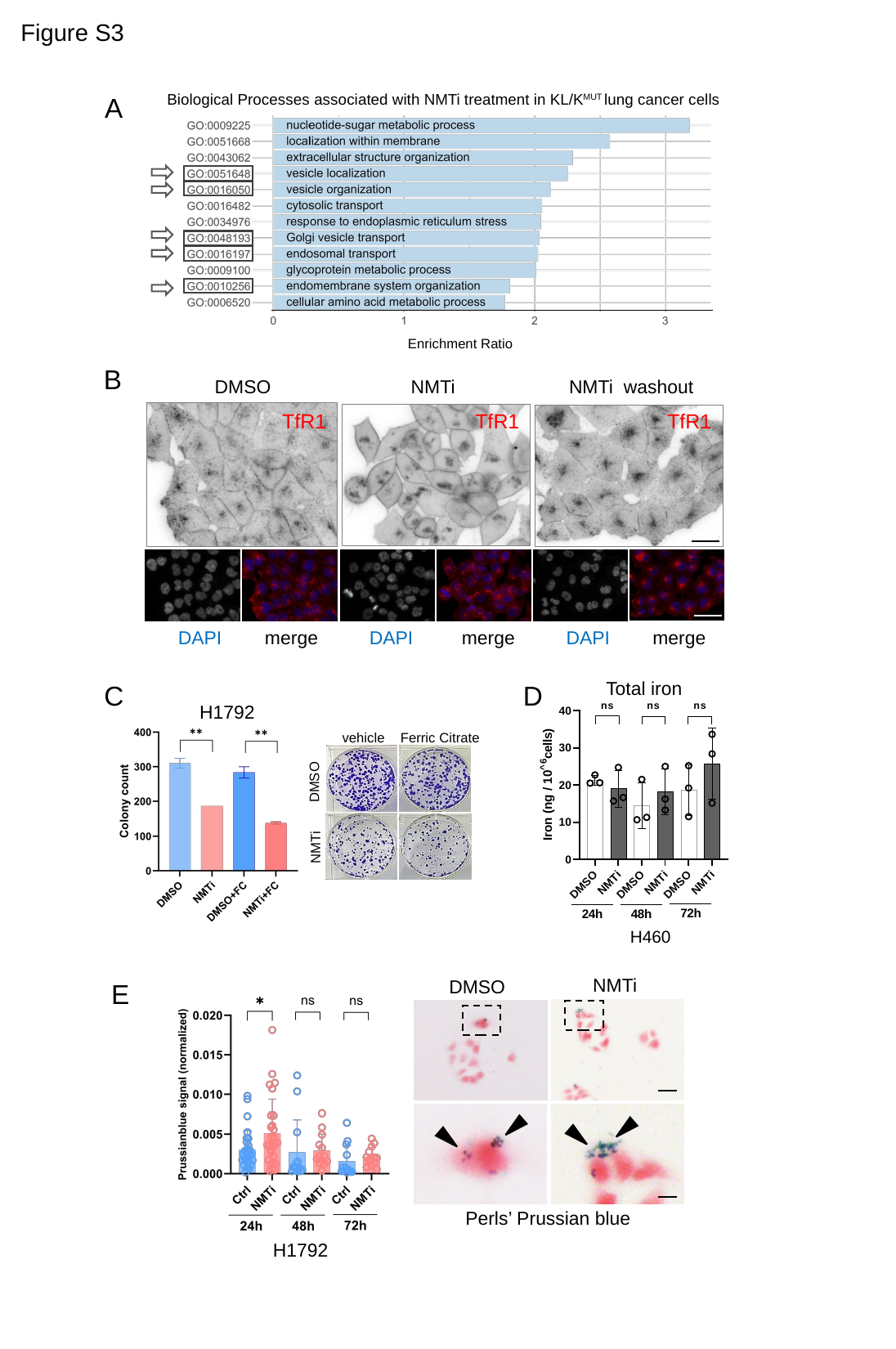

Figure S3
Biological Processes associated with NMTi treatment in KL/KMUT lung cancer cells
 Enrichment Ratio
A
B
DMSO
NMTi
NMTi washout
TfR1
TfR1
TfR1
DAPI
merge
DAPI
merge
DAPI
merge
Total iron
H460
C
D
H1792
vehicle
Ferric Citrate
DMSO
NMTi
NMTi
DMSO
Perls’ Prussian blue
E
H1792

## Slide 2
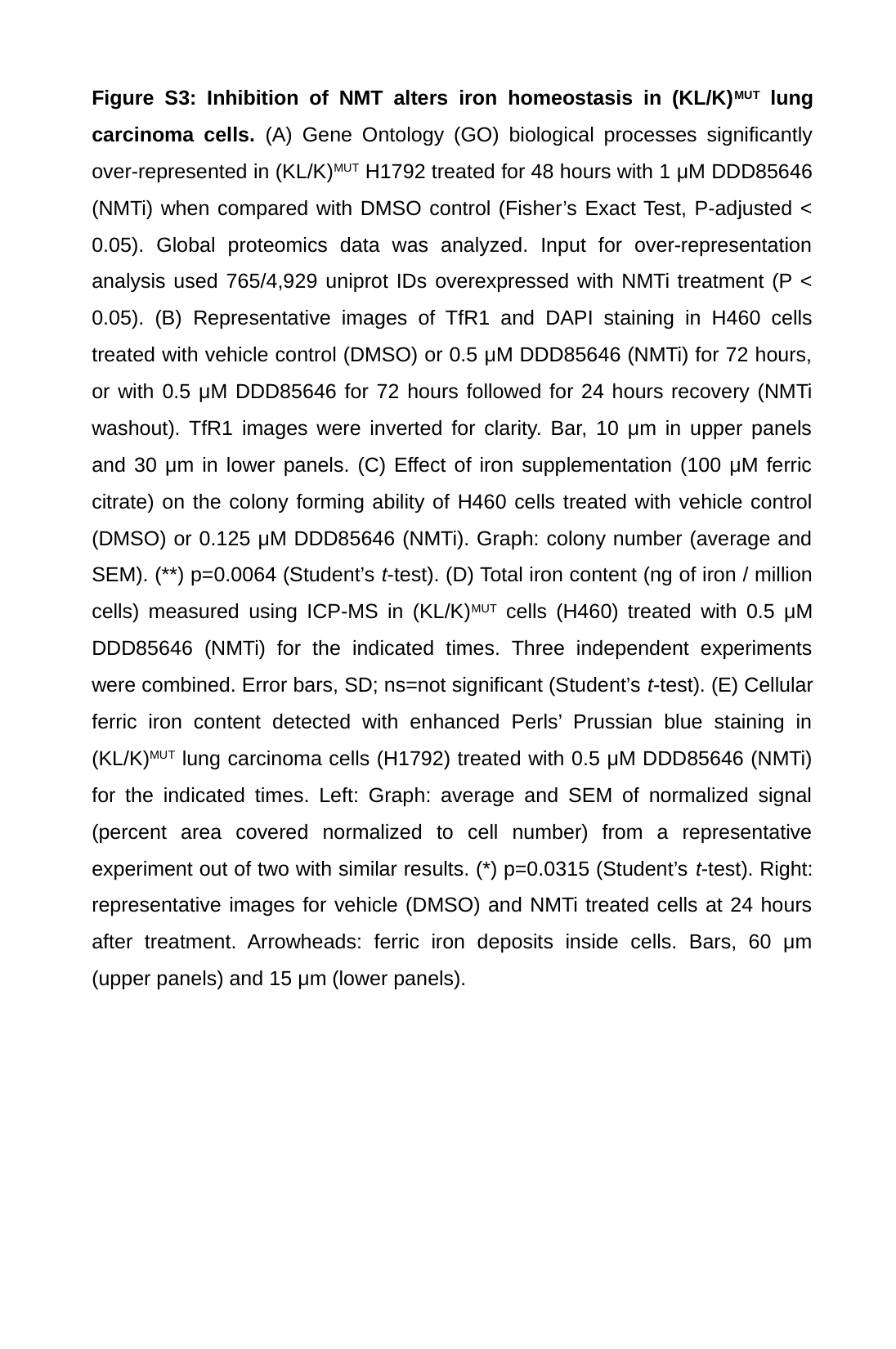

Figure S3: Inhibition of NMT alters iron homeostasis in (KL/K)MUT lung carcinoma cells. (A) Gene Ontology (GO) biological processes significantly over-represented in (KL/K)MUT H1792 treated for 48 hours with 1 μM DDD85646 (NMTi) when compared with DMSO control (Fisher’s Exact Test, P-adjusted < 0.05). Global proteomics data was analyzed. Input for over-representation analysis used 765/4,929 uniprot IDs overexpressed with NMTi treatment (P < 0.05). (B) Representative images of TfR1 and DAPI staining in H460 cells treated with vehicle control (DMSO) or 0.5 μM DDD85646 (NMTi) for 72 hours, or with 0.5 μM DDD85646 for 72 hours followed for 24 hours recovery (NMTi washout). TfR1 images were inverted for clarity. Bar, 10 μm in upper panels and 30 μm in lower panels. (C) Effect of iron supplementation (100 μM ferric citrate) on the colony forming ability of H460 cells treated with vehicle control (DMSO) or 0.125 μM DDD85646 (NMTi). Graph: colony number (average and SEM). (**) p=0.0064 (Student’s t-test). (D) Total iron content (ng of iron / million cells) measured using ICP-MS in (KL/K)MUT cells (H460) treated with 0.5 μM DDD85646 (NMTi) for the indicated times. Three independent experiments were combined. Error bars, SD; ns=not significant (Student’s t-test). (E) Cellular ferric iron content detected with enhanced Perls’ Prussian blue staining in (KL/K)MUT lung carcinoma cells (H1792) treated with 0.5 μM DDD85646 (NMTi) for the indicated times. Left: Graph: average and SEM of normalized signal (percent area covered normalized to cell number) from a representative experiment out of two with similar results. (*) p=0.0315 (Student’s t-test). Right: representative images for vehicle (DMSO) and NMTi treated cells at 24 hours after treatment. Arrowheads: ferric iron deposits inside cells. Bars, 60 μm (upper panels) and 15 μm (lower panels).
