# Supplementary material for: N-Myristoytransferase Inhibition Causes Mitochondrial Iron Overload and Parthanatos in TIM17A-Dependent Aggressive Lung Carcinoma
Source: Cancer Res Commun. 2024 Jul 25;4(7):1815–33. doi: 10.1158/2767-9764.CRC-23-0428 (PMC11270646; doi:10.1158/2767-9764.CRC-23-0428)
Supplement: Figure S4 — Inhibition of NMT increases mitochondrial ferrous iron content in (KL/K)MUT but not (KL/K)WT lung carcinoma cells. [file crc-23-0428_figure_s4_supps4.pptx]

## Slide 1
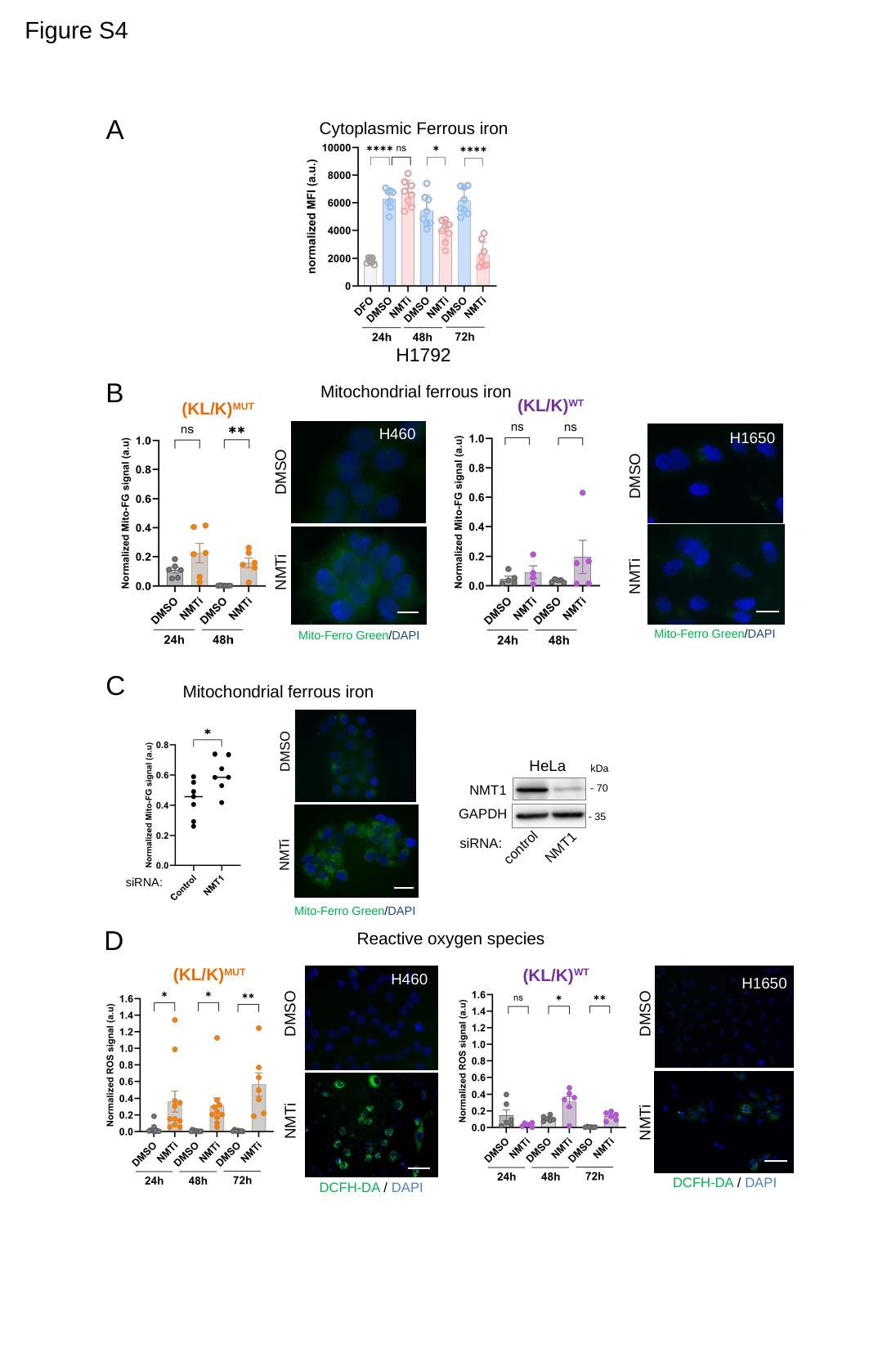

Figure S4
A
Cytoplasmic Ferrous iron
H1792
B
Mitochondrial ferrous iron
(KL/K)WT
(KL/K)MUT
H460
H1650
DMSO
DMSO
NMTi
NMTi
Mito-Ferro Green/DAPI
Mito-Ferro Green/DAPI
C
Mitochondrial ferrous iron
siRNA:
DMSO
HeLa
kDa
- 70
NMT1
GAPDH
- 35
siRNA:
NMT1
control
NMTi
Mito-Ferro Green/DAPI
D
Reactive oxygen species
(KL/K)MUT
(KL/K)WT
H460
DCFH-DA / DAPI
DCFH-DA / DAPI
H1650
DMSO
DMSO
HeLa
NMTi
NMTi

## Slide 2
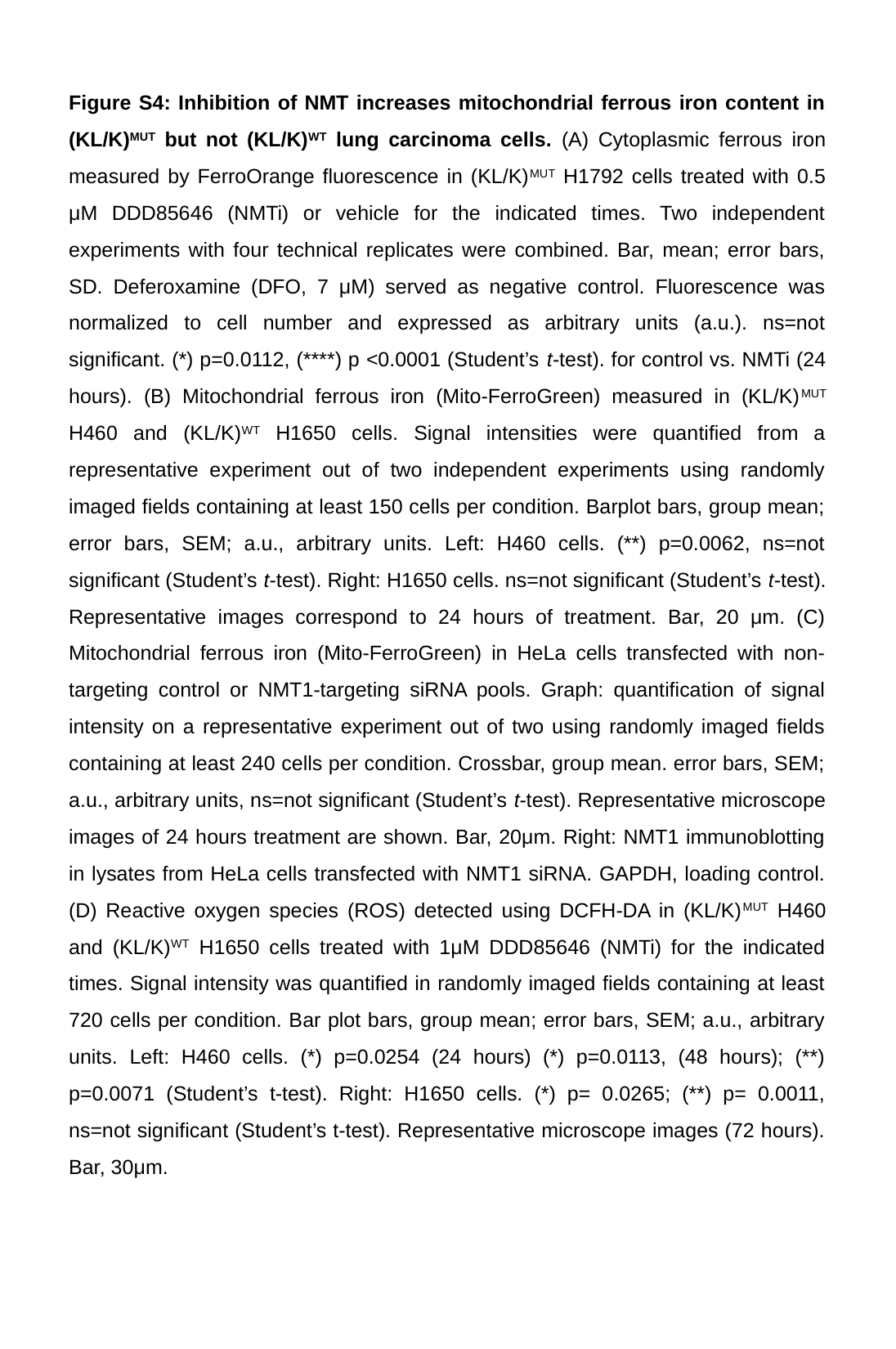

Figure S4: Inhibition of NMT increases mitochondrial ferrous iron content in (KL/K)MUT but not (KL/K)WT lung carcinoma cells. (A) Cytoplasmic ferrous iron measured by FerroOrange fluorescence in (KL/K)MUT H1792 cells treated with 0.5 μM DDD85646 (NMTi) or vehicle for the indicated times. Two independent experiments with four technical replicates were combined. Bar, mean; error bars, SD. Deferoxamine (DFO, 7 μM) served as negative control. Fluorescence was normalized to cell number and expressed as arbitrary units (a.u.). ns=not significant. (*) p=0.0112, (****) p <0.0001 (Student’s t-test). for control vs. NMTi (24 hours). (B) Mitochondrial ferrous iron (Mito-FerroGreen) measured in (KL/K)MUT H460 and (KL/K)WT H1650 cells. Signal intensities were quantified from a representative experiment out of two independent experiments using randomly imaged fields containing at least 150 cells per condition. Barplot bars, group mean; error bars, SEM; a.u., arbitrary units. Left: H460 cells. (**) p=0.0062, ns=not significant (Student’s t-test). Right: H1650 cells. ns=not significant (Student’s t-test). Representative images correspond to 24 hours of treatment. Bar, 20 μm. (C) Mitochondrial ferrous iron (Mito-FerroGreen) in HeLa cells transfected with non-targeting control or NMT1-targeting siRNA pools. Graph: quantification of signal intensity on a representative experiment out of two using randomly imaged fields containing at least 240 cells per condition. Crossbar, group mean. error bars, SEM; a.u., arbitrary units, ns=not significant (Student’s t-test). Representative microscope images of 24 hours treatment are shown. Bar, 20μm. Right: NMT1 immunoblotting in lysates from HeLa cells transfected with NMT1 siRNA. GAPDH, loading control. (D) Reactive oxygen species (ROS) detected using DCFH-DA in (KL/K)MUT H460 and (KL/K)WT H1650 cells treated with 1μM DDD85646 (NMTi) for the indicated times. Signal intensity was quantified in randomly imaged fields containing at least 720 cells per condition. Bar plot bars, group mean; error bars, SEM; a.u., arbitrary units. Left: H460 cells. (*) p=0.0254 (24 hours) (*) p=0.0113, (48 hours); (**) p=0.0071 (Student’s t-test). Right: H1650 cells. (*) p= 0.0265; (**) p= 0.0011, ns=not significant (Student’s t-test). Representative microscope images (72 hours). Bar, 30μm.
