# Supplementary material for: N-Myristoytransferase Inhibition Causes Mitochondrial Iron Overload and Parthanatos in TIM17A-Dependent Aggressive Lung Carcinoma
Source: Cancer Res Commun. 2024 Jul 25;4(7):1815–33. doi: 10.1158/2767-9764.CRC-23-0428 (PMC11270646; doi:10.1158/2767-9764.CRC-23-0428)
Supplement: Figure S5 — Inhibition of NMT increases mitochondrial ferrous iron content in (KL/K)MUT but not (KL/K)WT lung carcinoma cells. [file crc-23-0428_figure_s5_supps5.pptx]

## Slide 1
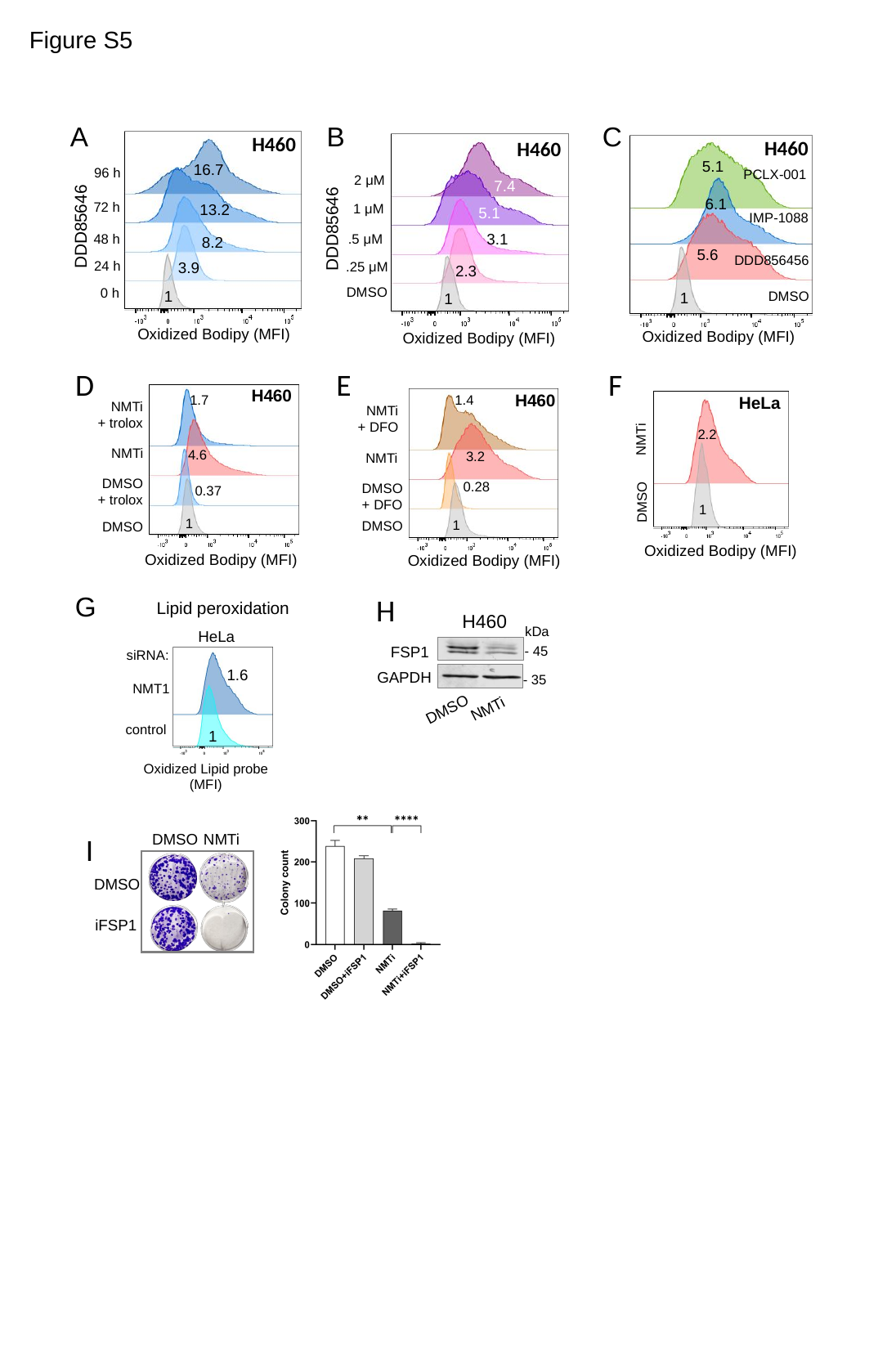

Figure S5
A
B
C
16.7
13.2
8.2
3.9
1
H460
Oxidized Bodipy (MFI)
96 h
72 h
48 h
24 h
0 h
DDD85646
2 μM
7.4
1 μM
5.1
DDD85646
3.1
.5 μM
.25 μM
2.3
DMSO
1
Oxidized Bodipy (MFI)
H460
H460
5.1
PCLX-001
6.1
IMP-1088
5.6
DDD856456
1
Oxidized Bodipy (MFI)
DMSO
D
E
F
H460
1.7
4.6
0.37
1
NMTi
+ trolox
NMTi
DMSO
 + trolox
DMSO
Oxidized Bodipy (MFI)
1.4
3.2
0.28
1
H460
NMTi
+ DFO
NMTi
DMSO + DFO
DMSO
Oxidized Bodipy (MFI)
HeLa
Oxidized Bodipy (MFI)
2.2
1
NMTi
DMSO
G
H
Lipid peroxidation
H460
kDa
FSP1
- 45
GAPDH
- 35
NMTi
DMSO
HeLa
siRNA:
1.6
NMT1
control
1
Oxidized Lipid probe (MFI)
DMSO
NMTi
DMSO
iFSP1
I

## Slide 2
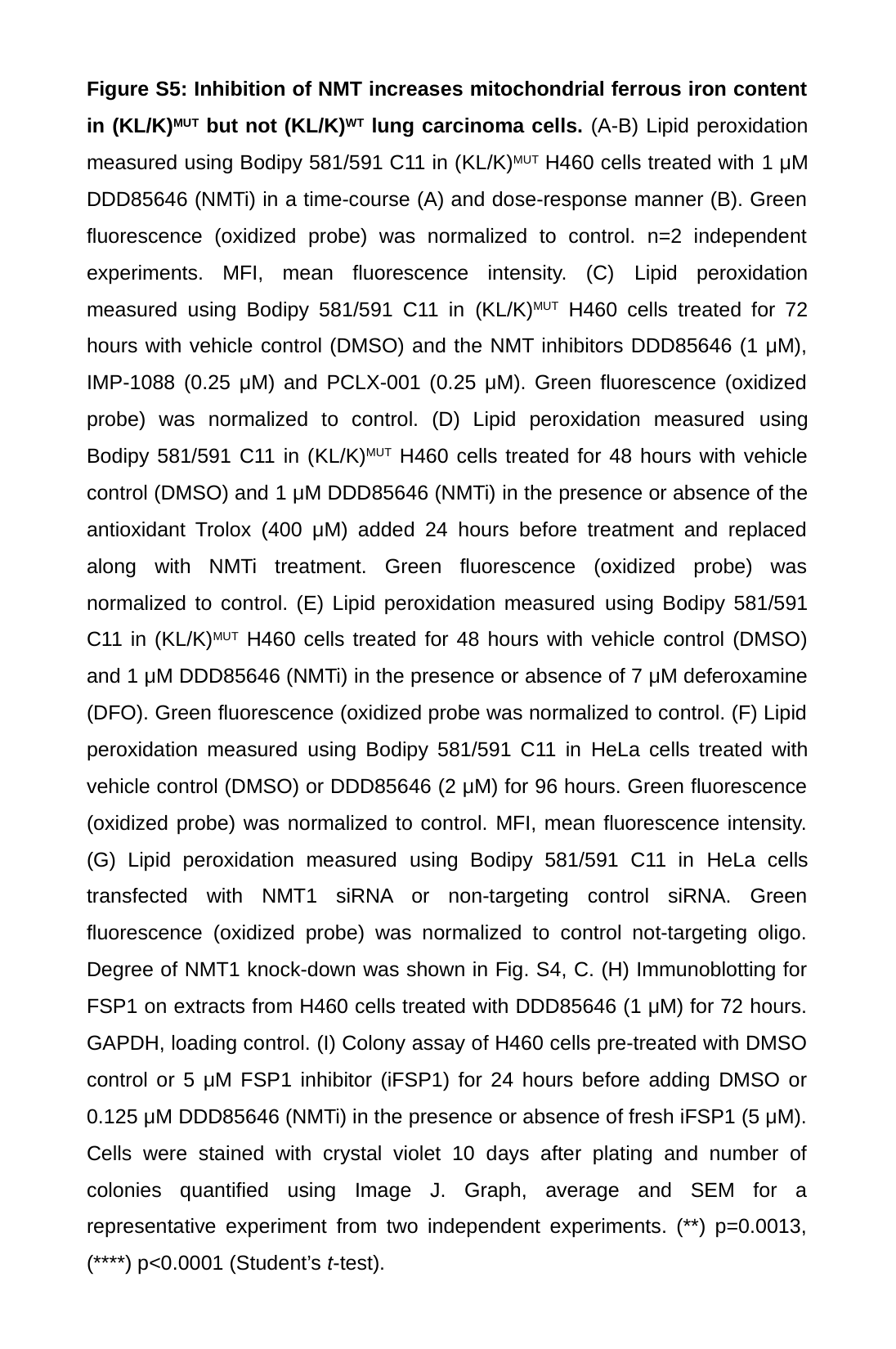

Figure S5: Inhibition of NMT increases mitochondrial ferrous iron content in (KL/K)MUT but not (KL/K)WT lung carcinoma cells. (A-B) Lipid peroxidation measured using Bodipy 581/591 C11 in (KL/K)MUT H460 cells treated with 1 μM DDD85646 (NMTi) in a time-course (A) and dose-response manner (B). Green fluorescence (oxidized probe) was normalized to control. n=2 independent experiments. MFI, mean fluorescence intensity. (C) Lipid peroxidation measured using Bodipy 581/591 C11 in (KL/K)MUT H460 cells treated for 72 hours with vehicle control (DMSO) and the NMT inhibitors DDD85646 (1 μM), IMP-1088 (0.25 μM) and PCLX-001 (0.25 μM). Green fluorescence (oxidized probe) was normalized to control. (D) Lipid peroxidation measured using Bodipy 581/591 C11 in (KL/K)MUT H460 cells treated for 48 hours with vehicle control (DMSO) and 1 μM DDD85646 (NMTi) in the presence or absence of the antioxidant Trolox (400 μM) added 24 hours before treatment and replaced along with NMTi treatment. Green fluorescence (oxidized probe) was normalized to control. (E) Lipid peroxidation measured using Bodipy 581/591 C11 in (KL/K)MUT H460 cells treated for 48 hours with vehicle control (DMSO) and 1 μM DDD85646 (NMTi) in the presence or absence of 7 μM deferoxamine (DFO). Green fluorescence (oxidized probe was normalized to control. (F) Lipid peroxidation measured using Bodipy 581/591 C11 in HeLa cells treated with vehicle control (DMSO) or DDD85646 (2 μM) for 96 hours. Green fluorescence (oxidized probe) was normalized to control. MFI, mean fluorescence intensity. (G) Lipid peroxidation measured using Bodipy 581/591 C11 in HeLa cells transfected with NMT1 siRNA or non-targeting control siRNA. Green fluorescence (oxidized probe) was normalized to control not-targeting oligo. Degree of NMT1 knock-down was shown in Fig. S4, C. (H) Immunoblotting for FSP1 on extracts from H460 cells treated with DDD85646 (1 μM) for 72 hours. GAPDH, loading control. (I) Colony assay of H460 cells pre-treated with DMSO control or 5 μM FSP1 inhibitor (iFSP1) for 24 hours before adding DMSO or 0.125 μM DDD85646 (NMTi) in the presence or absence of fresh iFSP1 (5 μM). Cells were stained with crystal violet 10 days after plating and number of colonies quantified using Image J. Graph, average and SEM for a representative experiment from two independent experiments. (**) p=0.0013, (****) p<0.0001 (Student’s t-test).
