# Supplementary material for: N-Myristoytransferase Inhibition Causes Mitochondrial Iron Overload and Parthanatos in TIM17A-Dependent Aggressive Lung Carcinoma
Source: Cancer Res Commun. 2024 Jul 25;4(7):1815–33. doi: 10.1158/2767-9764.CRC-23-0428 (PMC11270646; doi:10.1158/2767-9764.CRC-23-0428)
Supplement: Figure S6 — Inhibition of NMT prevents cell cycle progression and causes death in lung carcinoma cells. [file crc-23-0428_figure_s6_supps6.pptx]

## Slide 1
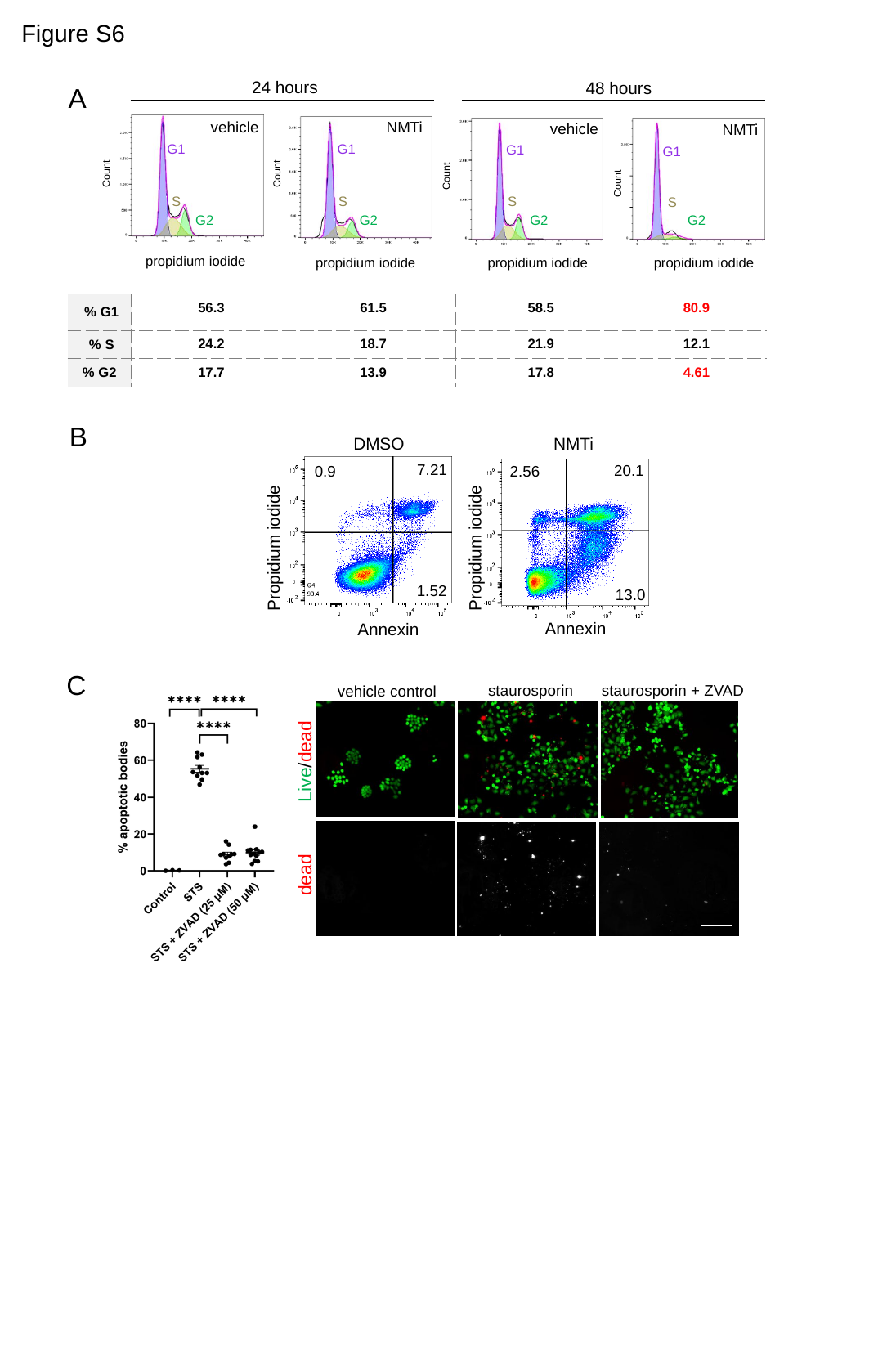

Figure S6
24 hours
48 hours
A
vehicle
Count
NMTi
vehicle
NMTi
G1
G1
G1
G1
Count
Count
Count
S
S
S
S
G2
G2
G2
G2
propidium iodide
propidium iodide
propidium iodide
propidium iodide
| % G1 | 56.3 | 61.5 | 58.5 | 80.9 |
| --- | --- | --- | --- | --- |
| % S | 24.2 | 18.7 | 21.9 | 12.1 |
| % G2 | 17.7 | 13.9 | 17.8 | 4.61 |
B
NMTi
20.1
2.56
13.0
Annexin
DMSO
7.21
0.9
Propidium iodide
Propidium iodide
1.52
Annexin
C
staurosporin + ZVAD
staurosporin
vehicle control
Live/dead
dead

## Slide 2
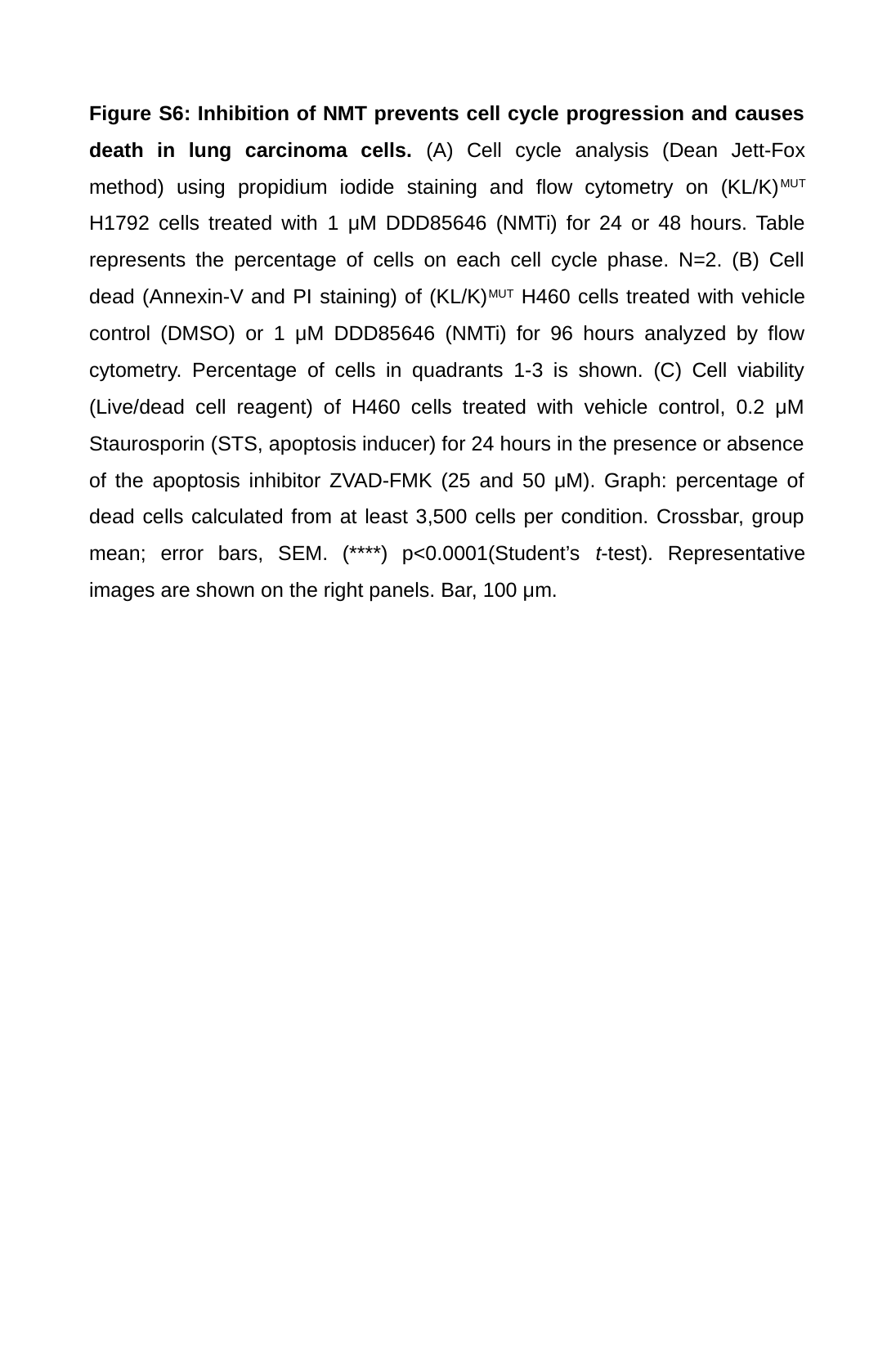

Figure S6: Inhibition of NMT prevents cell cycle progression and causes death in lung carcinoma cells. (A) Cell cycle analysis (Dean Jett-Fox method) using propidium iodide staining and flow cytometry on (KL/K)MUT H1792 cells treated with 1 μM DDD85646 (NMTi) for 24 or 48 hours. Table represents the percentage of cells on each cell cycle phase. N=2. (B) Cell dead (Annexin-V and PI staining) of (KL/K)MUT H460 cells treated with vehicle control (DMSO) or 1 μM DDD85646 (NMTi) for 96 hours analyzed by flow cytometry. Percentage of cells in quadrants 1-3 is shown. (C) Cell viability (Live/dead cell reagent) of H460 cells treated with vehicle control, 0.2 μM Staurosporin (STS, apoptosis inducer) for 24 hours in the presence or absence of the apoptosis inhibitor ZVAD-FMK (25 and 50 μM). Graph: percentage of dead cells calculated from at least 3,500 cells per condition. Crossbar, group mean; error bars, SEM. (****) p<0.0001(Student’s t-test). Representative images are shown on the right panels. Bar, 100 μm.
