# Supplementary material for: N-Myristoytransferase Inhibition Causes Mitochondrial Iron Overload and Parthanatos in TIM17A-Dependent Aggressive Lung Carcinoma
Source: Cancer Res Commun. 2024 Jul 25;4(7):1815–33. doi: 10.1158/2767-9764.CRC-23-0428 (PMC11270646; doi:10.1158/2767-9764.CRC-23-0428)
Supplement: Figure S7 — Treatment with necroptosis and pyroptosis inhibitors fail to prevent cell death induced by of NMTi treatment in lung carcinoma cells. [file crc-23-0428_figure_s7_supps7.pptx]

## Slide 1
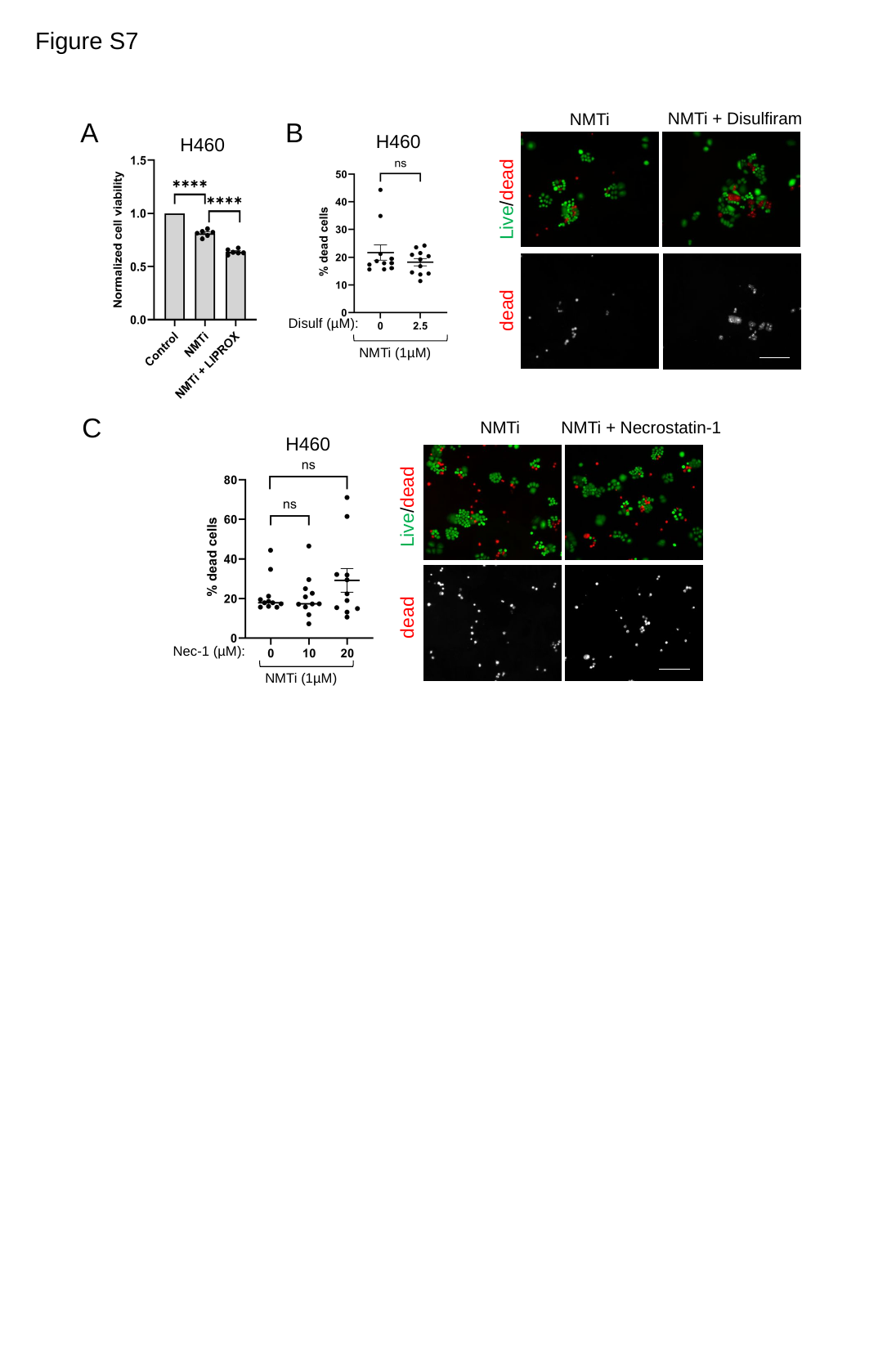

Figure S7
NMTi + Disulfiram
NMTi
B
A
H460
H460
Live/dead
dead
 Disulf (µM):
NMTi (1µM)
C
NMTi
NMTi + Necrostatin-1
H460
Live/dead
dead
NMTi (1µM)
 Nec-1 (µM):

## Slide 2
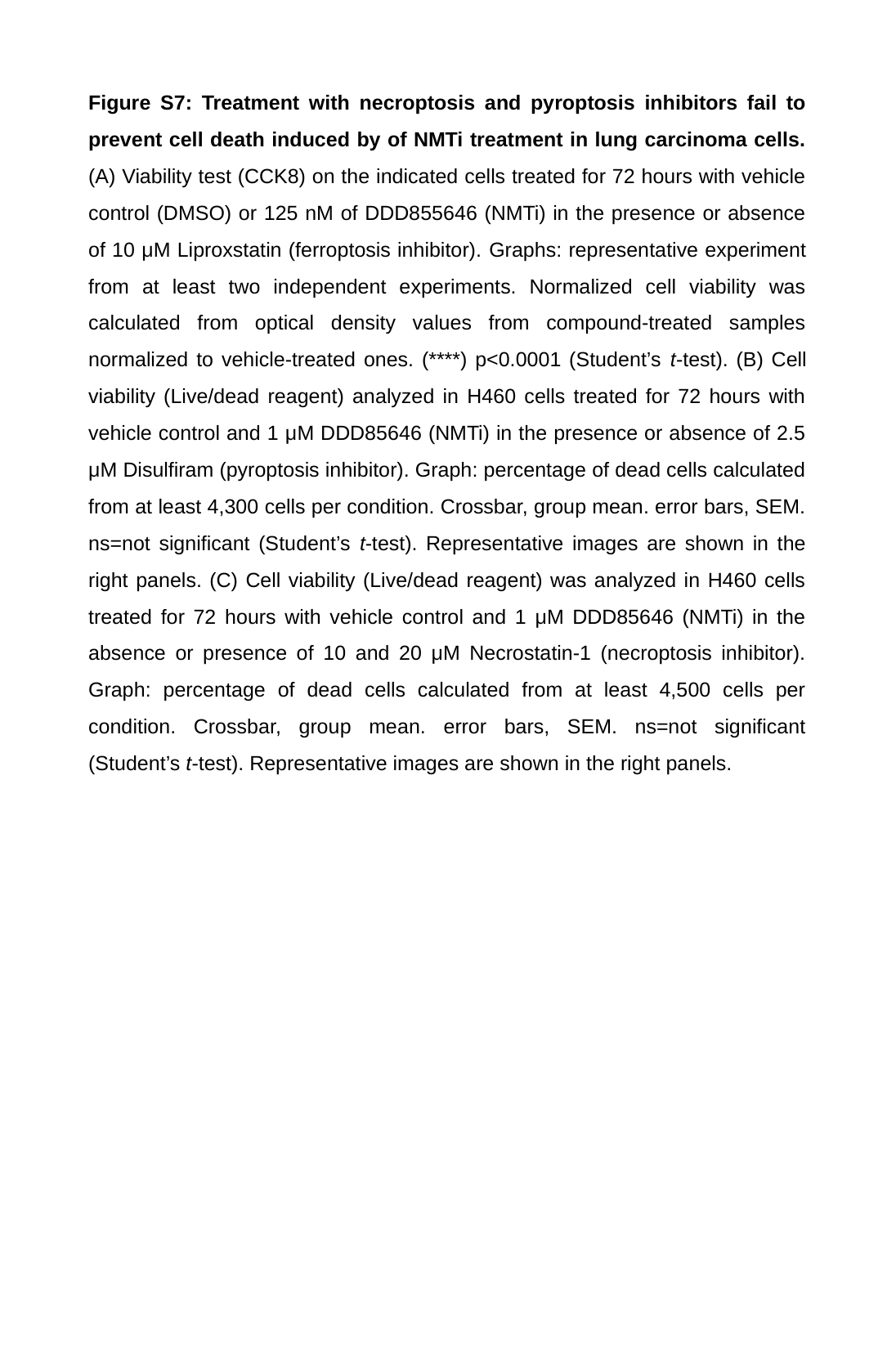

Figure S7: Treatment with necroptosis and pyroptosis inhibitors fail to prevent cell death induced by of NMTi treatment in lung carcinoma cells. (A) Viability test (CCK8) on the indicated cells treated for 72 hours with vehicle control (DMSO) or 125 nM of DDD855646 (NMTi) in the presence or absence of 10 μM Liproxstatin (ferroptosis inhibitor). Graphs: representative experiment from at least two independent experiments. Normalized cell viability was calculated from optical density values from compound-treated samples normalized to vehicle-treated ones. (****) p<0.0001 (Student’s t-test). (B) Cell viability (Live/dead reagent) analyzed in H460 cells treated for 72 hours with vehicle control and 1 μM DDD85646 (NMTi) in the presence or absence of 2.5 μM Disulfiram (pyroptosis inhibitor). Graph: percentage of dead cells calculated from at least 4,300 cells per condition. Crossbar, group mean. error bars, SEM. ns=not significant (Student’s t-test). Representative images are shown in the right panels. (C) Cell viability (Live/dead reagent) was analyzed in H460 cells treated for 72 hours with vehicle control and 1 μM DDD85646 (NMTi) in the absence or presence of 10 and 20 μM Necrostatin-1 (necroptosis inhibitor). Graph: percentage of dead cells calculated from at least 4,500 cells per condition. Crossbar, group mean. error bars, SEM. ns=not significant (Student’s t-test). Representative images are shown in the right panels.
