# Supplementary material for: N-Myristoytransferase Inhibition Causes Mitochondrial Iron Overload and Parthanatos in TIM17A-Dependent Aggressive Lung Carcinoma
Source: Cancer Res Commun. 2024 Jul 25;4(7):1815–33. doi: 10.1158/2767-9764.CRC-23-0428 (PMC11270646; doi:10.1158/2767-9764.CRC-23-0428)
Supplement: Figure S8 — NMTi treatment induces features of parthanatos in vitro and in vivo. [file crc-23-0428_figure_s8_supps8.pptx]

## Slide 1
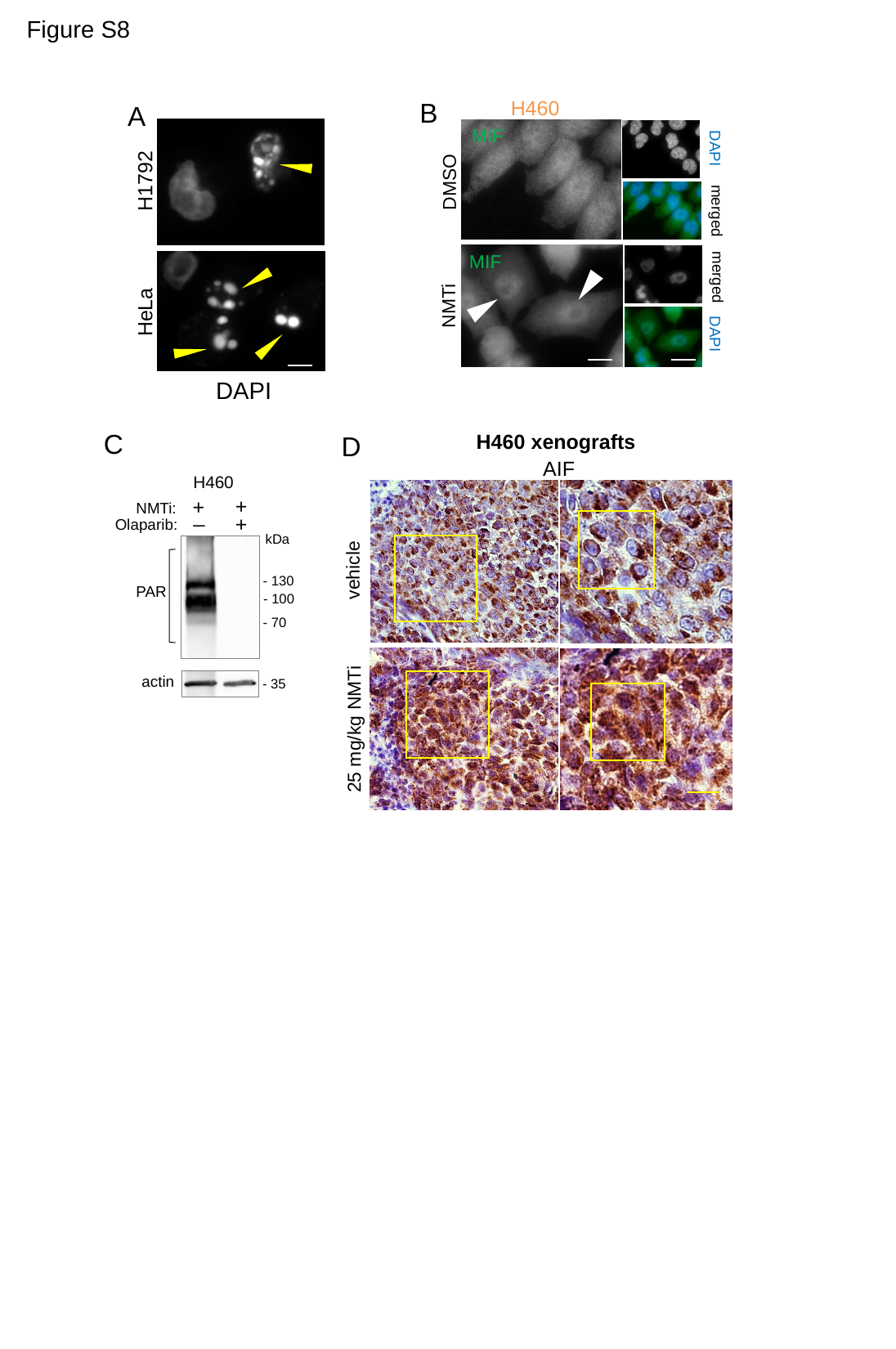

Figure S8
H460
B
A
H1792
HeLa
DAPI
MIF
DAPI
DMSO
merged
MIF
merged
NMTi
DAPI
C
H460 xenografts
D
AIF
H460
+
+
NMTi:
_
+
Olaparib:
kDa
- 130
PAR
- 100
- 70
actin
- 35
vehicle
25 mg/kg NMTi

## Slide 2
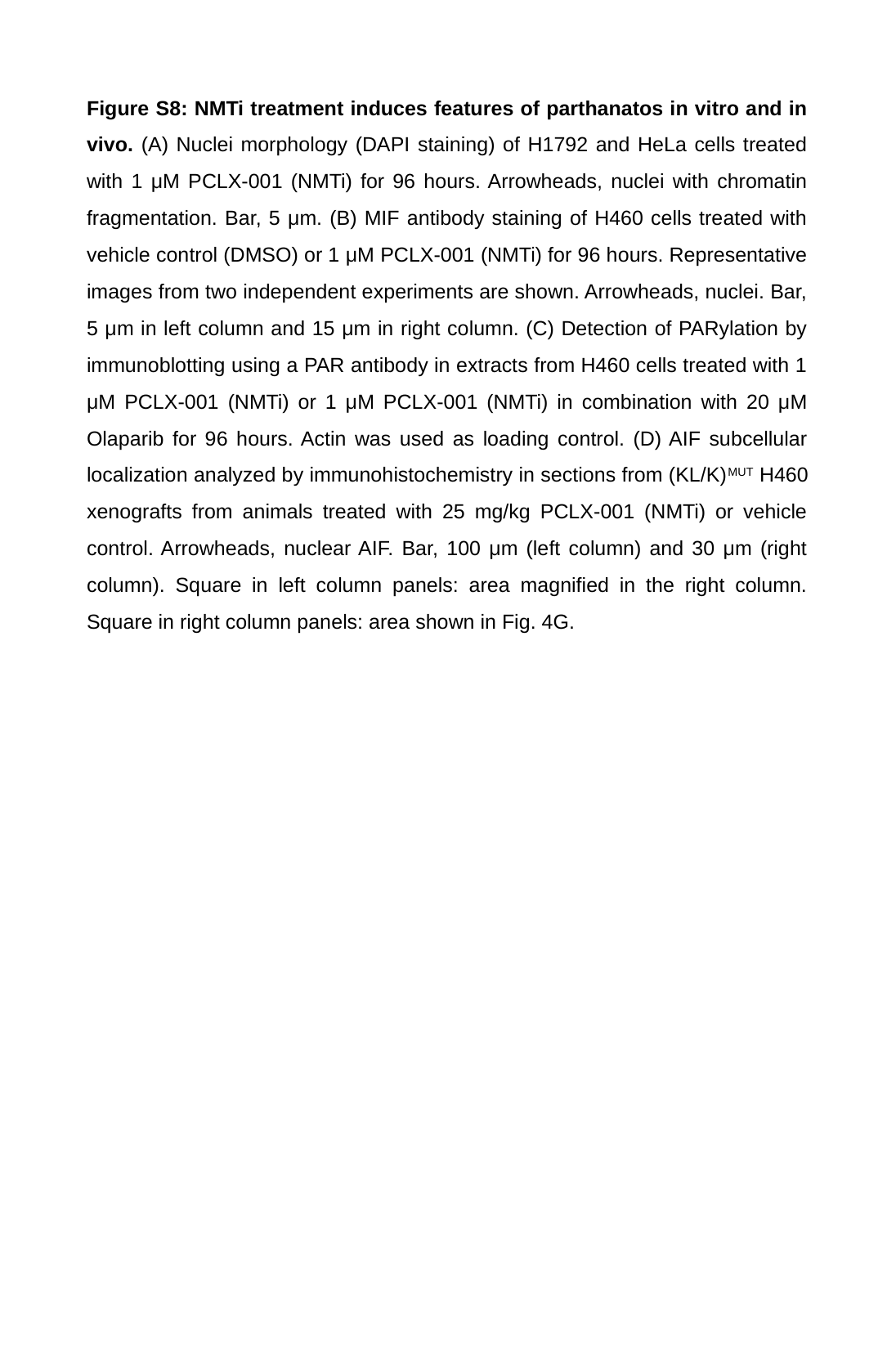

Figure S8: NMTi treatment induces features of parthanatos in vitro and in vivo. (A) Nuclei morphology (DAPI staining) of H1792 and HeLa cells treated with 1 μM PCLX-001 (NMTi) for 96 hours. Arrowheads, nuclei with chromatin fragmentation. Bar, 5 μm. (B) MIF antibody staining of H460 cells treated with vehicle control (DMSO) or 1 μM PCLX-001 (NMTi) for 96 hours. Representative images from two independent experiments are shown. Arrowheads, nuclei. Bar, 5 μm in left column and 15 μm in right column. (C) Detection of PARylation by immunoblotting using a PAR antibody in extracts from H460 cells treated with 1 μM PCLX-001 (NMTi) or 1 μM PCLX-001 (NMTi) in combination with 20 μM Olaparib for 96 hours. Actin was used as loading control. (D) AIF subcellular localization analyzed by immunohistochemistry in sections from (KL/K)MUT H460 xenografts from animals treated with 25 mg/kg PCLX-001 (NMTi) or vehicle control. Arrowheads, nuclear AIF. Bar, 100 μm (left column) and 30 μm (right column). Square in left column panels: area magnified in the right column. Square in right column panels: area shown in Fig. 4G.
