# Supplementary material for: N-Myristoytransferase Inhibition Causes Mitochondrial Iron Overload and Parthanatos in TIM17A-Dependent Aggressive Lung Carcinoma
Source: Cancer Res Commun. 2024 Jul 25;4(7):1815–33. doi: 10.1158/2767-9764.CRC-23-0428 (PMC11270646; doi:10.1158/2767-9764.CRC-23-0428)
Supplement: Figure S9 — The PARP inhibitor olaparib rescues death induced by NMTi treatment. [file crc-23-0428_figure_s9_supps9.pptx]

## Slide 1
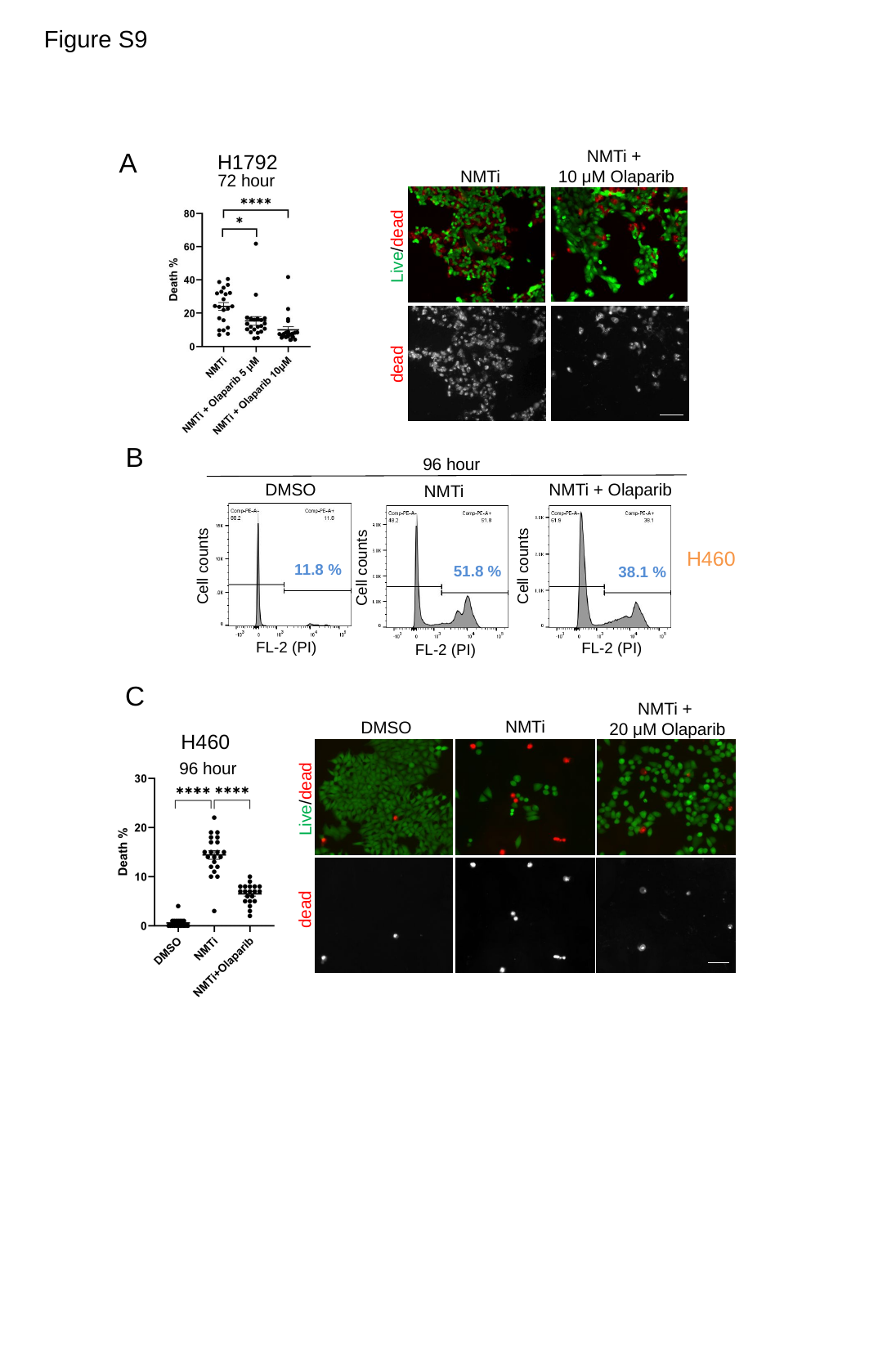

Figure S9
NMTi +
10 μM Olaparib
NMTi
Live/dead
dead
H1792
72 hour
A
B
96 hour
DMSO
NMTi + Olaparib
NMTi
11.8 %
Cell counts
FL-2 (PI)
51.8 %
Cell counts
FL-2 (PI)
38.1 %
Cell counts
FL-2 (PI)
H460
C
NMTi +
20 μM Olaparib
NMTi
DMSO
Live/dead
dead
H460
96 hour

## Slide 2
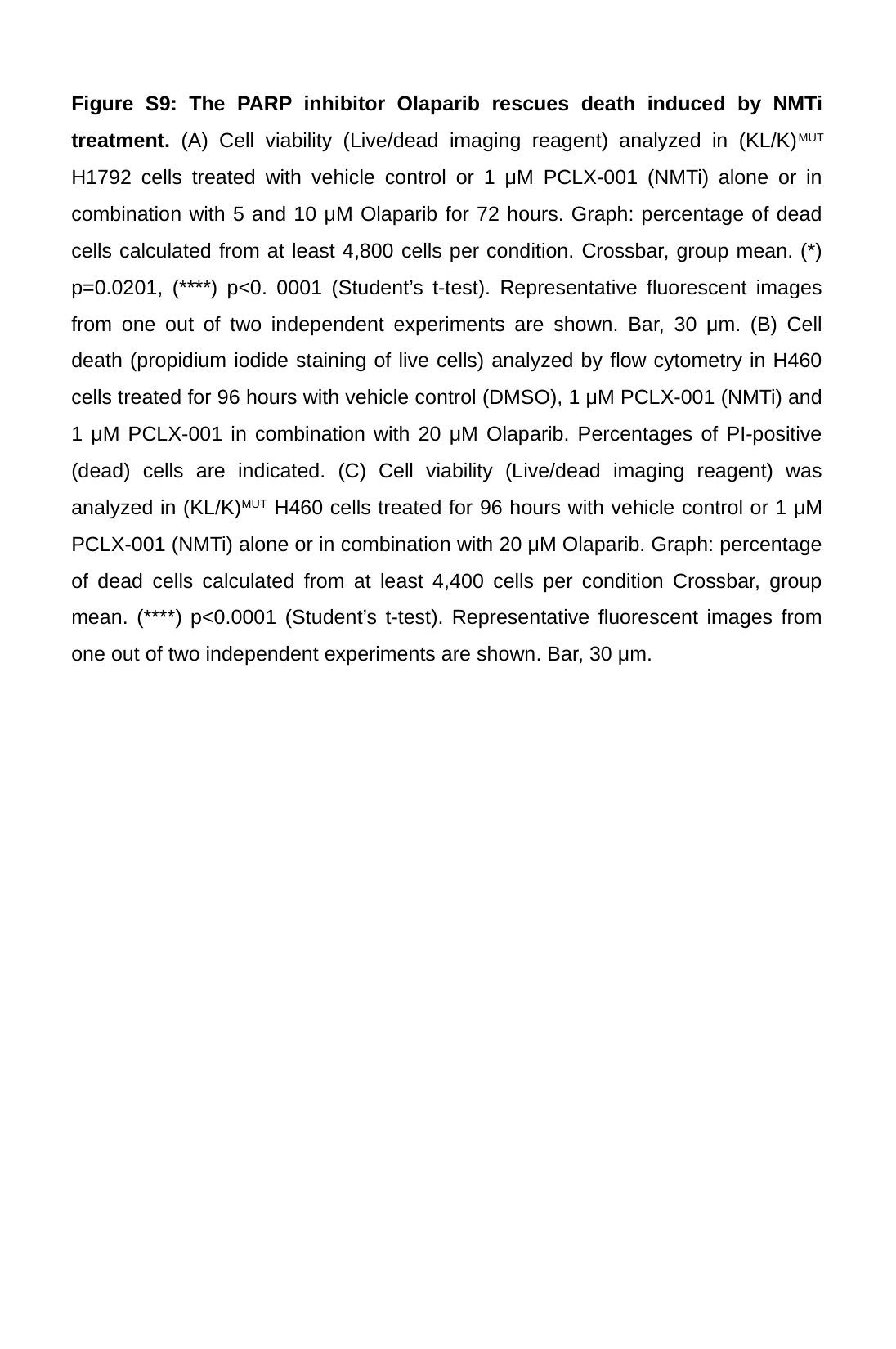

Figure S9: The PARP inhibitor Olaparib rescues death induced by NMTi treatment. (A) Cell viability (Live/dead imaging reagent) analyzed in (KL/K)MUT H1792 cells treated with vehicle control or 1 μM PCLX-001 (NMTi) alone or in combination with 5 and 10 μM Olaparib for 72 hours. Graph: percentage of dead cells calculated from at least 4,800 cells per condition. Crossbar, group mean. (*) p=0.0201, (****) p<0. 0001 (Student’s t-test). Representative fluorescent images from one out of two independent experiments are shown. Bar, 30 μm. (B) Cell death (propidium iodide staining of live cells) analyzed by flow cytometry in H460 cells treated for 96 hours with vehicle control (DMSO), 1 μM PCLX-001 (NMTi) and 1 μM PCLX-001 in combination with 20 μM Olaparib. Percentages of PI-positive (dead) cells are indicated. (C) Cell viability (Live/dead imaging reagent) was analyzed in (KL/K)MUT H460 cells treated for 96 hours with vehicle control or 1 μM PCLX-001 (NMTi) alone or in combination with 20 μM Olaparib. Graph: percentage of dead cells calculated from at least 4,400 cells per condition Crossbar, group mean. (****) p<0.0001 (Student’s t-test). Representative fluorescent images from one out of two independent experiments are shown. Bar, 30 μm.
