# Supplementary material for: N-Myristoytransferase Inhibition Causes Mitochondrial Iron Overload and Parthanatos in TIM17A-Dependent Aggressive Lung Carcinoma
Source: Cancer Res Commun. 2024 Jul 25;4(7):1815–33. doi: 10.1158/2767-9764.CRC-23-0428 (PMC11270646; doi:10.1158/2767-9764.CRC-23-0428)
Supplement: Figure S10 — Mitochondria is a key target of NMT inhibitors. [file crc-23-0428_figure_s10_supps10.pptx]

## Slide 1
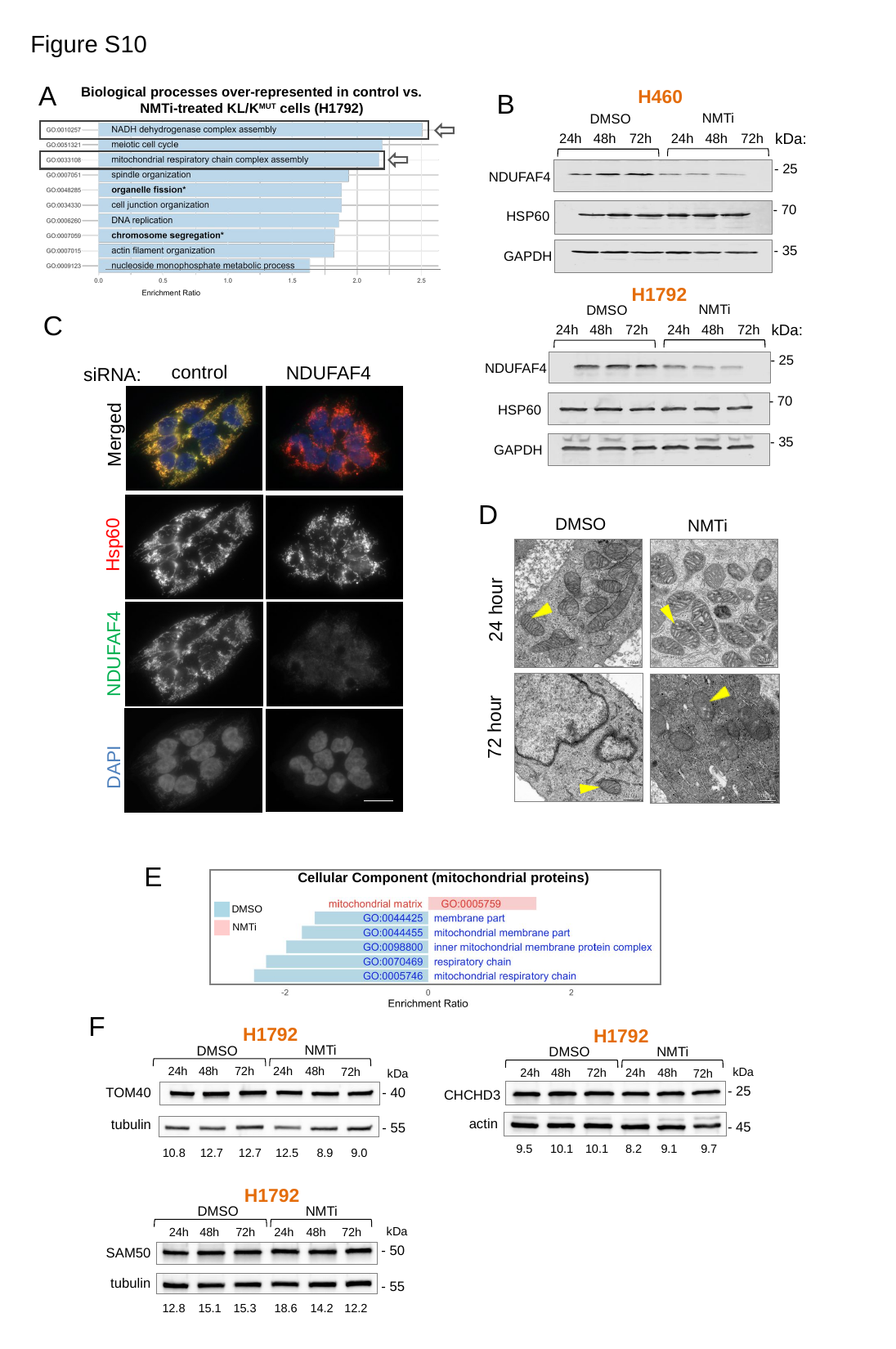

Figure S10
A
Biological processes over-represented in control vs. NMTi-treated KL/KMUT cells (H1792)
 H460
NMTi
DMSO
kDa:
24h
48h
72h
24h
48h
72h
- 25
NDUFAF4
- 70
HSP60
- 35
GAPDH
B
H1792
NMTi
DMSO
kDa:
24h
48h
72h
24h
48h
72h
- 25
NDUFAF4
- 70
HSP60
- 35
GAPDH
C
control
NDUFAF4
Merged
Hsp60
NDUFAF4
DAPI
siRNA:
D
DMSO
NMTi
24 hour
72 hour
E
Cellular Component (mitochondrial proteins)
DMSO
NMTi
F
H1792
H1792
NMTi
DMSO
24h
48h
72h
24h
48h
72h
kDa
- 40
TOM40
tubulin
- 55
12.5
9.0
10.8
12.7
12.7
8.9
NMTi
DMSO
kDa
24h
48h
72h
24h
48h
72h
- 25
CHCHD3
actin
- 45
9.5
10.1
10.1
8.2
9.1
9.7
 H1792
NMTi
DMSO
24h
48h
72h
24h
48h
72h
SAM50
tubulin
kDa
- 50
- 55
12.8
15.3
18.6
14.2
12.2
15.1

## Slide 2
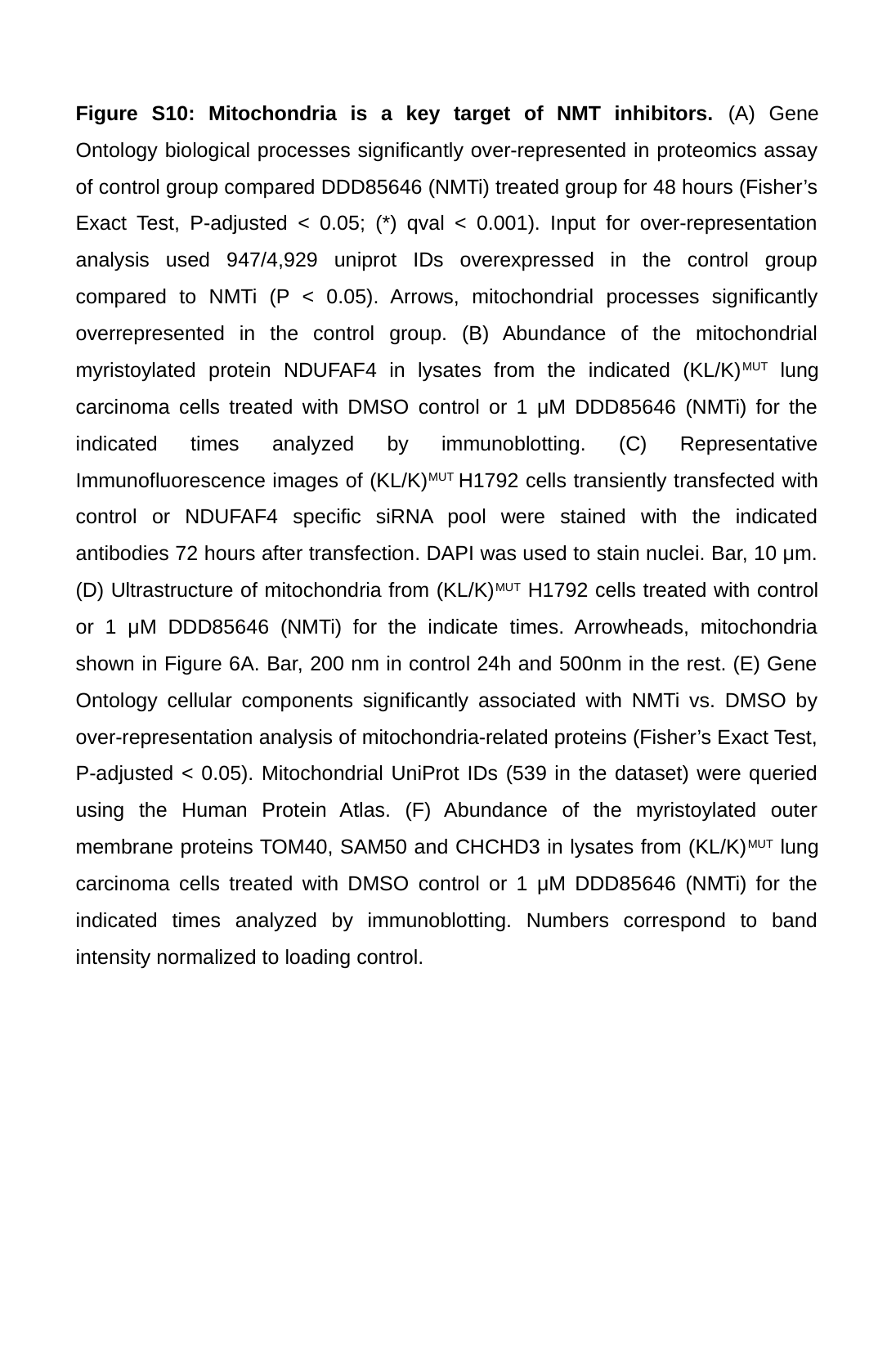

Figure S10: Mitochondria is a key target of NMT inhibitors. (A) Gene Ontology biological processes significantly over-represented in proteomics assay of control group compared DDD85646 (NMTi) treated group for 48 hours (Fisher’s Exact Test, P-adjusted < 0.05; (*) qval < 0.001). Input for over-representation analysis used 947/4,929 uniprot IDs overexpressed in the control group compared to NMTi (P < 0.05). Arrows, mitochondrial processes significantly overrepresented in the control group. (B) Abundance of the mitochondrial myristoylated protein NDUFAF4 in lysates from the indicated (KL/K)MUT lung carcinoma cells treated with DMSO control or 1 μM DDD85646 (NMTi) for the indicated times analyzed by immunoblotting. (C) Representative Immunofluorescence images of (KL/K)MUT H1792 cells transiently transfected with control or NDUFAF4 specific siRNA pool were stained with the indicated antibodies 72 hours after transfection. DAPI was used to stain nuclei. Bar, 10 μm. (D) Ultrastructure of mitochondria from (KL/K)MUT H1792 cells treated with control or 1 μM DDD85646 (NMTi) for the indicate times. Arrowheads, mitochondria shown in Figure 6A. Bar, 200 nm in control 24h and 500nm in the rest. (E) Gene Ontology cellular components significantly associated with NMTi vs. DMSO by over-representation analysis of mitochondria-related proteins (Fisher’s Exact Test, P-adjusted < 0.05). Mitochondrial UniProt IDs (539 in the dataset) were queried using the Human Protein Atlas. (F) Abundance of the myristoylated outer membrane proteins TOM40, SAM50 and CHCHD3 in lysates from (KL/K)MUT lung carcinoma cells treated with DMSO control or 1 μM DDD85646 (NMTi) for the indicated times analyzed by immunoblotting. Numbers correspond to band intensity normalized to loading control.
