# Supplementary material for: N-Myristoytransferase Inhibition Causes Mitochondrial Iron Overload and Parthanatos in TIM17A-Dependent Aggressive Lung Carcinoma
Source: Cancer Res Commun. 2024 Jul 25;4(7):1815–33. doi: 10.1158/2767-9764.CRC-23-0428 (PMC11270646; doi:10.1158/2767-9764.CRC-23-0428)
Supplement: Figure S11 — Effect of NMTi on the abundance of TIM17A in lung carcinoma cells. [file crc-23-0428_figure_s11_supps11.pptx]

## Slide 1
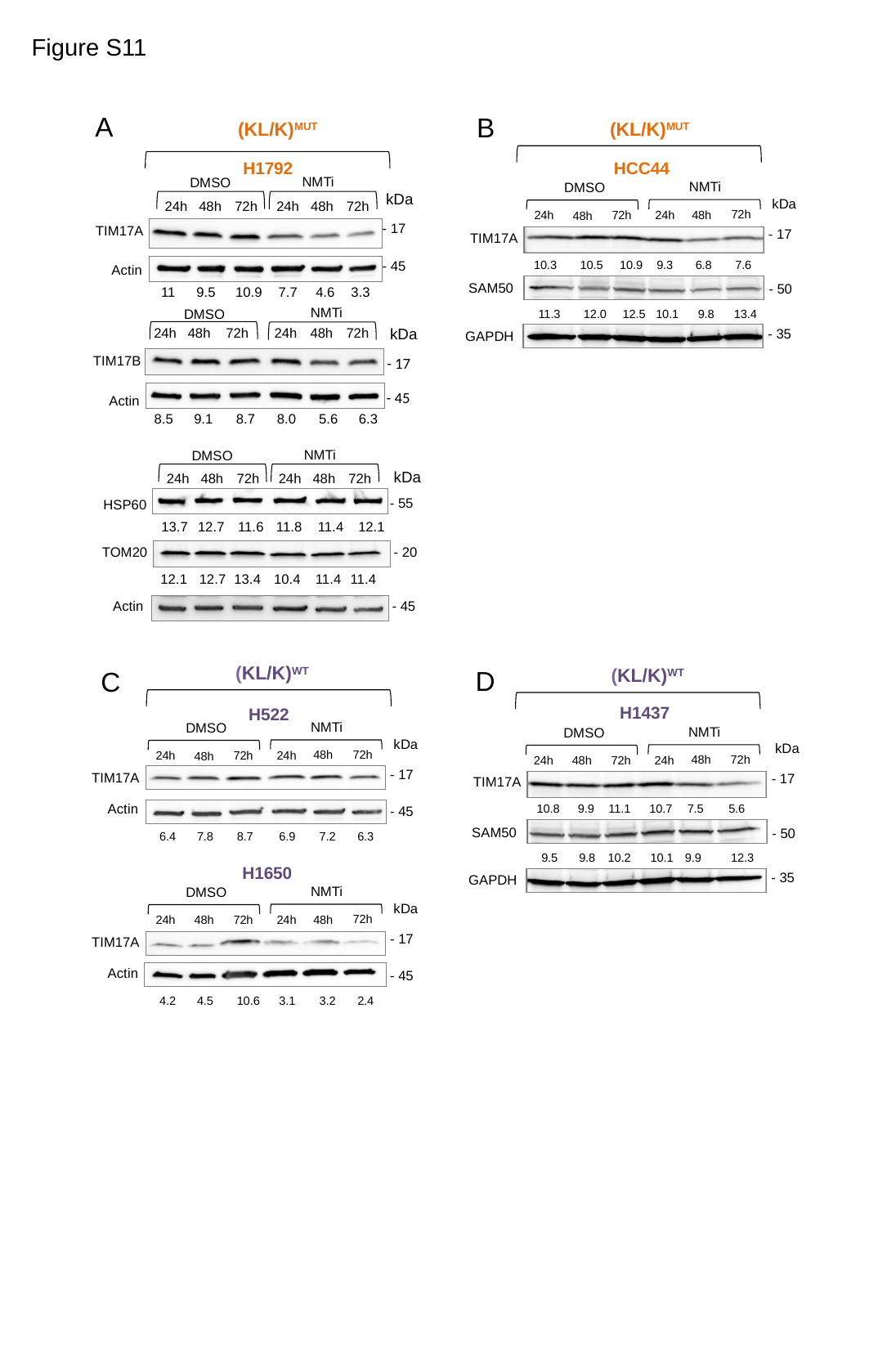

Figure S11
A
B
 (KL/K)MUT
 (KL/K)MUT
H1792
HCC44
NMTi
DMSO
kDa
24h
48h
72h
24h
48h
72h
- 17
TIM17A
- 45
Actin
11
9.5
10.9
7.7
4.6
3.3
NMTi
DMSO
kDa
72h
48h
72h
24h
24h
48h
- 17
TIM17A
10.3
10.5
10.9
9.3
6.8
7.6
6.4
7.8
8.7
6.9
7.2
6.3
SAM50
- 50
11.3
12.0
12.5
10.1
9.8
13.4
- 35
GAPDH
NMTi
DMSO
24h
48h
72h
24h
48h
72h
kDa
TIM17B
- 17
- 45
Actin
8.5
9.1
8.7
8.0
5.6
6.3
NMTi
DMSO
kDa
24h
48h
72h
24h
48h
72h
- 55
HSP60
13.7
12.7
11.6
11.8
11.4
12.1
- 20
TOM20
11.4
12.1
12.7
13.4
10.4
11.4
Actin
- 45
 (KL/K)WT
 (KL/K)WT
D
C
H1437
H522
NMTi
DMSO
72h
48h
72h
24h
24h
48h
6.4
7.8
8.7
6.9
7.2
6.3
kDa
- 17
TIM17A
Actin
- 45
NMTi
DMSO
kDa
72h
48h
72h
24h
24h
48h
- 17
TIM17A
10.8
9.9
11.1
10.7
7.5
5.6
6.4
7.8
8.7
6.9
7.2
6.3
SAM50
- 50
9.5
9.8
10.2
10.1
9.9
12.3
- 35
GAPDH
H1650
NMTi
DMSO
kDa
72h
48h
72h
24h
24h
48h
- 17
TIM17A
Actin
- 45
4.2
4.5
10.6
3.1
3.2
2.4

## Slide 2
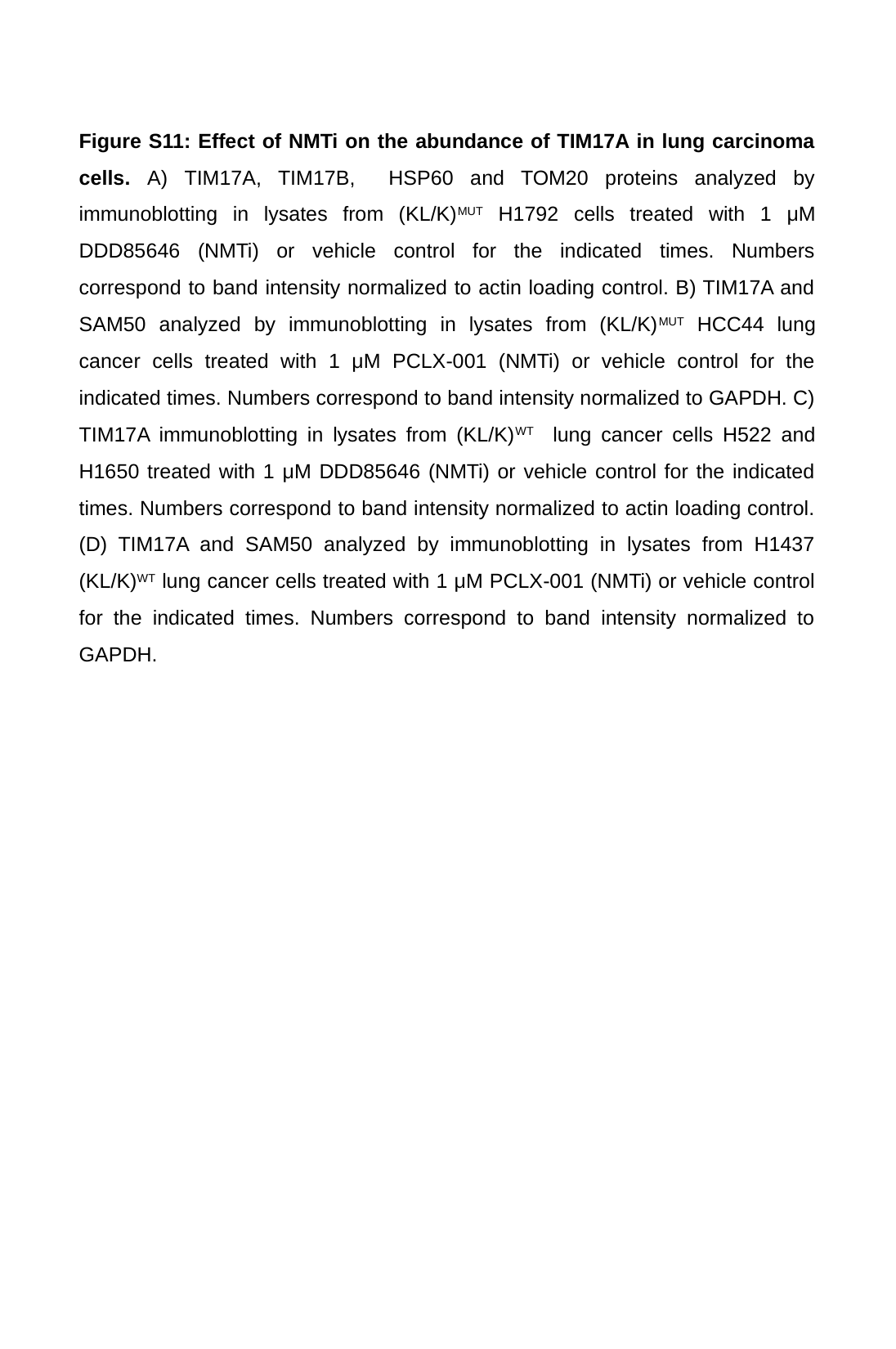

Figure S11: Effect of NMTi on the abundance of TIM17A in lung carcinoma cells. A) TIM17A, TIM17B, HSP60 and TOM20 proteins analyzed by immunoblotting in lysates from (KL/K)MUT H1792 cells treated with 1 μM DDD85646 (NMTi) or vehicle control for the indicated times. Numbers correspond to band intensity normalized to actin loading control. B) TIM17A and SAM50 analyzed by immunoblotting in lysates from (KL/K)MUT HCC44 lung cancer cells treated with 1 μM PCLX-001 (NMTi) or vehicle control for the indicated times. Numbers correspond to band intensity normalized to GAPDH. C) TIM17A immunoblotting in lysates from (KL/K)WT lung cancer cells H522 and H1650 treated with 1 μM DDD85646 (NMTi) or vehicle control for the indicated times. Numbers correspond to band intensity normalized to actin loading control. (D) TIM17A and SAM50 analyzed by immunoblotting in lysates from H1437 (KL/K)WT lung cancer cells treated with 1 μM PCLX-001 (NMTi) or vehicle control for the indicated times. Numbers correspond to band intensity normalized to GAPDH.
