# Supplementary material for: N-Myristoytransferase Inhibition Causes Mitochondrial Iron Overload and Parthanatos in TIM17A-Dependent Aggressive Lung Carcinoma
Source: Cancer Res Commun. 2024 Jul 25;4(7):1815–33. doi: 10.1158/2767-9764.CRC-23-0428 (PMC11270646; doi:10.1158/2767-9764.CRC-23-0428)
Supplement: Figure S12 — Effect of lipid peroxidation on the abundance of TIM17A in lung carcinoma cells. [file crc-23-0428_figure_s12_supps12.pptx]

## Slide 1
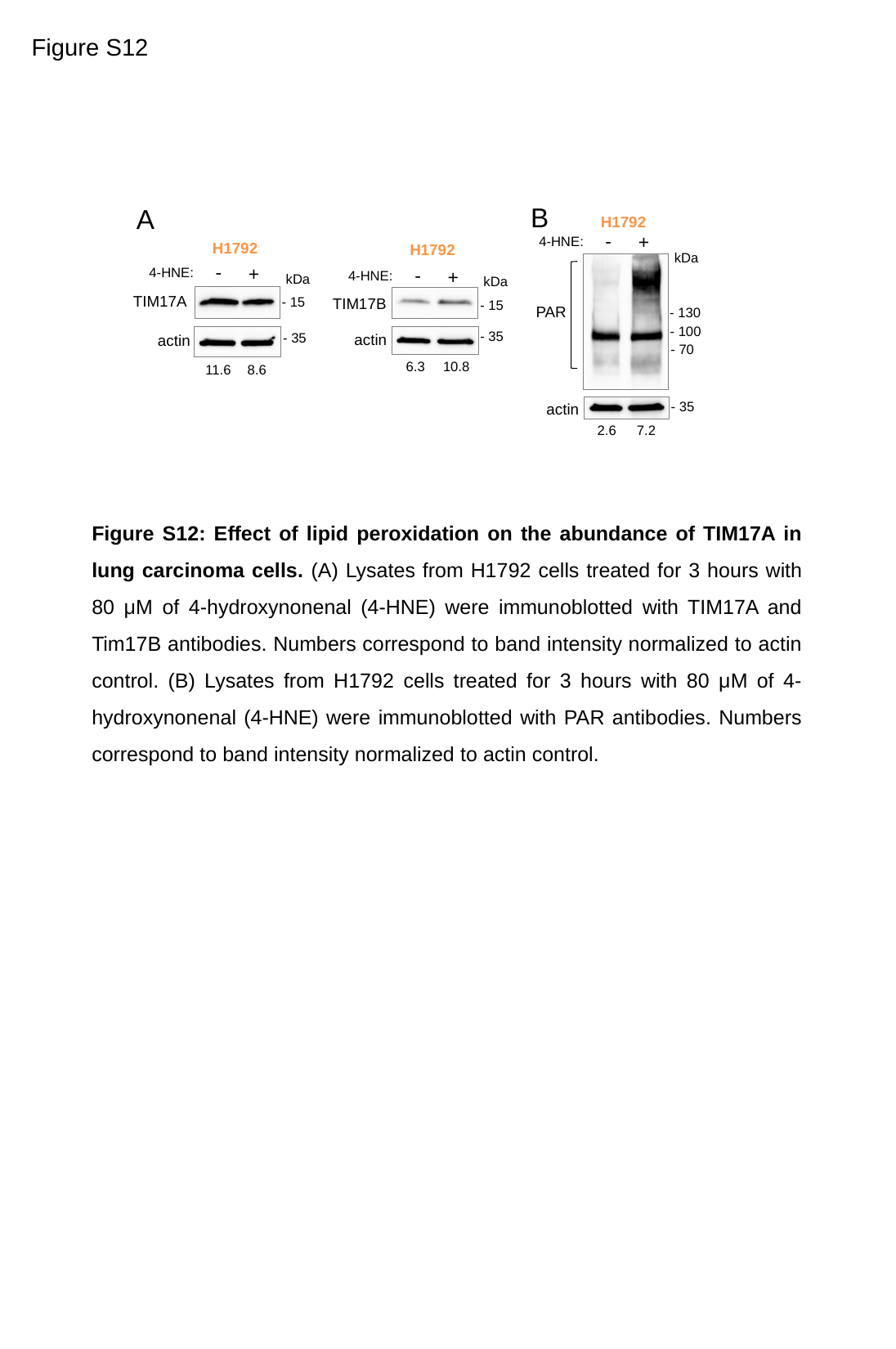

Figure S12
B
A
H1792
-
+
4-HNE:
H1792
H1792
kDa
-
+
-
+
4-HNE:
4-HNE:
kDa
kDa
TIM17A
- 15
TIM17B
- 15
PAR
- 130
- 100
- 35
- 35
actin
actin
- 70
10.8
6.3
11.6
8.6
- 35
actin
2.6
7.2
Figure S12: Effect of lipid peroxidation on the abundance of TIM17A in lung carcinoma cells. (A) Lysates from H1792 cells treated for 3 hours with 80 μM of 4-hydroxynonenal (4-HNE) were immunoblotted with TIM17A and Tim17B antibodies. Numbers correspond to band intensity normalized to actin control. (B) Lysates from H1792 cells treated for 3 hours with 80 μM of 4-hydroxynonenal (4-HNE) were immunoblotted with PAR antibodies. Numbers correspond to band intensity normalized to actin control.
